# Comparative efficacy of eight traditional Chinese medicines combined with statins in the treatment of hyperlipidemia: a Bayesian network meta-analysis

**DOI:** 10.3389/fphar.2025.1614767

**Published:** 2025-08-05

**Authors:** Ling Jiang, Wen Fan, Fangyu Zhou, Lihan Liu, Maoxing Pan, Qinhe Yang, Yupei Zhang

**Affiliations:** ^1^ School of Chinese Medicine, Jinan University, Guangzhou, China; ^2^ Department of Traditional Chinese Medicine, Xiangdong Hospital Affiliated to Hunan Normal University, Liling, China; ^3^ Department of Traditional Chinese Medicine, The First Affiliated Hospital of Jinan University, Guangzhou, China

**Keywords:** traditional Chinese medicines, hyperlipidemia, network meta-analysis, clinical efficacy, safety outcome

## Abstract

**Background:**

Hyperlipidemia drives global cardiovascular mortality by elevating risks of atherosclerosis and stroke. While statins are foundational, traditional Chinese medicines (TCMs) are widely combined with statins to boost efficacy. However, diverse TCM formulations lack comparative evidence in combination regimens, necessitating urgent evidence-based optimization.

**Methods:**

Based on preliminary literature review and component usage frequency analysis, 8 TCMs were included firstly. Then, we conducted a systematic search for RCTs that assessed 8 TCMs with traditional statin drugs (TT) for the treatment of hyperlipidemia. The search was conducted through September 30, 2024, and encompassed China National Knowledge Infrastructure (CNKI), Chinese Biomedical Literature Database (CBM), Database of Chinese Sci-Tech Periodicals (VIP), Wanfang Database, PubMed, Web of Science, and Cochrane Library. Outcomes included clinical total effective rate, total cholesterol (TC), triglycerides (TG), high-density lipoprotein cholesterol (HDL-C), and low-density lipoprotein cholesterol (LDL-C). Risk of bias in RCTs was evaluated using Cochrane’s bias risk tool. Evidence synthesis was performed utilizing both direct and Bayesian network meta-analyses (NMA). Meta-regression analysis, subgroup analysis, publication bias analysis, and sensitivity analysis were employed to evaluate heterogeneity sources and efficacy robustness. Ranking analysis was implemented to comparatively assess clinical efficacy among eight TCMs. Evidence quality for each outcome was assessed using Grading of Recommendations, Assessment, Development, and Evaluation approach (GRADE). Overall, the proposed structured framework integrated high-frequency TCM screening, NMA-driven efficacy ranking, methodological validation, and mechanistic investigation to holistically evaluate therapeutic interventions for the first time.

**Results:**

67 RCTs involving 7327 individuals and 8 TCMs were encompassed. Related analyses indicated TT + TCMs were more efficacious than TT monotherapy. Among 8 TCMs + TT, TT combined with Jiangzhi Tongmai Capsule (TT + JZTM) demonstrated the highest clinical total effective rate, TT combined with Dantian Jiangzhi Pill (TT + DTJZ) was the most effective in reducing TC, TT combined with Pushen Capsule (TT + PS) was the most effective in reducing TG, and TT combined with Jiangzhiling Tablet (TT + JZL) was the most effective in increasing HDL-C and reducing LDL-C.

**Conclusion:**

NMA revealed the overall clinical efficacy of TT + JZL, TT + DTJZ, and TT + JZTM were ranked at the forefront in treating hyperlipidemia. These findings provide evidence-based guidance for tailoring TCM-statin combinations to target individualized lipid profiles.

**Systematic Review Registration:**

https://www.crd.york.ac.uk/PROSPERO/view/CRD42024603979, identifier [CRD42024603979].

## 1 Introduction

Hyperlipidemia, being a critical risk factor, significantly exacerbates the risk of severe health complications such as atherosclerosis, myocardial infarction, and stroke. Approximately 17 million deaths annually due to cardiovascular diseases raised by hyperlipidemia, accounting for 30% of total global mortality (WHO CVD Risk Chart Working Group, 2019). In 2019, high LDL-C index was implicated in approximately 3.78 million deaths from ischemic heart disease (IHD), accounting for 44.3% of all IHD mortalities (Cieza et al., 2021). Hyperlipidemia is caused by disorders in lipid metabolism (Joint Committee, 2016) and common indicators include the elevation of TC, TG, and LDL-C, as well as the reduction of HDL-C (Li J. J. et al., 2023). It often presents no obvious symptoms at onset, making it difficult to recognize and highly concealed.

The current treatment of hyperlipidemia in biomedicine mainly relies on statins, which work by inhibiting cholesterol synthesis and accelerating the clearance of LDL. In China, statins are commonly co-administered with TCMs to enhance therapeutic effectiveness. Chinese patent medicine treatment adheres to the principles of holistic thinking and syndrome differentiation, aiming to improve lipid-lowering effects while ensuring the safety and non-toxic side effects. TCMs, predominantly composed of botanical drugs, also incorporate animal-derived substances, mineral-based components, and microbial fermentation products. Due to their well-defined compositions and established therapeutic efficacy, these formulations are widely employed in China for the management of hyperlipidemia. However, there are many kinds of TCMs available on the market, and the relative efficacy of various TCMs combined with TT for lipid regulation are not yet clear.

NMA integrates direct and indirect evidence to compare multiple treatments, enhancing statistical power where head-to-head trials are scarce. We applied Bayesian NMA to evaluate TCMs combined with statins for hyperlipidemia. Bayesian methods were prioritized due to their ability to (1) model sparse data efficiently, (2) generate probabilistic rankings for clinical utility, (3) address heterogeneity (e.g., statin types, patient profiles), and (4) quantify uncertainty via credible intervals. This approach provides actionable insights for guiding hyperlipidemia treatment when direct evidence is limited but comparative decisions are critical.

We systematically reviewed existing NMAs comparing the efficacy of TCMs for hyperlipidemia, identifying significant methodological limitations and incomplete evidence chains in current research. The work in Xu et al., 2022 focused on TCMs containing red yeast rice but only included three types, lacking heterogeneity analysis and sensitivity testing for individual medicines, which compromises conclusion robustness. The work in Yang et al., 2023 employed rigorous TCM selection for coronary heart disease with hyperlipidemia yet omitted essential heterogeneity assessments for each medicine, failing to address outcome variability sources. The research in Shi et al., 2025 compared multiple TCMs combined with atorvastatin and conducted detailed heterogeneity analyses. However, its TCM screening relied on direct specification from the Chinese Pharmacopoeia without systematic transparency, and omitted GRADE evaluation of evidence quality, undermining reliability of efficacy comparisons.

To address these prevalent methodological shortcomings and fragmented evidence chains, our study implements systematic enhancements. We first established the criteria for high-frequency TCM selection, then integrated pairwise and network meta-analyses to construct a robust evidence network. Subsequent meta-regression and subgroup analyses identified and mitigated heterogeneity sources, followed by sensitivity testing and GRADE application to evaluate evidence quality of key findings. This comprehensive methodology delivers a more balanced, reliable, and critically assessed evaluation of comparative TCM efficacy, addressing critical gaps in existing literature.

## 2 Materials and methods

Our study was carried out following the guidelines for PRISMA-NMA (Hutton et al., 2015) and PRISMA (Page et al., 2021), and their checklists were provided in Supplementary Material S1. Besides, the NMA study had been registered in the International Prospective Register of Systematic Reviews (PROSPERO: CRD42024603979). The aim of this study is to conduct a systematic assessment of the comparative clinical efficacy of various combinations of TCMs with TT for the treatment of hyperlipidemia.

Based on preliminary literature research as shown in Supplementary Material S2, a total of 11 frequently used TCMs were initially identified, including JZTM, DTJZ, PS, JZL, Hedan Tablet (HD), Jiangzhi Tongluo Soft Capsule (JZTL), Songling Xuemaikang Capsule (SLXMK), Xuezhikang Capsule (XZK), Xuezhitong Capsule (XZT), Zhibituo Tablet (ZBTT) and Zhibitai Capsule (ZBTC). Then, by the data analysis of high-frequency ingredients (Xu et al., 2024) and guided by the principles of maximizing coverage of high-frequency components while minimizing ingredient redundancy, 8 final candidates were selected through the following rationale:

Firstly, priority was given to TCMs containing more high-frequency components. Examples include PS (containing 7 high-frequency ingredients such as Crataegus Pinnatifida), DTJZ (with 5 components including Salvia Miltiorrhiza and Panax Notoginseng) and JZL (with 3 components including Cassia Seed), ensuring optimal utilization of clinically validated high-frequency ingredients. Secondly, for TCMs with overlapping components, those covering more high-frequency ingredients or demonstrating higher total frequency scores were retained. This rationale led to the exclusion of HD (duplicating Salvia Miltiorrhiza and Crataegus Pinnatifida with selected TCMs) to reduce therapeutic redundancy. Additionally, TCMs containing unique components, such as Monascus Purpureus in XZK and Allium Macrostemon in XZT, were preserved to enhance formula diversity. Strategic retention of low-frequency but critical components (e.g., Pueraria Lobata in SLXMK) ensured comprehensive therapeutic coverage.

The final selection of 8 TCMs (including DTJZ, JZL, JZTL, JZTM, PS, SLXMK, XZK and XTZ) achieved balanced coverage of high-frequency components while enhancing scientific validity through minimized redundancy and preserved unique constituents, ultimately optimizing clinical practicality. It should be noted that, while the original studies only provided the corresponding proprietary name and manufacturer, the detailed compositions can be determined by official records from the China National Medical Products Administration and corresponding manufacturers (accessible via their websites). Their detailed information of these TCMs were summarized in Supplementary Material S3.1, 3.21, including specific manufacturers, drug approval number, ingredients and processing method.

### 2.1 Inclusion and exclusion criteria

#### 2.1.1 Inclusion

We included published journal articles related to RCTs of TCMs for the treatment of hyperlipidemia or dyslipidemia. Patients with hyperlipidemia or dyslipidemia were included in the study, with no restrictions on age, gender, disease duration, or coexisting conditions. The criteria for diagnosing hyperlipidemia was derived from *the Chinese Guidelines for the Prevention and Treatment of Dyslipidemia in Adults* (Joint Committee, 2016).

Patients in the control group received TT, which comprised atorvastatin, rosuvastatin, simvastatin, pitavastatin, and fluvastatin. The treatment group, in addition to the control group’s regimen, was treated with one oral TCM approved for the treatment of dyslipidemia, including DTJZ, JZL, JZTL, JZTM, PS, SLXMK, XZK, and XZT. The dosage was according to the instructions, and the treatment period was no less than 2 weeks. The outcomes encompassed the clinical total effective rate, TC, TG, LDL-C, HDL-C, and adverse reactions. One or more outcomes should be reported in the included studies.

#### 2.1.2 Exclusion

The exclusion criteria for the literature included the following: (1) Non-RCTs, systematic reviews, meta-analyses, animal experiments, and reports based on expert experience, (2) Interventions that were non-pharmacological, such as acupuncture, acupoint application, (3) Studies that utilized traditional Chinese medicine decoctions, combine multiple TCMs in treatment, or employ unmarketed TCMs, (4) Studies with irrelevant content or topics that did not align with the theme, (5) Studies with duplicated data, unavailable original texts, or obvious errors, (6) Studies with outcomes that did not match the required indicators of this research.

The inclusion and exclusion criteria met the PICOS criteria as follows:

(1) Participants (P): Included patients met the diagnostic criteria of *the Chinese Guidelines for the Prevention and Treatment of Dyslipidemia in Adults (2016 Edition)* (TC/TG/LDL-C/HDL-C thresholds), excluding confounding factors such as hepatic or renal insufficiency to focus on the core pathophysiology of lipid metabolism disorders.
(2) Interventions and Comparisons (I/C): Patients in the control group received one conventional statin (e.g., atorvastatin, simvastatin, rosuvastatin, fluvastatin, pitavastatin, etc.), while the experimental group were treated with “TCM + statin” regimen. Drug dosages strictly followed prescribing instructions, with a treatment duration ≥2 weeks. Studies involving dose gradients or non-pharmacological interventions were excluded.
(3) Outcomes (O): Primary outcome was the total effective rate. Secondary outcomes included the lipid profiles (TC, TG, LDL-C, HDL-C) and adverse reactions. All included studies reported at least one core outcome. Among them, the total effective rate was evaluated in accordance with *the Chinese Guidelines for the Prevention and Treatment of Dyslipidemia in Adults.* Efficacy criteria were defined as: (a) Markedly Effective: resolution of clinical symptoms and signs accompanied by any one lipid improvement (TC reduction ≥20%, TG reduction ≥40%, HDL-C elevation ≥0.26 mmol/L, or TC/HDL-C ratio reduction ≥20%); (b) Effective: improvement in clinical symptoms and signs combined with any one lipid parameter change (TC reduction ≥10% but <20%, TG reduction ≥20% but <40%, HDL-C elevation ≥0.104 mmol/L but <0.26 mmol/L, or TC/HDL-C ratio reduction ≥10% but <20%); (c) Ineffective: failure to meet any lipid criterion with no significant post-treatment improvement in symptoms, signs, or lipid parameters. The total effective rate was calculated as (Number of Markedly Effective Cases + Number of Effective Cases)/Total Sample Size.
(4) Study Design (S): Prospective RCTs were included, while systematic reviews, animal studies, and non-randomized designs were excluded.

This deliberate homogenization across all PICOS dimensions was designed to minimize clinical and methodological heterogeneity, thereby supporting the assumption that the relative treatment effects estimated from different pairwise comparisons within the network can be validly compared (transitivity). In addition, the network topology in Section 4 exhibited a star-shaped configuration without closed-loop connections between TCM interventions, resulting in exclusively indirect comparisons. Given this specific topological property, which precludes the possibility of direct comparisons forming loops, the assumption of consistency is structurally satisfied within our network.

### 2.2 Search strategy

This study employed a multi-database differentiated search strategy to systematically retrieve RCTs on TCMs for hyperlipidemia or dyslipidemia. Comprehensive searches were conducted across PubMed, Web of Science, Cochrane Library, and four Chinese databases (CNKI, Wanfang, VIP, and CBM databases), covering records from each database’s inception to September 30, 2024. The search strategy was constructed based on three core domains: disease (hyperlipidemia, dyslipidemia), interventions (Xuezhikang, Dantian Jiangzhi, Jiangzhiling, Pushen, Songling Xuemaikang, Jiangzhi Tongluo, Jiangzhi Tongmai, Xuezhitong), and study design (randomized, clinical). English databases utilized a combination of title/abstract/keyword field restrictions and free-text terms. Chinese databases adopted fuzzy field matching and intelligent word segmentation to build compound queries. Boolean operators (AND/OR) were systematically applied to combine terms across domains, strictly adhering to each database’s syntax rules. Detailed search strategies for all databases were fully documented in Supplementary Material S4 to ensure methodological transparency and reproducibility.

### 2.3 Literature screening and data extraction

All identified literature from various databases was initially de-duplicated through NoteExpress. Following de-duplication, the literature was screened by reviewing titles, abstracts, and keywords. Subsequently, a full-text review was performed to further refine the selection according to the predetermined inclusion and exclusion criteria, excluding any literature that did not fulfill the requirements. Data extraction was carried out autonomously by two researchers (Ling Jiang and Maoxing Pan) with the background in evidence-based medicine. In the event of any discrepancies during this process, a senior researcher (Yupei Zhang) was involved to assist the judgement. The data extracted included information such as the literature author, publication year, number of cases, treatment duration, intervention measures, outcomes, and adverse reactions.

### 2.4 Quality assessment and evidence evaluation

Two researchers conducted independently the quality assessment of identified studies (Ling Jiang and Maoxing Pan), using the bias risk assessment tool recommended by the Cochrane tool RoB 2.0 (Higgins et al., 2011). The assessment covered 5 key domains: randomization process, deviations from intended interventions, missing outcome data, measurement of the outcome, and selection of the reported results. Each domain was evaluated and categorized as high risk, low risk, or some concerns. When disagreements arose, a third researcher was sought to facilitate a consensus.

The GRADE system is a sophisticated tool employed in NMA to assess the evidence quality from the underlying studies, encompassing study design, risk of bias, consistency, directness, precision, and the possibility of publication bias (Balshem et al., 2011). The culmination of these assessments leads to the classification of evidence certainty into four tiers: high, moderate, low, and very low.

### 2.5 Statistical analysis

#### 2.5.1 Data synthesis analysis

This study employed R, STATA and RevMan software for data processing and statistical analysis. Risk of bias graph for included RCTs was plotted using R. The direct meta-analysis was conducted for each intervention by RevMan. The random-effect model would be used if high data heterogeneity (*I* ^2^ ≥ 50% or *P* < 0.05) was detected, otherwise, the fixed-effect model would be applied with low data heterogeneity (*I* ^2^ < 50% or *P* ≥ 0.05). For dichotomous outcome, clinical total effective rate, the odds ratio (OR) served as the effect size, whereas for continuous outcomes such as TC, TG, HDL-C, and LDL-C, the mean difference (MD) was the chosen effect size. In each case, a 95% confidence interval (CI) was computed for the respective effect size. If the 95% CI of the OR included 1 or the 95% CI of the MD included 0, the intervention was considered statistically non-significant.

#### 2.5.2 Network meta-analysis

Utilizing the R with a Bayesian NMA model (Mills et al., 2013) and calling the gemtc package, 4 Markov chains were set, with 5000 pre-iterations and 20,000 iteration numbers, a step length of 1, observing the trace density plot to assess the model fit. The prior distribution for the random-effects variance (*τ* ^2^) was specified as a uniform distribution: *τ* ^2^ ∼ Uniform (0, 0.5). If the Potential Scale Reduction Factor (PSRF) approaches convergence at close to 1.00 (PSRF <1.05), the model is well-converged, and the NMA is performed. The network evidence plot was drawn by R. Each node in the network symbolized a unique intervention, and the robustness of the connections between them was indicative of the volume of direct comparative RCTs. For each outcome, the league table would be obtained to compare the effect size of different interventions, and the Surface Under the Cumulative Ranking curve (SUCRA) was calculated to rank the total efficacy of interventions (Salanti et al., 2011).

#### 2.5.3 Heterogeneity, sensitivity and publication bias analysis

The meta-regression approach (Thompson and Higgins, 2002) and subgroup analysis (Fingerhut et al., 2024) were employed to explore the heterogeneity sources by STATA when *I* ^2^ > 50%, including sample size, treatment duration, patients average age, whether or not combined other diseases, and different TT. To assess the robustness of results, sensitivity analysis (Thabane et al., 2013) was conducted. Publication bias analysis was evaluated with begg’s and egger’s test (Begg and Mazumdar, 1994; Egger et al., 1997), as well as trim and fill method.

## 3 Results

### 3.1 Literature search results

**FIGURE 1:**
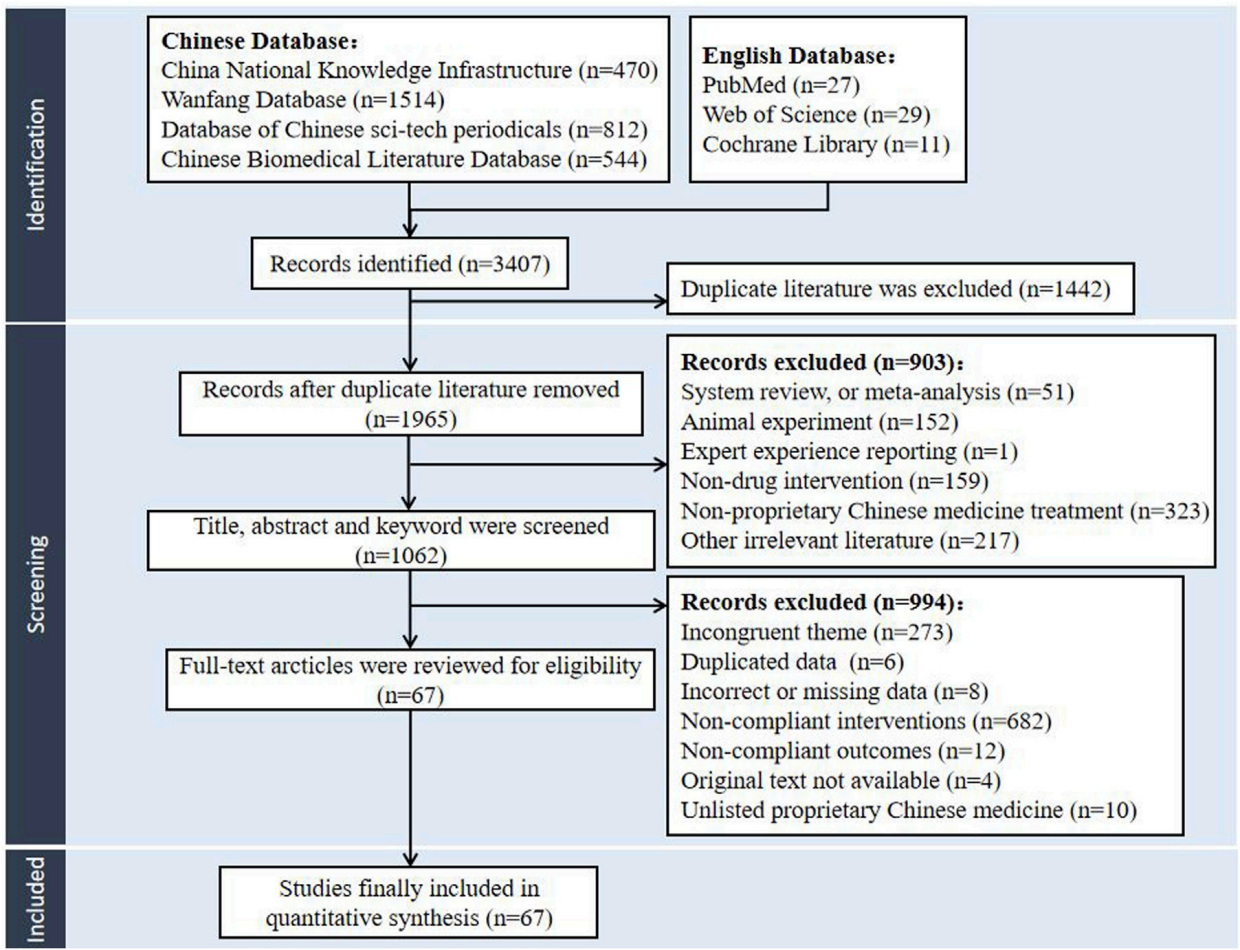
Literature screening protocol.

The literature search recognized 3407 potentially relevant papers from the databases of CNKI, VIP, CBM, Wanfang database, Web of Science, PubMed, and the Cochrane library. After the de-duplication process facilitated by NoteExpress, the collection was refined to 1965 articles, with 1915 articles written in Chinese and 50 in English. Adhering to the predefined criteria for inclusion and exclusion, the initial screening phase involved a meticulous review of titles, abstracts, and keywords, culminating in the selection of 1062 articles for further evaluation. Subsequently, comprehensive full-text analysis led to the selection of 67 studies. Finally, 67 articles were incorporated into the NMA, all of which were in Chinese. The literature screening protocol was delineated in Figure 1.

### 3.2 Basic characteristics of included studies

The total of 67 studies were incorporated into the analysis, all characterized as two-arm RCTs, amassing a collective participant count of 7327 individuals. A total of 3681 patients were enrolled in the treatment group, and 3646 patients were part of the control group. These studies evaluated the spectrum of 8 TCMs and 9 intervention strategies, encompassing conventional TT, in conjunction with various TCMs including TT + DTJZ (5 RCTs), TT + JZL (4 RCTs), TT + JZTL (4 RCTs), TT + JZTM (5 RCTs), TT + PS (10 RCTs), TT + SLXMK (6 RCTs), TT + XZK (27 RCTs), and TT + XZT (6 RCTs). The fundamental detailed attributes of the included studies were provided in Supplementary Material S5.

### 3.3 Risk of bias assessment of included studies

**FIGURE 2:**
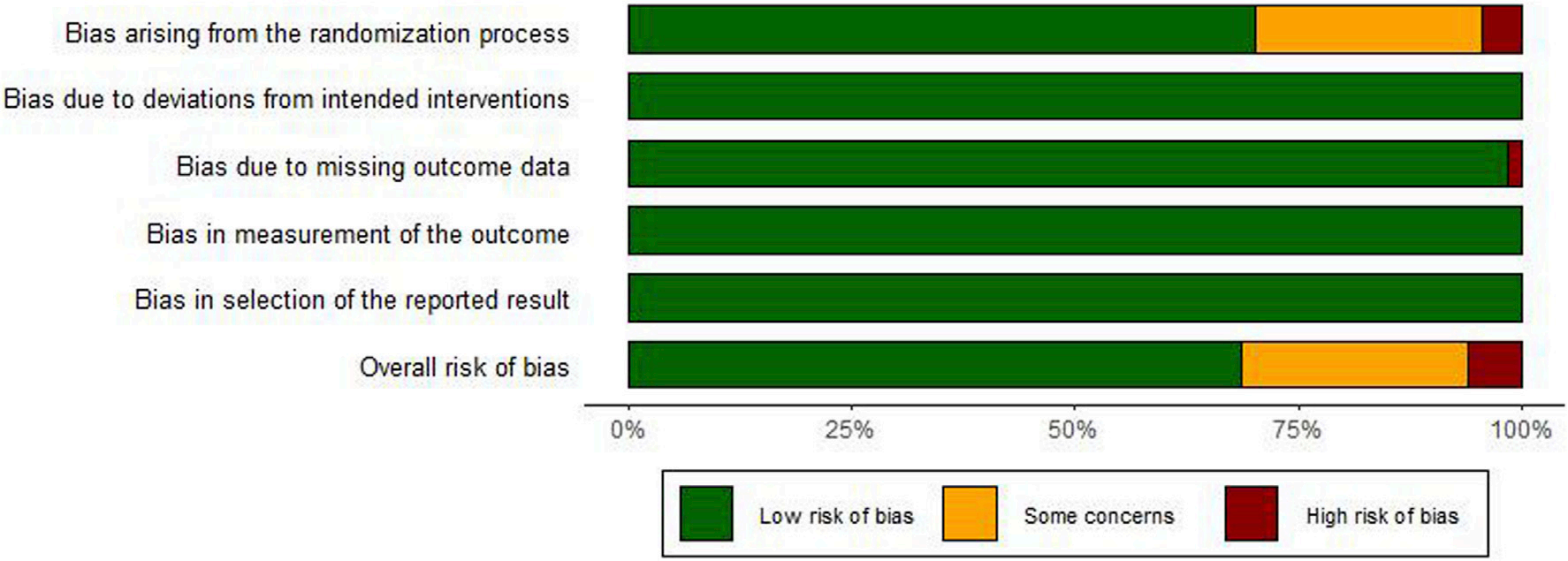
Risk assessment of bias for include studies.

Of the 67 enrolled RCTs, 47 studies utilized the random number table method or stated that the group assignment was not statistically significant for randomization and were classified as “low risk”, whereas 3 studies (Xiao, 2014; Li, 2016; Feng, 2013) employed a randomization strategy based on the sequence of clinical visits, which was classified as “high risk”. The remaining 17 studies failed to specify the randomization technique, leading to a classification of “unclear risk”. Besides, all studies reported no bias due to deviations from intended interventions and employed appropriate method to study the intervention effects, leading to the “low risk”. With respect to data integrity, one study (Xie et al., 2014) reported participant withdrawal, leading to the “high risk” classification. All other studies showed complete data, and were therefore classified as “low risk”. Moreover, the measurement of the outcomes in all studies was consistent and objective, thus leading to the “low risk”. No evidence of selective reporting was identified across the studies, meriting the “low risk” classification. The evaluation of bias risk within the included studies was depicted in Figure 2 and the detailed traffic diagram was depicted in Supplementary Material S6. Among the included RCTs, 69% demonstrated low risk of bias, 6% were rated as high risk, and the remaining 25% raised some concerns regarding potential bias.

## 4 Meta-analysis

### 4.1 Primary outcome

**FIGURE 3:**
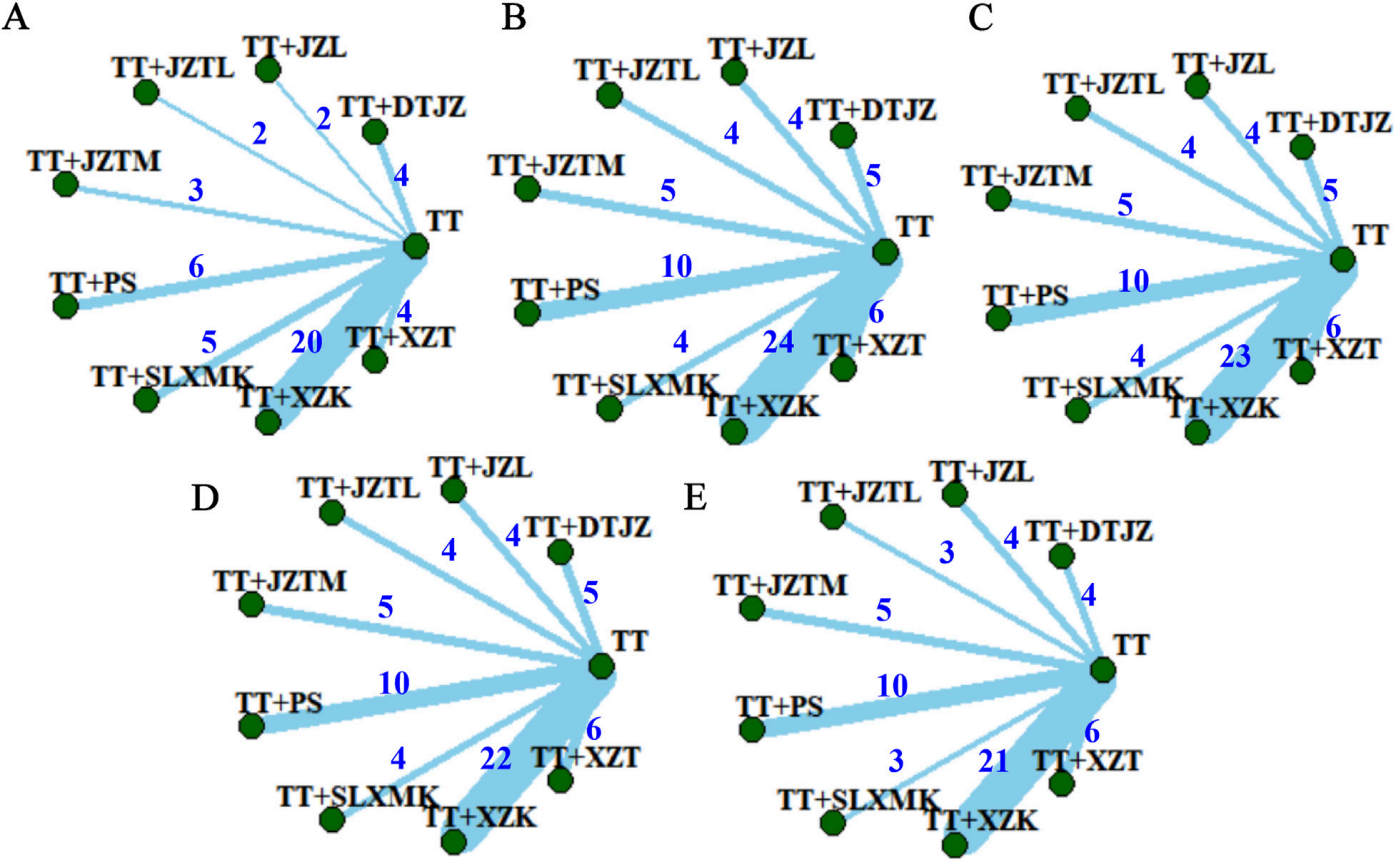
Network evidence plots of **(A)** clinical total effective rate, **(B)** TC, **(C)** TG, **(D)** HDL-C and **(E)** LDL-C.

The clinical total effective rate was delineated in 46 studies. The interventions pertained to 8 TCMs, encompassing 9 intervention strategies, and involved a cohort of 5479 participants. A nine-node network relationship had been constructed, positioning TT-based lipid-lowering medications at its nucleus, as shown in Figure 3A. Notably, the most extensive research had been dedicated to the comparative efficacy of the combination therapy of TT + XZK versus TT monotherapy, with a total of 20 studies contributing to this comparison.

#### 4.1.1 Direct meta-analysis of clinical total effective rate

The direct meta-analysis of clinical total effective rate included 8 pairs of comparison, utilizing OR as the effect size. The forest plot was shown in Supplementary Material S7.1. It presented the comparative evaluation of the efficacy between the combination therapy of TCMs with TT and TT monotherapy. The findings revealed that the clinical total effective rate for the combination of TT and all TCMs consistently outperformed TT monotherapy, with significant statistical disparities (*P* < 0.05). Among them, the interventions of TT + SLXMK resulted in considerable heterogeneity (*I* ^2^ = 68%, *P* = 0.01).

#### 4.1.2 Heterogeneity analysis of clinical total effective rate

Due to the limited number of studies on TT + SLXMK, the reliability of meta-regression analysis was compromised. Subgroup analysis was performed to identify possible causes of heterogeneity, including sample size, treatment duration, average age, and whether or not combined with other diseases, as depicted in Supplementary Material S8.1. The results indicated that the heterogeneity may related to the difference in whether or not combined with other diseases, as shown in Supplementary Material S8.1.1. A statistical difference was observed between the two subgroups (*P* = 0.004) and the heterogeneity of each subgroup was lowered. Therefore, for the clinical total effective rate, there was no significant heterogeneity across all intervention measures.

#### 4.1.3 Network meta-analysis of clinical total effective rate

Given the absence of closed loops among the various TCM interventions, direct comparisons were not present. All pairwise contrasts were the result of indirect comparisons. Consequently, the analysis was conducted using the consistency model. The PSRF, nearing 1.00, signified an excellent level of research convergence and lent strong credibility to the analysis outcomes. The NMA had yielded a total of 36 pairwise comparisons, with the OR for 8 of these comparisons exhibiting 95% CI that did not encompass 1, indicating statistically significant differences. The findings suggested that, in comparison to TT monotherapy, the combination of DTJZ, JZL, JZTL, JZTM, PS, SLXMK, XZK, and XZT with TT therapy could considerably enhance the clinical total effective rate, with the following OR and 95% CI values: [OR = 3.11, 95% CI (1.84, 5.39)], [OR = 5.6, 95% CI (1.63, 26.97)], [OR = 4.38, 95% CI (1.91, 11.12)], [OR = 5.79, 95% CI (2.5, 15.23)], [OR = 3.53, 95% CI (2.09, 6.17)], [OR = 3.38, 95% CI (2.28, 5.14)], [OR = 4.02, 95% CI (3.08, 5.3)], and [OR = 3.69, 95% CI (1.87, 7.74)], respectively. The remaining interventions showed no statistically significant differences in pairwise comparisons, as detailed in Table 1.

**TABLE 1:** League table of clinical total effective rate.

**TT + DTJZ**								
0.55 (0.11,2.13)	**TT + JZL**							
0.71 (0.24,1.9)	1.28 (0.27,7.57)	**TT + JZTL**						
0.54 (0.18,1.46)	0.97 (0.2,5.68)	0.75 (0.21,2.63)	**TT + JZTM**					
0.88 (0.41,1.89)	1.59 (0.41,8.31)	1.24 (0.46,3.6)	1.64 (0.6,4.91)	**TT + PS**				
0.92 (0.47,1.81)	1.66 (0.45,8.31)	1.3 (0.52,3.54)	1.71 (0.67,4.89)	1.04 (0.53,2.06)	**TT + SLXMK**			
0.77 (0.43,1.43)	1.39 (0.39,6.89)	1.09 (0.46,2.86)	1.44 (0.6,3.93)	0.88 (0.48,1.63)	0.84 (0.52,1.38)	**TT + XZK**		
0.84 (0.34,2.01)	1.53 (0.36,8.35)	1.19 (0.39,3.7)	1.57 (0.51,5.08)	0.95 (0.39,2.31)	0.92 (0.4,2.03)	1.09 (0.5,2.26)	**TT + XZT**	
**3.11 (1.84,5.39)**	**5.6 (1.63,26.97)**	**4.38 (1.91,11.12)**	**5.79 (2.5,15.23)**	**3.53 (2.09,6.17)**	**3.38 (2.28,5.14)**	**4.02 (3.08,5.3)**	**3.69 (1.87,7.74)**	**TT**

The OR and 95% CI marked in bold indicate statistically significant pairwise comparisons.

**FIGURE 4:**
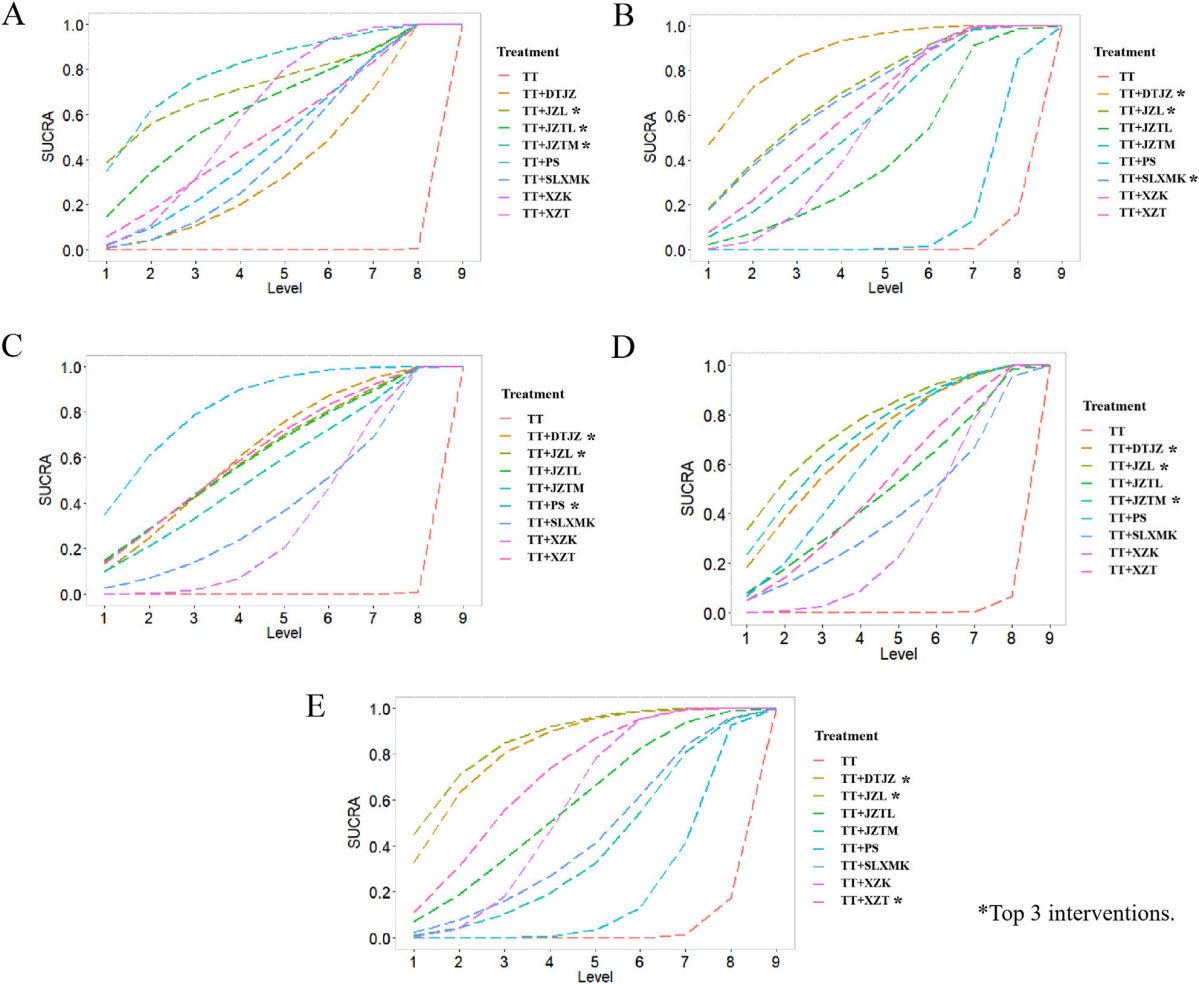
SUCRA rank of **(A)** clinical total effective rate, **(B)** TC, **(C)** TG, **(D)** HDL-C and **(E)** LDL-C.

According to the rankings derived from SUCRA curve (Figure 4A), the hierarchy of interventions, based on clinical total effective rate, was as follows: the combination of TT with JZTM secured the top spot with a SUCRA value of 79.2%, followed closely by the combination with JZL at 72.5%. The JZTL in conjunction with TT claimed the third position with a SUCRA value of 62.8%. TT paired with XZK hold a SUCRA value of 59.5%, while the combination with XZT was credited with 50.9%. The integration of TT with PS achieved a SUCRA value of 46.8%, and the pairing with SLXMK received a SUCRA value of 42.1%. The combination of TT and DTJZ was noted with a SUCRA value of 36.1%. Lastly, TT monotherapy was at the base of the rankings with a SUCRA value of 0.0%.

### 4.2 Secondary outcomes

#### 4.2.1 TC

TC was delineated in 62 studies. The interventions pertained to 8 TCMs, encompassing 9 intervention strategies, and involved a cohort of 6347 participants. A nine-node network relationship had been constructed in Figure 3B. The most extensive research had been dedicated to the comparative efficacy of the combination therapy of TT and XZK versus TT monotherapy, with a total of 24 studies contributing to this comparison.

##### 4.2.1.1 Direct meta-analysis of TC

The direct meta-analysis of TC included 8 pairs of comparisons, utilizing MD as the effect size. The forest plot was shown in Supplementary Material S7.2. It presented the comparative evaluation of the efficacy between the combination therapy of TCMs with TT and TT monotherapy. The findings revealed that the TC for the combination of TT and DTJZ, JZL, JZTL, JZTM, PS, SLXMK, XZK, and XZT consistently outperformed TT monotherapy, with significant statistical disparities (*P* < 0.05). Among them, the interventions of TT + DTJZ, TT + JZTL, TT + JZTM, TT + SLXMK, TT + XZK and TT + XZT resulted in considerable heterogeneity.

##### 4.2.1.2 Meta-regression and subgroup analysis of TC

For the interventions of TT + XZK, meta-regression approach was performed to identify possible causes of heterogeneity. The results indicated that the high heterogeneity was concerned with sample size (*P* = 0.034), and not related to treatment duration (*P* = 0.44), average age (*P* = 0.846), whether or not combined with other diseases (*P* = 0.993) and different TT (*P* = 0.280). As shown in Supplementary Material S8.2.1, subgroup analysis indicated that there existed statistical significance between the two subgroups based on sample size (*P* = 0.003). Besides, subgroup analysis showed statistical significance among subgroups with different TTs (*P* = 0.002), suggesting that varying TT treatments might be a source of heterogeneity in TT + XZK.

As for the interventions of TT + DTJZ, TT + JZTL, TT + JZTM, TT + SLXMK, and TT + XZT, no enough studies were involved to adopt meta regression method. Subgroup analysis was conducted to identify possible causes of heterogeneity, including sample size, treatment duration, average age, whether or not combined with other diseases, and different TT, as depicted in Supplementary Material S8.2. The results indicated that, for TT + XZT, the heterogeneity may be related to the difference in sample size, as shown in Supplementary Material S8.2.3. There exists statistical significance between the two subgroups (*P* = 0.002) and the heterogeneity of each subgroup was lowered. Subgroup analysis of other interventions showed no significant reduction in heterogeneity and no statistical significance between subgroups.

Therefore, significant heterogeneity remained for the interventions TT + DTJZ, TT + JZTL, TT + JZTM, TT + SLXMK, and TT + XZK after subgroup analysis (*I* ^2^ = 91%, 58%, 67%, 60%, and 88%, respectively). The heterogeneity may stem from variations in statin types used, whether patients continued their original lipid-lowering medications, total sample size, mean age, and treatment duration. Specifically, in studies involving TT + DTJZ, the sample size in Lv WH’s work (n = 316) (Lv, 2013) was significantly larger than in other studies. In studies involving TT + JZTL, the treatment duration in Xie Y’s study (8 weeks) (Xie et al., 2014) was shorter than in other studies (≥12 weeks), potentially leading to an underestimation of the intervention group’s effect size. In studies involving TT + JZTM, the TCM medication dosage in Liu FG’s study (3 g/day) (Liu and Chen, 2011) was lower than in other studies, possibly underestimating the intervention’s effectiveness. For TT + SLXMK, only Li HL’s study (Li et al., 2011) used fluvastatin as the control group medication.

##### 4.2.1.3 Network meta-analysis of TC

The NMA had yielded a total of 36 pairwise comparisons, with the MD for 13 of these comparisons exhibiting 95% CI that did not encompass 0, indicating statistically significant differences. The findings suggested that, in comparison to TT monotherapy, the combination of DTJZ, JZL, JZTL, JZTM, SLXMK, XZK, and XZT with TT therapy can significantly enhance TC, with the following MD and 95% CI values: [MD = −0.95, 95% CI (−1.31, −0.59)], [MD = −0.78, 95% CI (−1.22, −0.34)], [MD = −0.51, 95% CI (−0.96, −0.06)], [MD = −0.66, 95% CI (−1.04, −0.28)], [MD = −0.78, 95% CI (−1.24, −0.31)], [MD = −0.6, 95% CI (−0.77, −0.43)] and [MD = −0.7, 95% CI (−1.08, −0.33)], respectively. The therapeutic effects of DTJZ, JZL, JZTM, SLXMK, XZK, and XZT when combined with TT were superior to the combination of PS with TT, with statistically significant differences, respectively represented as [MD = −0.8, 95% CI (−1.25,-0.35)], [MD = −0.63, 95% CI (−1.15,-0.11)], [MD = −0.52, 95% CI (−0.98,-0.04)], [MD = −0.63, 95% CI (−1.16,-0.09)], [MD = −0.46, 95% CI (−0.77,-0.16)], and [MD = −0.56, 95% CI (−1.02,-0.1)]. The remaining interventions showed no statistically significant differences in pairwise comparisons, as detailed in Table 2, the lower left triangular red region.

**TABLE 2:** League table of TC (lower left triangular red region) and TG (upper right triangular green region).

TT + DTJZ	0 (−0.38,0.38)	0.01 (−0.38,0.39)	0.04 (−0.35,0.42)	−0.1 (−0.42,0.21)	0.12 (−0.24,0.49)	0.14 (−0.12,0.4)	0 (−0.36,0.36)	0.49 (0.25,0.72)
−0.17 (−0.74,0.41)	TT + JZL	0 (−0.42,0.43)	0.04 (−0.39,0.46)	−0.11 (−0.47,0.26)	0.12 (−0.29,0.53)	0.14 (−0.18,0.46)	0 (−0.41,0.41)	**0.49 (0.19,0.78)**
−0.44 (−1.01,0.14)	−0.27 (−0.89,0.36)	TT + JZTL	0.03 (−0.4,0.47)	−0.11 (−0.48,0.26)	0.12 (−0.3,0.53)	0.14 (−0.19,0.46)	0 (−0.42,0.41)	**0.48 (0.17,0.79)**
−0.29 (−0.81,0.23)	−0.12 (−0.71,0.46)	0.15 (−0.44,0.73)	TT + JZTM	−0.14 (−0.5,0.23)	0.08 (−0.32,0.5)	0.1 (−0.22,0.43)	−0.04 (−0.44,0.38)	**0.45 (0.15,0.76)**
**−0.8 (-1.25,-0.35)**	**−0.63 (-1.15,-0.11)**	−0.36 (−0.89,0.16)	**−0.52 (-0.98,-0.04)**	TT + PS	0.23 (−0.12,0.57)	**0.24 (0.01,0.48)**	0.11 (−0.24,0.45)	**0.59 (0.39,0.79)**
−0.17 (−0.76,0.41)	0 (−0.64,0.63)	0.27 (−0.38,0.9)	0.12 (−0.48,0.71)	**0.63 (0.09,1.16)**	TT + SLXMK	0.02 (−0.28,0.32)	−0.12 (−0.52,0.27)	**0.37 (0.08,0.65)**
−0.35 (−0.75,0.05)	−0.18 (−0.65,0.3)	0.09 (−0.39,0.57)	−0.06 (−0.47,0.36)	**0.46 (0.13,0.77)**	−0.18 (−0.67,0.32)	TT + XZK	−0.14 (−0.44,0.16)	**0.35 (0.24,0.45)**
−0.25 (−0.76,0.27)	−0.08 (−0.65,0.5)	0.19 (−0.39,0.77)	0.04 (−0.49,0.58)	**0.56 (0.1,1.02)**	−0.08 (−0.66,0.52)	0.1 (−0.31,0.51)	TT + XZT	**0.48 (0.21,0.76)**
**−0.95 (-1.31,-0.59)**	**−0.78 (-1.22,-0.34)**	**−0.51 (-0.96,-0.06)**	**−0.66 (-1.04,-0.28)**	−0.15 (−0.42,0.12)	**−0.78 (-1.24,-0.31)**	**−0.6 (-0.77,-0.43)**	**−0.7 (-1.08,-0.33)**	TT

The OR and 95% CI marked in bold indicate statistically significant pairwise comparisons.

According to the rankings derived from SUCRA curve (Figure 4B), the hierarchy of interventions, based on TC, was as follows: the combination of TT with DTJZ secured the top spot with a SUCRA value of 87.7%, followed closely by the combination with JZL at 70.1%. The SLXMK in conjunction with TT claimed the third position with a SUCRA value of 69.8%. TT paired with XZT hold a SUCRA value of 61.9%, while the combination with JZTM was credited with 57.0%. The integration of TT with XZK achieved a SUCRA value of 48.1%, and the pairing with JZTL received a SUCRA value of 41.1%. The combination of TT and PS was noted with a SUCRA value of 12.6%. Lastly, TT monotherapy was at the base of the rankings with a SUCRA value of 2.0%.

#### 4.2.2 TG

TG was delineated in 61 studies. The interventions pertained to 8 TCMs, encompassing 9 intervention strategies, and involved a cohort of 6269 participants. A nine-node network relationship had been constructed in Figure 3C. The most extensive research had been dedicated to the comparative efficacy of the combination therapy of TT and XZK versus TT monotherapy, with a total of 23 studies contributing to this comparison.

##### 4.2.2.1 Direct meta-analysis of TG

The direct meta-analysis of TG included 8 pairs of comparisons, utilizing MD as the effect size. The forest plot was shown in Supplementary Material S7.3. It presented the comparative evaluation of the efficacy between the combination therapy of TCMs with TT and TT monotherapy. The findings revealed that the TG for the combination of TT and all 8 TCMs consistently outperformed TT monotherapy, with significant statistical disparities (*P* < 0.05). Among them, the interventions of TT + DTJZ, TT + JZL, TT + JZTM, TT + PS, TT + SLXMK, and TT + XZK resulted in considerable heterogeneity.

##### 4.2.2.2 Meta-regression and subgroup analysis of TG

For the interventions of TT + PS and TT + XZK, meta-regression approach was performed to identify possible causes of heterogeneity. The results indicated that there was no statistical significance between the different subgroups based on sample size (*P* = 0.15 and 0.563), treatment duration (*P* = 0.19 and 0.732), average age (*P* = 0.33 and 0.806), whether or not combined with other diseases (*P* = 0.096 and 0.97), and different TT (*P* = 0.4 and 0.1). However, the subgroup analysis of TT + PS based on whether or not combined with other diseases showed that, the corresponding *P*-value of different subgroups was 0.04, as shown in Supplementary Material S8.3, 8.3.1. It might be the possible source of heterogeneity in TT + PS.

As for the interventions of TT + DTJZ, TT + JZL, TT + JZTM, and TT + SLXMK, no enough studies were involved to adopt meta regression method. Subgroup analysis was conducted to identify possible causes of heterogeneity, as depicted in Supplementary Material S8.3. The results indicated that, for TT + DTJZ, the heterogeneity may be relevant to the difference in sample size and whether or not combined with other diseases, as shown in Supplementary Material S8.3.2, 8.3.3. There exists statistical significance between the two subgroups (*P <* 0.00001) and the heterogeneity of each subgroup was lowered. For TT + JZL, the heterogeneity may be related to treatment duration and average age, and the corresponding *P*-value of different subgroups were both 0.04, as shown in Supplementary Material S8.3.4, 8.3.5. For TT + JZTM, the heterogeneity may be concerned with sample size and different TT. The corresponding *P*-value of different subgroups were 0.001 and 0.0007, respectively, as shown in Supplementary Material S8.3.6, 8.3.7. Subgroup analysis of other interventions showed no significant reduction in heterogeneity and no statistical significance between subgroups.

Therefore, significant heterogeneity remained for the interventions TT + PS, TT + SLXMK, and TT + XZK after subgroup analysis (I^2^ = 64%, 72%, and 91%, respectively). The heterogeneity may arise from differences in treatment duration, statin types used, and medication dosages. Notably, in studies involving TT + SLXMK, the treatment duration in Ning Y’s study (Ning and Ding, 2012) was only 4 weeks, which may lead to unstable intervention outcomes.

##### 4.2.2.3 Network meta-analysis of TG

The NMA had yielded a total of 36 pairwise comparisons, with the MD for 9 of these comparisons exhibiting 95% CI that did not encompass 0, indicating statistically significant differences. The findings suggested that, in comparison to TT monotherapy, the combination of all 8 TCMs with TT therapy can significantly enhance TG, with the following MD and 95% CI values: [MD = −0.49, 95% CI (−0.72, −0.25)], [MD = −0.49, 95% CI (−0.78, −0.19)], [MD = −0.48, 95% CI (−0.79, −0.17)], [MD = −0.45, 95% CI (−0.76, −0.15)], [MD = −0.59, 95% CI (−0.79, −0.39)], [MD = −0.37, 95% CI (−0.65, −0.08)], [MD = −0.35, 95% CI (−0.45, −0.24)] and [MD = −0.48, 95% CI (−0.76, −0.21)], respectively. The therapeutic effects of PS when combined with TT were superior to the combination of XZK with TT, with statistically significant differences, respectively represented as [MD = −0.24, 95% CI (−0.48, −0.01)]. The remaining interventions showed no statistically significant differences in pairwise comparisons, as detailed in Table 2, the upper right triangular green region.

According to the rankings derived from SUCRA curve (Figure 4C), the hierarchy of interventions, based on TG, was as follows: the combination of TT with PS secured the top spot with a SUCRA value of 82.2%, followed closely by the combination with DTJZ at 62.1%. The JZL in conjunction with TT claimed the third position with a SUCRA value of 61.1%. TT paired with XZT hold a SUCRA value of 61.0%, while the combination with JZTL was credited with 60.2%. The integration of TT with JZTM achieved a SUCRA value of 54.0%, and the pairing with SLXMK received a SUCRA value of 38.5%. The combination of TT and XZK was noted with a SUCRA value of 30.7%. Lastly, TT monotherapy was at the base of the rankings with a SUCRA value of 0.1%.

#### 4.2.3 HDL-C

HDL-C was delineated in 60 studies. The interventions pertained to 8 TCMs, encompassing 9 intervention strategies, and involved a cohort of 6220 participants. A nine-node network relationship had been constructed in Figure 3D. The most extensive research had been dedicated to the comparative efficacy of the combination therapy of TT and XZK versus TT monotherapy, with a total of 22 studies contributing to this comparison.

##### 4.2.3.1 Direct meta-analysis of HDL-C

The direct meta-analysis of HDL-C encompassed 8 pairs of comparisons, utilizing MD as the effect size. The forest plot was shown in Supplementary Material S7.4. It presented a comparative evaluation of the efficacy between the combination therapy of TCMs with TT and TT monotherapy. The findings revealed that the HDL-C for the combination of TT and all 8 TCMs consistently outperformed TT monotherapy, with significant statistical disparities (*P* < 0.05). All the interventions resulted in considerable heterogeneity.

##### 4.2.3.2 Meta-regression and subgroup analysis of HDL-C

For the interventions of TT + PS and TT + XZK, meta-regression approach was performed to identify possible causes of heterogeneity. The results indicated that, for TT + XZK, the high heterogeneity was concerned with average age (*P* = 0.003), and not related to sample size (*P* = 0.921), treatment duration (*P* = 0.468), whether or not combined with other diseases (*P* = 0.984), and different TT (*P* = 0.669). As shown in Supplementary Material S8.4.4, subgroup analysis indicated that there was statistical significance between the two subgroups based on average age (*P <* 0.00001). For TT + PS, there was no statistical significance between the different subgroups.

As for the interventions of TT + DTJZ, TT + JZL, TT + JZTL, TT + JZTM, TT + SLXMK, and TT + XZT, no enough studies were involved to adopt meta regression method. Subgroup analysis was performed to identify possible causes of heterogeneity, as depicted in Supplementary Material S8.4. The results indicated that, for TT + DTJZ, the heterogeneity may be relevant to the difference in sample size and whether or not combined with other diseases, as shown in Supplementary Material S8.4.1, 8.4.2. There exists statistical significance between the two subgroups (*P =* 0.002) and the heterogeneity of each subgroup was lowered. For TT + JZTM, the heterogeneity may be relevant to sample size and different TT. The corresponding *P*-value of different subgroups were 0.01 and 0.00001, respectively, as shown in Supplementary Material S8.4.3, 8.4.4. Subgroup analysis of other interventions showed no significant reduction in heterogeneity and no statistical significance between subgroups.

Therefore, significant heterogeneity remained for the interventions TT + DTJZ, TT + JZL, TT + JZTL, TT + JZTM, TT + PS, TT + SLXMK, TT + XZK, and TT + XZT after subgroup analysis (*I* ^2^ = 92%, 84%, 83%, 96%, 97%, 51%, 79%, and 86%, respectively). The heterogeneity likely originates from variations in statin types used, whether patients continued their original lipid-lowering medications, total sample size, mean age, and treatment duration. Specifically, in studies involving TT + JZL, the maximum TCM medication dose in Zhang XL’s study (Zhang et al., 2017) reached 16 g/day, potentially leading to an overestimation of the intervention group’s effect size. For TT + JZTL, the shorter treatment duration (8 weeks) in Xie Y’s study (Xie et al., 2014) compared to other studies (≥12 weeks) may cause an underestimation of the effect size. In studies involving TT + JZTM, the 6 g/day dosage in the treatment group in Wang H’s study (Wang, 2019) may lead to an overestimation of the effect size. Regarding TT + SLXMK, only Li HL’s study (Li et al., 2011) used fluvastatin as the control group medication, which may cause discrepancies in efficacy outcomes.

##### 4.2.3.3 Network meta-analysis of HDL-C

The NMA had yielded a total of 36 pairwise comparisons, with the MD for 7 of these comparisons exhibiting 95% CI that did not encompass 0, indicating statistically significant differences. The findings suggested that, in comparison to TT monotherapy, the combination of DTJZ, JZL, JZTL, JZTM, PS, XZK, and XZT with TT therapy can significantly enhance HDL-C, with the following MD and 95% CI values: [MD = 0.34, 95% CI (0.13, 0.55)], [MD = 0.38, 95% CI (0.14, 0.61)], [MD = 0.26, 95% CI (0.02, 0.5)], [MD = 0.35, 95% CI (0.14, 0.57)], [MD = 0.31, 95% CI (0.16, 0.45)], [MD = 0.21, 95% CI (0.11, 0.31)] and [MD = 0.27, 95% CI (0.09, 0.46)], respectively. The remaining interventions showed no statistically significant differences in pairwise comparisons, as detailed in Table 3, the lower left triangular red region.

**TABLE 3:** League table of HDL-C (lower left triangular red region) and LDL-C (upper right triangular green region).

TT + DTJZ	−0.04 (−0.47,0.4)	0.19 (−0.25,0.64)	0.33 (−0.11,0.76)	0.47 (0.12,0.82)	0.3 (−0.15,0.74)	0.23 (−0.08,0.55)	0.11 (−0.29,0.51)	0.61 (0.32,0.9)
−0.04 (−0.35,0.28)	TT + JZL	0.23 (−0.23,0.69)	0.36 (−0.08,0.81)	**0.51 (0.13,0.87)**	0.33 (−0.13,0.8)	0.27 (−0.07,0.61)	0.14 (−0.27,0.56)	**0.65 (0.33,0.96)**
0.08 (−0.24,0.4)	0.11 (−0.22,0.46)	TT + JZTL	0.14 (−0.33,0.6)	0.28 (−0.11,0.67)	0.11 (−0.38,0.58)	0.04 (−0.32,0.4)	−0.08 (−0.52,0.35)	0.42 (0.08,0.75)
−0.01 (−0.31,0.28)	0.02 (−0.3,0.34)	−0.09 (−0.42,0.23)	TT + JZTM	0.14 (−0.24,0.52)	−0.03 (−0.5,0.44)	−0.1 (−0.44,0.25)	−0.22 (−0.63,0.2)	0.28 (−0.04,0.6)
0.03 (−0.22,0.29)	0.07 (−0.21,0.35)	−0.04 (−0.33,0.24)	0.04 (−0.21,0.31)	TT + PS	−0.17 (−0.57,0.22)	**−0.24 (-0.47,0)**	**−0.36 (-0.69,-0.03)**	0.14 (−0.05,0.33)
0.12 (−0.21,0.45)	0.16 (−0.19,0.51)	0.04 (−0.31,0.4)	0.13 (−0.2,0.47)	0.09 (−0.21,0.39)	TT + SLXMK	−0.06 (−0.43,0.3)	−0.19 (−0.62,0.25)	0.31 (−0.03,0.66)
0.13 (−0.1,0.36)	0.17 (−0.09,0.42)	0.05 (−0.21,0.31)	0.14 (−0.09,0.38)	0.09 (−0.08,0.27)	0.01 (−0.27,0.28)	TT + XZK	−0.12 (−0.42,0.18)	**0.38 (0.25,0.5)**
0.07 (−0.21,0.35)	0.1 (−0.19,0.41)	−0.01 (−0.31,0.3)	0.08 (−0.2,0.37)	0.03 (−0.2,0.27)	−0.05 (−0.37,0.27)	−0.06 (−0.27,0.15)	TT + XZT	**0.5 (0.23,0.77)**
**0.34 (0.13,0.55)**	**0.38 (0.14,0.61)**	**0.26 (0.02,0.5)**	**0.35 (0.14,0.57)**	**0.31 (0.16,0.45)**	0.22 (−0.04,0.48)	**0.21 (0.11,0.31)**	**0.27 (0.09,0.46)**	TT

The OR and 95% CI marked in bold indicate statistically significant pairwise comparisons.

According to the rankings derived from SUCRA curve (Figure 4D), the hierarchy of interventions, based on HDL-C, was as follows: the combination of TT with JZL secured the top spot with a SUCRA value of 76.0%, followed closely by the combination with JZTM at 71.3%. The DTJZ in conjunction with TT claimed the third position with a SUCRA value of 68.2%. TT paired with PS hold a SUCRA value of 61.1%, while the combination with XZT was credited with 51.2%. The integration of TT with JZTL achieved a SUCRA value of 49.2%, and the pairing with SLXMK received a SUCRA value of 39.6%. The combination of TT and XZK was noted with a SUCRA value of 32.6%. Lastly, TT monotherapy was at the base of the rankings with a SUCRA value of 0.9%.

#### 4.2.4 LDL-C

LDL-C was delineated in 56 studies. The interventions pertained to 8 TCMs, encompassing 9 intervention strategies, and involved a cohort of 5552 participants. A nine-node network relationship had been constructed in Figure 3E. The most extensive research had been dedicated to the comparative efficacy of the combination therapy of TT and XZK versus TT monotherapy, with a total of 21 studies contributing to this comparison.

##### 4.2.4.1 Direct meta-analysis of LDL-C

The direct meta-analysis of LDL-C encompassed 8 pairs of comparisons, utilizing MD as the effect size. The forest plot was shown in Supplementary Material S7.5. It presented a comparative evaluation of the efficacy between the combination therapy of TCMs with TT and TT monotherapy. The findings revealed that the LDL-C for the combination of TT and DTJZ, JZL, JZTM, PS, XZK, and XZT consistently outperformed TT monotherapy, with significant statistical disparities (*P* < 0.05). Among them, the interventions of TT + DTJZ, TT + JZL, TT + PS, TT + XZK and TT + XZT resulted in considerable heterogeneity.

##### 4.2.4.2 Meta-regression and subgroup analysis of LDL-C

For the interventions of TT + PS and TT + XZK, meta-regression approach was performed to identify possible causes of heterogeneity. The results indicated that there existed statistical significance between the different subgroups. However, the subgroup analysis of TT + XZK based on different TT showed that, the corresponding *P*-value of different subgroups was 0.006, as shown in Supplementary Material S8.5.3. It might be the possible source of heterogeneity in TT + XZK.

As for the interventions of TT + DTJZ, TT + JZL, and TT + XZT, no enough studies were involved to adopt meta regression method. Subgroup analysis was performed to identify possible causes of heterogeneity, as depicted in Supplementary Material S8.5. The results indicated that, for TT + DTJZ, the heterogeneity may be relevant to the difference in sample size and whether or not combined with other diseases, as shown in Supplementary Material S8.5.1, 8.5.2. There was statistical significance between the two subgroups (*P <* 0.00001). Subgroup analysis of other interventions showed no significant reduction in heterogeneity and no statistical significance between subgroups.

Therefore, significant heterogeneity remained for the interventions TT + JZL, TT + JZTL, TT + PS, TT + SLXMK, and TT + XZK after subgroup analysis (*I* ^2^ = 56%, 50%, 91%, 87%, and 93%, respectively). The heterogeneity may be attributed to differences in statin types used, medication dosages, treatment duration, and total sample size. Specifically, in studies involving TT + JZL, the maximum TCM medication dose in Zhang XL’s study (Zhang et al., 2017) (16 g/day) may lead to an overestimation of the intervention group’s effect size. For TT + JZTL, the shorter treatment duration (8 weeks) in Xie Y’s study (Xie et al., 2014) compared to other studies (≥12 weeks) may cause an underestimation of the effect size. In studies involving TT + SLXMK, only Zhou Y’s study (Zhou, 2017) used atorvastatin as the control group treatment, which may lead to discrepancies in efficacy.

##### 4.2.4.3 Network meta-analysis of LDL-C

The NMA had yielded a total of 36 pairwise comparisons, with the MD for 9 of these comparisons exhibiting 95% CI that did not encompass 0, indicating statistically significant differences. The findings suggested that, in comparison to TT monotherapy, the combination of DTJZ, JZL,JZTM, XZK, and XZT with TT therapy can significantly enhance LDL-C, with the following MD and 95% CI values: [MD = −0.61, 95% CI (−0.9, −0.32)], [MD = −0.65, 95% CI (−0.96, −0.33)], [MD = −0.42, 95% CI (−0.75, −0.08)], [MD = −0.38, 95% CI (−0.5, −0.25)] and [MD = −0.5, 95% CI (−0.77, −0.23)], respectively. The therapeutic effects of DTJZ, JZL, XZK, and XZT when combined with TT were superior to the combination of PS with TT, with statistically significant differences, respectively represented as [MD = −0.47, 95% CI (−0.82, −0.12)], [MD = −0.51, 95% CI (−0.87, −0.13)], [MD = 0.24, 95% CI (0, 0.47)] and [MD = 0.36, 95% CI (0.03, 0.69)]. The remaining interventions showed no statistically significant differences in pairwise comparisons, as detailed in Table 3, the upper right triangular green region.

According to the rankings derived from SUCRA curve (Figure 4E), the hierarchy of interventions, based on LDL-C, was as follows: the combination of TT with JZL secured the top spot with a SUCRA value of 87.0%, followed closely by the combination with DTJZ at 83.8%. The XZT in conjunction with TT claimed the third position with a SUCRA value of 70.3%. TT paired with JZTL hold a SUCRA value of 57.5%, while the combination with XZK was credited with 50.9%. The integration of TT with SLXMK achieved a SUCRA value of 42.3%, and the pairing with JZTM received a SUCRA value of 37.8%. The combination of TT and PS was noted with a SUCRA value of 18.4%. Lastly, TT monotherapy was at the base of the rankings with a SUCRA value of 2.0%.

### 4.3 Safety outcomes

A total of 35 studies provided specific reports on the emergence of adverse reactions. Interventions included the combination of DTJZ, JZL, JZTL, JZTM, PS, SLXMK, XZK and XZT with TT, as well as treatment with TT monotherapy. Adverse reactions mainly included: abdominal bloating, abdominal pain, diarrhea, nausea, vomiting, muscle pain, joint swelling, muscle fatigue, headache, dizziness, rash, constipation, taste disturbances, and mild increases in liver and kidney function indicators, etc. Most symptoms were mild, resolved spontaneously, or disappeared after symptomatic treatment. The detailed summary table of adverse reactions were listed in Supplementary Material S13.

As shown in the forest plot (Supplementary Material S12), the combination therapy of TCMs with TT demonstrated a significantly lower incidence of adverse reactions compared with TT monotherapy, with statistical significance (OR = 0.69, 95% CI: 0.50–0.95, P < 0.05). Specifically, TT + XZK showed a statistically significant reduction in adverse reaction rates (OR = 0.43, 95% CI: 0.23–0.79, P < 0.05), while other TCMs + TT did not exhibit statistically significant differences in adverse reaction rates compared with TT monotherapy.

The additional risks of TCMs versus statins may primarily arise from specific constituents. Polygonum multiflorum (e.g., in JZL or PS) carries idiosyncratic hepatotoxicity risks. Blood-activating components like Salvia miltiorrhiza and Panax notoginseng may increase bleeding potential, while bitter/cold or greasy herbs such as Alisma orientale, Cassia seed, and Polygonatum commonly cause gastrointestinal discomfort. Critically, clinical evidence shows no statistically significant difference in overall adverse event rates between TT monotherapy and TT-TCM combinations. This suggests TCMs do not substantially increase risks. Notably, XZK (red yeast rice formulation) co-administered with statins may reduce TT-related adverse effects through synergistic mechanisms. Its multi-component profile exerts cytoprotective effects via phytosterol-mediated cholesterol absorption inhibition and flavonoid-provided antioxidant/anti-inflammatory activity. This directly ameliorates TT-associated myalgia and hepatic enzyme abnormalities.

These findings suggest that the combined use of TCMs with statins for hyperlipidemia treatment may potentially mitigate statin-related side effects without introducing additional safety concerns.

### 4.4 Sensitivity analysis

The sensitivity analysis was performed for each TCM in relation to the outcomes using the leave-one-out approach. The findings revealed that the pooled effect sizes, after the omission of each individual study, were consistent with the original effect sizes, and the corresponding statistical significance remained unchanged, as shown in Supplementary Material S9. Consequently, this demonstrated the robustness of meta-analysis.

### 4.5 Publication bias

Publication bias was performed with begg’s and egger’s test on comparisons including at least 3 RCTs, as shown in Supplementary Material S10. There was 38 comparisons, and only 3 results included 3 RCTs. Among them, *P*-values of begg’s test of all results were larger than 0.05, showing no statistical significance on publication bias. Egger’s test indicated that, 4 results owned a *P*-value less than 0.05, indicating possible publication bias. Most of the comparison results (89.5%, 34/38) did not indicate significant publication bias of included RCTs, showing the meta-analysis results were robust.

### 4.6 GRADE assessment

The GRADE assessment classified the evidence strengths into four tiers (high, moderate, low, and very low). When applied to assess the included RCTs, the evidence quality predominantly fell within the moderate to high range (seen Supplementary Material S11), suggesting generally positive results while acknowledging potential limitations. The limitations included: (1) 3 studies employed inadequate randomization method and 1 analyses reported participant withdrawal; (2) heterogeneity tests and subgroup analyses in 21 of the 40 effect estimates (derived from 8 interventions across 5 outcome measures) demonstrated data inconsistency; (3) 4 comparisons suggested potential publication bias; and (4) 2 analyses exhibited imprecision due to inadequate sample sizes (<250 participants). To enhance methodological rigor, future studies require standardized randomization methods, pre-specified subgroup analyses to address heterogeneity, and large-scale sample designs to improve precision. Systematic implementation of these methodological improvements is expected to elevate the evidence strength of future studies to a higher level, thereby significantly enhancing the reliability of clinical decision-making.

## 5 Discussion

Hyperlipidemia refers to a pathological state where blood lipid is abnormally fluctuated due to the genetic factors, metabolic disorders, and unhealthy lifestyle habits. In recent years, due to its potential threat to public health, hyperlipidemia has gradually become one of the hot topics in the field of global public health (Canfora and Pierno, 2024). Traditional Chinese medicine associates hyperlipidemia with spleen and stomach dysfunction, internal generation of phlegm and dampness, and decline of kidney. In addition, emotional fluctuations, excessive fatigue, and unhealthy lifestyle habits such as preference for greasy and sweet foods can interfere with the normal functions of the viscera, and ultimately promoting the occurrence and development of the disease (Xie et al., 2012).

TCMs demonstrate unique advantages over statins via standardized multi-target formulations and natural components with metabolic pleiotropy. Their combination strategy enhances lipid-lowering efficacy while reducing hepatotoxicity and myopathy risks (Ding et al., 2024). Based on preliminary literature research, as well as the principles of maximizing coverage of high-frequency components while minimizing ingredient redundancy, 8 TCMs was determined (Dantian Jiangzhi Pill, Jiangzhiling Tablet, Jiangzhi Tongluo Soft Capsule, Jiangzhi Tongmai Capsule, Pushen Capsule, Songling Xuemaikang Capsule, Xuezhikang Capsule, and Xuezhitong Capsule). This study conducted a NMA on the efficacy of these 8 included patent medicines combined with statins.

The results of the NMA indicated that in terms of improving the clinical total effective rate, the combination of statins and Jiangzhi Tongmai Capsule was ranked the best in the SUCRA ranking. The main ingredients of Jiangzhi Tongmai Capsule include Cassia tora, Alisma plantago-aquatica, and turmeric, which are used to resolve phlegm and dampness, activate blood circulation, and unblock collaterals, mainly used for hyperlipidemia caused by phlegm and blood stasis obstruction. Pharmacological studies have shown that Jiangzhi Tongmai Capsule can exert its effects through multiple active components and mechanisms. The lipid-lowering components of Cassia tora are primarily found in solvents with higher polarity, such as ethanol, ethyl acetate, n-butanol, and water, which can significantly reduce serum levels of LDL-C and TG, while increasing HDL-C (Yadav et al., 2010). Plantago asiatica mainly contains oxidized monoterpenes, particularly patchouli alcohol, which accounts for up to 82.6% of its composition. Patchouli alcohol can directly downregulate the expression of the cholesterol regulatory element-binding protein gene (Wen et al., 2022). Curcumin in turmeric can lower the levels of triglycerides and total cholesterol in the plasma and liver, repair impaired leptin signaling pathways in the liver, reduce SCD-1 gene expression, increase PPAR-α gene expression, and inhibit the development of fatty liver (Krishi et al., 2018).

In terms of reducing the TC, the combination of statins and Dantian Jiangzhi Pill was ranked the best in the SUCRA ranking. The main ingredients of Dantian Jiangzhi Pill include Salvia miltiorrhiza and Notoginseng, which have the effects of activating blood circulation to remove blood stasis, strengthening the spleen and kidney, reducing serum lipids, and improving microcirculation. The pharmacological effects of Dantian Jiangzhi Pill may involve multiple active components and mechanisms, and studies have shown that traditional Chinese medicine components such as Salvia miltiorrhiza (Ren et al., 2019) and Notoginseng (Ng, 2006) may regulate blood lipids through anti-inflammatory effects on inflammatory signaling pathways. These studies indicated Salvia miltiorrhiza enhances PGC-1α in the liver to improve lipid metabolism, reducing cholesterol accumulation, and Notoginseng reduces serum cholesterol by accelerating lipid excretion, achieving lipid-lowering effects.

In terms of reducing the TG, the combination of statins and Pushen Capsule was ranked the best in the SUCRA ranking. The main ingredients of Pushen Capsule include Salvia miltiorrhiza and Crataegus pinnatifida, which have the effects of activating blood circulation to remove blood stasis and nourishing yin to resolve turbidity. Pharmacological studies on Pushen Capsule have shown that it may exert its effects through the AGE-RAGE signaling pathway, affecting the occurrence and development of chronic inflammation, thereby improving blood lipid levels. Salvia miltiorrhiza and Crataegus pinnatifida are rich in bioactive compounds that lower blood lipid levels. Salvia miltiorrhiza inhibits cholesterol synthesis, while Crataegus pinnatifida’s anthocyanins enhance fat metabolism and reduce fat accumulation (Chen et al., 2013).

In terms of increasing the HDL-C and reducing the LDL-C, the combination of statins and Jiangzhiling Tablet was ranked the best in the SUCRA ranking. The main ingredients of Jiangzhiling Tablet include Cassia tora, Crataegus pinnatifida, and Polygonum multiflorum, which have the effects of nourishing the liver and kidney, nourishing blood and improving vision, and reducing blood lipids, suitable for patients with hyperlipidemia of liver and kidney yin deficiency type. Pharmacological studies on Jiangzhiling Tablet have shown that components such as emodin in processed Polygonum multiflorum may exert their effects through signaling pathways such as PI3K-Akt, estrogen, and AMPK, affecting lipid metabolic pathways to regulate blood lipids. The lipid-lowering effects of Polygonum multiflorum are primarily mediated by its active component 2,3,5,4′-tetrahydroxystilbene-2-O-β-D-glucoside (TSG), which regulates the expression of LDL-R mRNA, thereby reducing TC and LDL-C (Lin et al., 2015). Crataegus pinnatifida flavonoids may inhibit the rate-limiting enzyme in cholesterol synthesis in hepatic microsomes and intestinal mucosa, significantly increasing HDL-C and decreasing LDL-C (Li R. et al., 2023). Additionally, the research have shown that Cassia tora can also reduce LDL-C and increase HDL-C (Yadav et al., 2010).

The strengths and novelty of this study could be summarized as followed. (1) Systematically evaluation for the first time: The framework integrates high-frequency TCM screening, followed by NMA for efficacy ranking, subsequently validated through multidimensional methods, and finally explores pharmacological mechanisms. (2) Comprehensive database search: The study conducted an exhaustive search across 7 major databases, ensuring the completeness of the data included, thereby enhancing the comprehensiveness and reliability of the research findings. (3) Literature bias analysis: The study employed the Cochrane Risk of Bias tool for a rigorous evaluation of the quality of included studies, and created bias risk diagrams and traffic plot to thoroughly analyze literature bias. (4) Comprehensive outcomes: The research covered 5 major outcomes affecting blood lipid level, providing a comprehensive evaluation of treatment effects. (5) Overall efficacy comparison: Through NMA, including network evidence plots, league tables, and SUCRAs, the study comprehensively analyzed and compared the overall efficacy of various interventions. (6) Diverse assessment methods: The study utilized a variety of assessment methods, including meta-regression, subgroup analysis, sensitivity analysis, and publication bias analysis, to determine the sources of heterogeneity and to verify the robustness of the results. (7) Evidence strength evaluation: The study employed the GRADE approach to assess the evidence strength and analyzed the reasons for downgrading the evidence strength, offering targeted suggestions for future research directions.

In the methodological quality assessment of this study, we realized that there may be some limiting factors that could affect our comprehensive understanding of the study results. (1) Geographical limitation: As all included RCTs were conducted exclusively in China, this geographical constraint may limit the extrapolation of findings to other populations. (2) Sample size limitations: We noticed that the sample size for each intervention included in this study was relatively small, which may limit our ability to detect small effect size changes. (3) Randomization issues: In the included studies, some RCTs failed to clarify the specific randomization method and the statistical significance of the grouping results. These methodological gaps may introduce bias, affecting the objectivity and reliability of the results. (4) Publication bias and small sample effects: we found that there may be publication bias, and the small sample effect may have influenced our conclusions. Future studies should be based on multinational cohorts, large sample sizes, rigorous randomization protocols, as well as RCTs with multiple outcomes to enhance the practicality of the NMA results.

In clinical practice, due to differences in patients’ constitution, disease condition, and treatment response, individualized treatment is particularly important. This study provided efficacy data for the combined use of various TCMs with statins in the treatment of hyperlipidemia, which helps doctors choose the appropriate patent medicine according to the specific situation of patients to achieve differentiated treatment.

## 6 Conclusion

In this NMA involving patients with hyperlipidemia, Jiangzhi Tongmai Capsule had the best clinical total effective rate, Dantian Jiangzhi Pill was the most effective in reducing TC, Pushen Capsule was the most effective in reducing TG, and Jiangzhiling Tablet was the most effective in increasing HDL-C and reducing LDL-C. The results enable data-driven optimization of TCM-statin combination therapies to address personalized lipid management needs. We suggest that future studies should be based on large sample sizes, rigorously designed, and RCTs with multiple outcomes for verification.

## Data Availability

The original contributions presented in the study are included in the article/Supplementary Material, further inquiries can be directed to the corresponding authors.

## Abbreviations

CBM, Chinese Biomedical Literature Database; CI, confidence interval; CNKI, China National Knowledge Infrastructure; TCM, Traditional Chinese Medicine; DTJZ, Dantian Jiangzhi Pill; GRADE, Grading of Recommendations, Assessment, Development, and Evaluation; HDL-C, high-density lipoprotein cholesterol; IHD, ischemic heart disease; JZL, Jiangzhi Ling Tablet; JZTL, Jiangzhi Tongluo Soft Capsule; JZTM, Jiangzhi Tongmai Capsule; LDL-C, low-density lipoprotein cholesterol; MD, mean difference; NMA, network meta-analysis; OR, odds ratio; PS, Pushen Capsule; PSRF, Potential Scale Reduction Factor; RCT, randomized controlled trial; SLXMK, Songling Xuemaikang Capsule; SUCRA, Surface Under the Cumulative Ranking curve; TC, total cholesterol; TG, triglycerides; TT, traditional statin drug; VIP, Database of Chinese Sci-Tech Periodicals; XZK, Xuezhikang Capsule; XZT, Xuezhitong Capsule.
