# Supplementary material for: Comparative efficacy of eight traditional Chinese medicines combined with statins in the treatment of hyperlipidemia: a Bayesian network meta-analysis
Source: Front Pharmacol. 2025 Aug 5;16:1614767. doi: 10.3389/fphar.2025.1614767 (PMC12361126; doi:10.3389/fphar.2025.1614767)
Supplement: Supplementary file 2 [file DataSheet3.pdf]

Supplementary material

S1. PRISMA guideline.

S2. Preliminary literature research of TCMs in treating hyperlipidemia.

S3. Details of the included TCMs.

S4. Search strategy in different database.

S5. Details of the included studies.

S6. Traffic plot of bias risk assessment.

S7. Forest plot based on the difference in intervention measures.

S8. Heterogeneity analysis and subgroup analysis of different interventions for different outcomes.

S9. Sensitivity analysis for different outcomes.

S10. Publication bias analysis.

S11. GRADE assessment.

S12. Forest plot of safety outcomes.

S13. Summary table of adverse reactions.

S1. PRISMA guideline

S1.1 The PRISMA 2020 checklist.

Section and Topic	Item #	Checklist item	Location where item is reported
**TITLE**			
Title	1	Identify the report as a systematic review.	Title
**ABSTRACT**			
Abstract	2	See the PRISMA 2020 for Abstracts checklist.	Abstract
**INTRODUCTION**			
Rationale	3	Describe the rationale for the review in the context of existing knowledge.	Introduction
Objectives	4	Provide an explicit statement of the objective(s) or question(s) the review addresses.	Inclusion and exclusion criteria
**METHODS**			
Eligibility criteria	5	Specify the inclusion and exclusion criteria for the review and how studies were grouped for the syntheses.	Inclusion and exclusion criteria
Information sources	6	Specify all databases, registers, websites, organisations, reference lists and other sources searched or consulted to identify studies. Specify the date when each source was last searched or consulted.	Search strategy
Search strategy	7	Present the full search strategies for all databases, registers and websites, including any filters and limits used.	Search strategy
Selection process	8	Specify the methods used to decide whether a study met the inclusion criteria of the review, including how many reviewers screened each record and each report retrieved, whether they worked independently, and if applicable, details of automation tools used in the process.	Literature screening and data extraction
Data collection process	9	Specify the methods used to collect data from reports, including how many reviewers collected data from each report, whether they worked independently, any processes for obtaining or confirming data from study investigators, and if applicable, details of automation tools used in the process.	Literature screening and data extraction
Data items	10a	List and define all outcomes for which data were sought. Specify whether all results that were compatible with each outcome domain in each study were sought (e.g. for all measures, time points, analyses), and if not, the methods used to decide which results to collect.	Literature search results
	10b	List and define all other variables for which data were sought (e.g. participant and intervention characteristics, funding sources). Describe any assumptions made about any missing or unclear information.	Basic characteristics of included studies, Funding
Study risk of bias assessment	11	Specify the methods used to assess risk of bias in the included studies, including details of the tool(s) used, how many reviewers assessed each study and whether they worked independently, and if applicable, details of automation tools used in the process.	Data synthesis analysis
Effect measures	12	Specify for each outcome the effect measure(s) (e.g. risk ratio, mean difference) used in the synthesis or presentation of results.	Data synthesis analysis
Synthesis methods	13a	Describe the processes used to decide which studies were eligible for each synthesis (e.g. tabulating the study intervention characteristics and comparing against the planned groups for each synthesis (item #5)).	Statistical analysis

Section and Topic	Item #	Checklist item	Location where item is reported
	13b	Describe any methods required to prepare the data for presentation or synthesis, such as handling of missing summary statistics, or data conversions.	Statistical analysis
	13c	Describe any methods used to tabulate or visually display results of individual studies and syntheses.	Statistical analysis
	13d	Describe any methods used to synthesize results and provide a rationale for the choice(s). If meta-analysis was performed, describe the model(s), method(s) to identify the presence and extent of statistical heterogeneity, and software package(s) used.	Statistical analysis
	13e	Describe any methods used to explore possible causes of heterogeneity among study results (e.g. subgroup analysis, meta-regression).	Statistical analysis
	13f	Describe any sensitivity analyses conducted to assess robustness of the synthesized results.	Statistical analysis
Reporting bias assessment	14	Describe any methods used to assess risk of bias due to missing results in a synthesis (arising from reporting biases).	Quality assessment and evidence evaluation
Certainty assessment	15	Describe any methods used to assess certainty (or confidence) in the body of evidence for an outcome.	Data synthesis analysis
**RESULTS**			
Study selection	16a	Describe the results of the search and selection process, from the number of records identified in the search to the number of studies included in the review, ideally using a flow diagram.	Literature search results
	16b	Cite studies that might appear to meet the inclusion criteria, but which were excluded, and explain why they were excluded.	Literature search results
Study characteristics	17	Cite each included study and present its characteristics.	Basic characteristics of included studies
Risk of bias in studies	18	Present assessments of risk of bias for each included study.	Risk of bias assessment of included studies
Results of individual studies	19	For all outcomes, present, for each study: (a) summary statistics for each group (where appropriate) and (b) an effect estimate and its precision (e.g. confidence/credible interval), ideally using structured tables or plots.	Meta-analysis
Results of syntheses	20a	For each synthesis, briefly summarise the characteristics and risk of bias among contributing studies.	Risk of bias assessment of included studies
	20b	Present results of all statistical syntheses conducted. If meta-analysis was done, present for each the summary estimate and its precision (e.g. confidence/credible interval) and measures of statistical heterogeneity. If comparing groups, describe the direction of the effect.	Meta-analysis
	20c	Present results of all investigations of possible causes of heterogeneity among study results.	Meta-analysis
	20d	Present results of all sensitivity analyses conducted to assess the robustness of the synthesized results.	Sensitivity analysis
Reporting biases	21	Present assessments of risk of bias due to missing results (arising from reporting biases) for each synthesis assessed.	Publication

Section and Topic	Item #	Checklist item	Location where item is reported
			bias
Certainty of evidence	22	Present assessments of certainty (or confidence) in the body of evidence for each outcome assessed.	Meta-analysis
**DISCUSSION**			
Discussion	23a	Provide a general interpretation of the results in the context of other evidence.	Discussion
	23b	Discuss any limitations of the evidence included in the review.	Discussion
	23c	Discuss any limitations of the review processes used.	Discussion
	23d	Discuss implications of the results for practice, policy, and future research.	Discussion
**OTHER INFORMATION**			
Registration and protocol	24a	Provide registration information for the review, including register name and registration number, or state that the review was not registered.	Materials and methods
	24b	Indicate where the review protocol can be accessed, or state that a protocol was not prepared.	Materials and methods
	24c	Describe and explain any amendments to information provided at registration or in the protocol.	NA
Support	25	Describe sources of financial or non-financial support for the review, and the role of the funders or sponsors in the review.	Funding
Competing interests	26	Declare any competing interests of review authors.	Conflict of interest
Availability of data, code and other materials	27	Report which of the following are publicly available and where they can be found: template data collection forms; data extracted from included studies; data used for all analyses; analytic code; any other materials used in the review.	Supplementary data

For more information, visit: www.prisma-statement.org.

S1.2 PRISMA NMA checklist.

Section/Topic	Item #	Checklist Item	Reported on Page #
**TITLE**			
Title	1	Identify the report as a systematic review *incorporating a network meta-analysis (or related form of meta-analysis).*	***Title***
**ABSTRACT**			
Structured summary	2	Provide a structured summary including, as applicable:<br>**Background:** main objectives<br>**Methods:** data sources; study eligibility criteria, participants, and interventions; study appraisal; and *synthesis methods, such as network meta-analysis.*<br>**Results:** number of studies and participants identified; summary estimates with corresponding confidence/credible intervals; *treatment rankings may also be discussed. Authors may choose to summarize pairwise comparisons against a chosen treatment included in their analyses for brevity.*<br>**Discussion/Conclusions:** limitations; conclusions and implications of findings.<br>**Other:** primary source of funding; systematic review registration number with registry name.	Abstract
**INTRODUCTION**			
Rationale	3	Describe the rationale for the review in the context of what is already known*, including mention of why a network meta-analysis has been conducted.*	***Introduction***
Objectives	4	Provide an explicit statement of questions being addressed, with reference to participants, interventions, comparisons, outcomes, and study design (PICOS).	Inclusion and exclusion criteria
**METHODS**			
Protocol and registration	5	Indicate whether a review protocol exists and if and where it can be accessed (e.g., Web address); and, if available, provide registration information, including registration number.	Materials and methods
Eligibility criteria	6	Specify study characteristics (e.g., PICOS, length of follow-up) and report characteristics (e.g., years considered, language, publication status) used as criteria for eligibility, giving rationale. *Clearly describe eligible treatments included in the treatment network, and note whether any have been clustered or merged into the same node (with justification).*	***Inclusion and exclusion criteria***
Information sources	7	Describe all information sources (e.g., databases with dates of coverage, contact with study authors to identify additional studies) in the search and date last searched.	Search strategy
Search	8	Present full electronic search strategy for at least one database, including any limits used, such that it could be repeated.	Search strategy

Study selection	9	State the process for selecting studies (i.e., screening, eligibility, included in systematic review, and, if applicable, included in the meta-analysis).	Literature screening and data extraction
Data collection process	10	Describe method of data extraction from reports (e.g., piloted forms, independently, in duplicate) and any processes for obtaining and confirming data from investigators.	Literature screening and data extraction
Data items	11	List and define all variables for which data were sought (e.g., PICOS, funding sources) and any assumptions and simplifications made.	Basic characteristics of included studies, Funding
**Geometry of the network**	**S1**	Describe methods used to explore the geometry of the treatment network under study and potential biases related to it. This should include how the evidence base has been graphically summarized for presentation, and what characteristics were compiled and used to describe the evidence base to readers.	***Statistical analysis***
Risk of bias within individual studies	12	Describe methods used for assessing risk of bias of individual studies (including specification of whether this was done at the study or outcome level), and how this information is to be used in any data synthesis.	Quality assessment and evidence evaluation
Summary measures	13	State the principal summary measures (e.g., risk ratio, difference in means). *Also describe the use of additional summary measures assessed, such as treatment rankings and surface under the cumulative ranking curve (SUCRA) values, as well as modified approaches used to present summary findings from meta-analyses.*	Statistical analysis
Planned methods of analysis	14	Describe the methods of handling data and combining results of studies for each network meta-analysis. This should include, but not be limited to:<br>• *Handling of multi-arm trials;*<br>• *Selection of variance structure;*<br>• *Selection of prior distributions in Bayesian analyses; and*<br>• *Assessment of model fit.*	Statistical analysis
**Assessment of Inconsistency**	**S2**	Describe the statistical methods used to evaluate the agreement of direct and indirect evidence in the treatment network(s) studied. Describe efforts taken to address its presence when found.	Inclusion and exclusion criteria
Risk of bias across studies	15	Specify any assessment of risk of bias that may affect the cumulative evidence (e.g., publication bias, selective reporting within studies).	**Quality assessment and evidence evaluation**
Additional analyses	16	Describe methods of additional analyses if done, indicating which were pre-specified. This may include, but not be limited to, the following:<br>• Sensitivity or subgroup analyses;	***Statistical analysis***

- Meta-regression analyses;
- *Alternative formulations of the treatment network; and*
- *Use of alternative prior distributions for Bayesian analyses (if applicable).*

RESULTS†

Study selection	17	Give numbers of studies screened, assessed for eligibility, and included in the review, with reasons for exclusions at each stage, ideally with a flow diagram.	Literature search results
**Presentation of network structure**	**S3**	Provide a network graph of the included studies to enable visualization of the geometry of the treatment network.	***Meta-analysis***
**Summary of network geometry**	**S4**	Provide a brief overview of characteristics of the treatment network. This may include commentary on the abundance of trials and randomized patients for the different interventions and pairwise comparisons in the network, gaps of evidence in the treatment network, and potential biases reflected by the network structure.	***Meta-analysis***
Study characteristics	18	For each study, present characteristics for which data were extracted (e.g., study size, PICOS, follow-up period) and provide the citations.	Basic characteristics of included studies
Risk of bias within studies	19	Present data on risk of bias of each study and, if available, any outcome level assessment.	Risk of bias assessment of included studies
Results of individual studies	20	For all outcomes considered (benefits or harms), present, for each study: 1) simple summary data for each intervention group, and 2) effect estimates and confidence intervals. *Modified approaches may be needed to deal with information from larger networks.*	Meta-analysis
Synthesis of results	21	Present results of each meta-analysis done, including confidence/credible intervals. *In larger networks, authors may focus on comparisons versus a particular comparator (e.g. placebo or standard care), with full findings presented in an appendix. League tables and forest plots may be considered to summarize pairwise comparisons.* If additional summary measures were explored (such as treatment rankings), these should also be presented.	***Meta-analysis***
**Exploration for inconsistency**	**S5**	Describe results from investigations of inconsistency. This may include such information as measures of model fit to compare consistency and inconsistency models, *P* values from statistical tests, or summary of inconsistency estimates from different parts of the treatment network.	***Meta-analysis***
Risk of bias across studies	22	Present results of any assessment of risk of bias across studies for the evidence base being studied.	Publication bias
Results of additional analyses	23	Give results of additional analyses, if done (e.g., sensitivity or subgroup analyses, meta-regression analyses*, alternative network geometries studied, alternative choice of prior distributions for Bayesian analyses,* and so forth).	***Meta-analysis***

DISCUSSION

Summary of evidence	24	Summarize the main findings, including the strength of evidence for each main outcome; consider their relevance to key groups (e.g., healthcare providers, users, and policy-makers).	Discussion
Limitations	25	Discuss limitations at study and outcome level (e.g., risk of bias), and at review level (e.g., incomplete retrieval of identified research, reporting bias). *Comment on the validity of the assumptions, such as transitivity and consistency. Comment on any concerns regarding network geometry (e.g., avoidance of certain comparisons).*	Discussion
Conclusions	26	Provide a general interpretation of the results in the context of other evidence, and implications for future research.	Conclusion
**FUNDING**			
Funding	27	Describe sources of funding for the systematic review and other support (e.g., supply of data); role of funders for the systematic review. This should also include information regarding whether funding has been received from manufacturers of treatments in the network and/or whether some of the authors are content experts with professional conflicts of interest that could affect use of treatments in the network.	***Funding***

PICOS = population, intervention, comparators, outcomes, study design.

* Text in italics indicate wording specific to reporting of network meta-analyses that has been added to guidance from the PRISMA statement.

† Authors may wish to plan for use of appendices to present all relevant information in full detail for items in this section.

S2. Preliminary literature research of TCMs in treating hyperlipidemia.

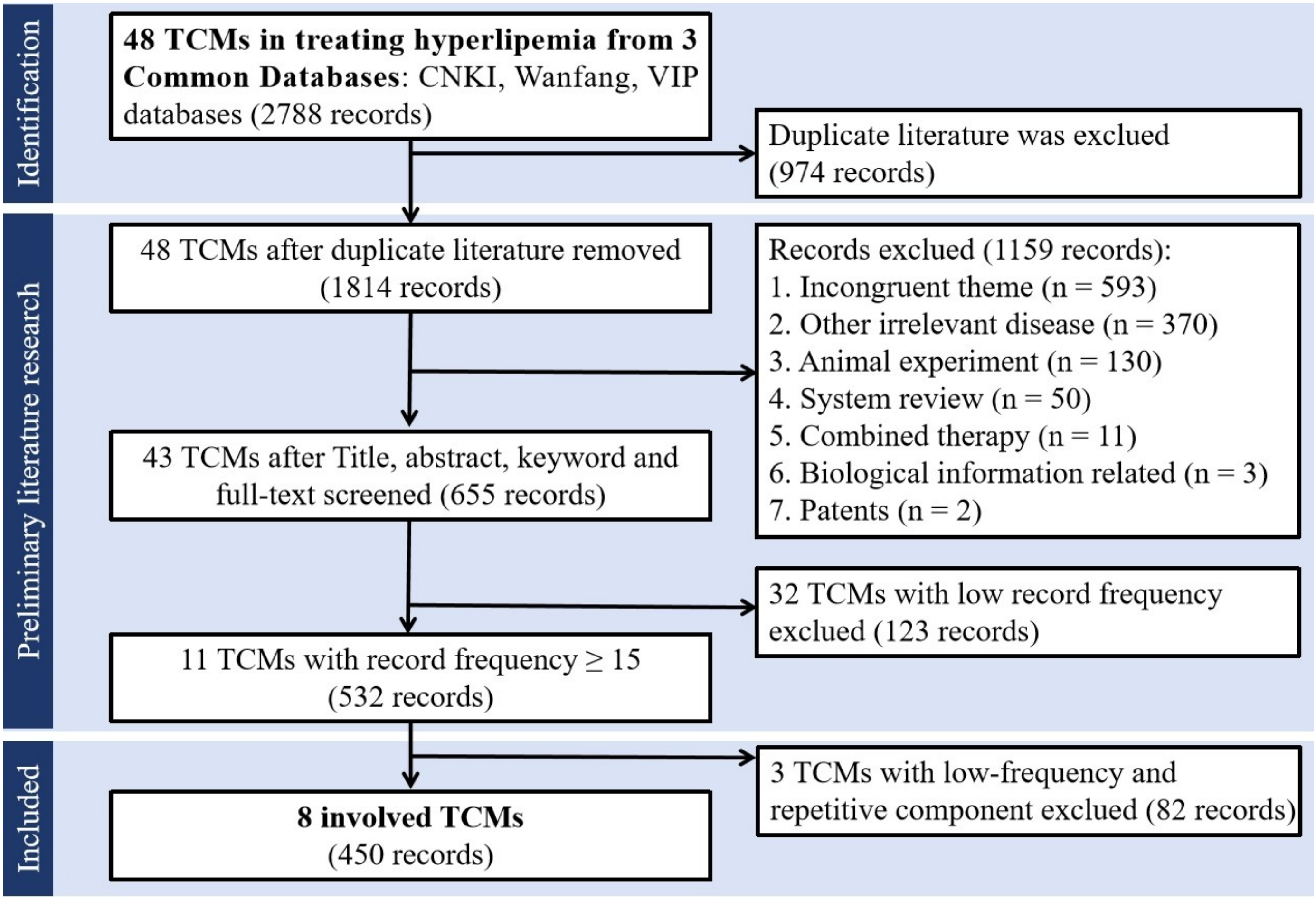

S3. Details of the included TCMs.

S3.1 The source, approval information and raw material of included TCMs.

TCMs	Source	SFDA approval number and date	Raw material	Quality control reported (Y/N)
Dantian Jiangzhi Pill	Guangdong Hongxing Group Co., Ltd. Hongxing Pharmaceutical Factory	Z44021107	Salvia miltiorrhiza Bunge [Lamiaceae; Salviae miltiorrhizae radix et rhizoma]; Panax notoginseng (Burkill) F.H.Chen ex C.Y.Wu & K.M.Feng [Araliaceae; Notoginseng radix]; Polygonum multiflorum Thunb. [Polygonaceae; Polygoni multiflori radix preparata]; Panax ginseng C.A.Mey. [Araliaceae; Ginseng radix et rhizoma]; Ligusticum chuanxiong S.H.Qiu, Y.Q.Zeng, K.Y.Pan, Y.C.Tang & J.M.Xu [Apiaceae; Chuanxiong rhizoma]; Alisma plantago-aquatica L. [Alismataceae; Alismatis rhizoma]; Angelica sinensis (Oliv.) Diels [Apiaceae; Angelicae sinensis radix]; Polygonatum sibiricum Redouté [Asparagaceae; Polygonati rhizoma]; Cinnamomum cassia (L.) J.Presl [Lauraceae; Cinnamomi cortex]; Epimedium brevicornu Maxim. [Berberidaceae; Epimedii folium]; Eleutherococcus senticosus (Rupr. & Maxim.) Maxim. [Araliaceae; Eleutherococci radix]	Y*
Jiangzhiling Tablet	Taiji Group Chongqing Tongjun Palace Pharmaceutical Factory Co., Ltd.	Z50020125	Polygonum multiflorum Thunb. [Polygonaceae; Polygoni multiflori radix preparata]; Lycium barbarum L. [Solanaceae; Lycii fructus]; Polygonatum sibiricum Redouté [Asparagaceae; Polygonati rhizoma]; Crataegus pinnatifida Bunge [Rosaceae; Crataegi fructus]; Cassia tora L. [Fabaceae; Cassiae semen]	Y*
Jiangzhi Tongluo Soft Capsules	Sunway Pharmaceutical Group Co., LTD	Z20040032	Curcuma longa L. [Zingiberaceae; Curcumae longae rhizoma]	Y*
Jiangzhi Tongmai Capsule	Yunnan excellent control drug company	Z20026429	Cassia tora L. [Fabaceae; Cassiae semen]; Curcuma longa L. [Zingiberaceae; Curcumae longae rhizoma]; Alisma plantago-aquatica L. [Alismataceae; Alismatis rhizoma]; Panax notoginseng (Burkill) F.H.Chen ex C.Y.Wu & K.M.Feng [Araliaceae; Notoginseng radix]; Lysimachia clethroides Duby [Primulaceae; Lysimachiae herba]	Y*
Pushen Capsule	Suzhong Pharmaceutical Group Co., LTD	Z20040074	Polygonum multiflorum Thunb. [Polygonaceae; Polygoni multiflori radix preparata]; Typha angustifolia L. [Typhaceae; Typhae pollen]; Salvia miltiorrhiza Bunge [Lamiaceae; Salviae miltiorrhizae radix et rhizoma]; Ligusticum chuanxiong S.H.Qiu, Y.Q.Zeng, K.Y.Pan, Y.C.Tang & J.M.Xu [Apiaceae; Chuanxiong rhizoma]; Paeonia lactiflora Pall. [Paeoniaceae; Paeoniae radix rubra]; Crataegus pinnatifida Bunge [Rosaceae; Crataegi fructus]; Alisma plantago-aquatica L. [Alismataceae; Alismatis rhizoma]; Codonopsis pilosula (Franch.) Nannf. [Campanulaceae; Codonopsis radix]	Y*

Songling Xuemaikang Capsule	Sichuan Jisheng Tang Pharmaceutical Co., LTD	Z10960023	Pinus massoniana Lamb. [Pinaceae; Pini massonianae folium]; Pueraria lobata (Willd.) Ohwi [Fabaceae; Puerariae radix]; Pinctada martensii (Dunker) [Pteriidae; Margaritifera concha pulverata]	Y*
Xuezhikang Capsule	Beijing Beida Weixin Biotechnology Co., LTD	Z10950029	Monascus purpureus Went. [Monascaceae; Monasci purpurei fermentum]	Y*
Xuezhitong Capsule	Jilin Province Oriental Pharmaceutical Co., LTD	Z10970076	Allium macrostemon Bunge [Amaryllidaceae; Allii macrostemi bulbus]	Y*

*Prepared according to People's Republic of China Pharmacopoeia.

S3.2 Classification of TCM ingredients processing methods.

Processing Method	TCM ingredients
Steaming	Polygonum multiflorum, Polygonatum sibiricum, Panax ginseng, Allium macrostemon
Frying/Steaming	Panax notoginseng
Wine Processing	Salvia miltiorrhiza, Ligusticum chuanxiong, Angelica sinensis, Paeonia lactiflora
Stir-frying	Cinnamomum cassia, Alisma plantago-aquatica, Crataegus pinnatifida, Cassia tora, Typha angustifolia
Oil Processing	Epimedium brevicornu
Calcining	Pinus massoniana, Pinctada martensii
Roasting in Ashes	Pueraria lobata
Honey Processing	Codonopsis pilosula
Fermentation	Monascus purpureus
Sulfur Fumigation	Lycium barbarum
Vinegar Processing	Curcuma longa

S4. Search strategy in different database.

Database	Specific Search Query
PubMed	(((Hyperlipidemia OR Dyslipidemia) AND ((Xuezhikang) OR (Dantian Jiangzhi) OR (Jiangzhiling) OR (Pushen) OR (Songling Xuemaikang) OR (Jiangzhi Tongluo) OR (Jiangzhi Tongmai) OR (Xuezhitong))) AND (Randomized)) AND (Clinical trial)
Web of Science (WOS)	(((TS=(Hyperlipidemia OR Dyslipidemia)) AND TS=((Xuezhikang) OR (Dantian Jiangzhi) OR (Jiangzhiling) OR (Pushen) OR (Songling Xuemaikang) OR (Jiangzhi Tongluo) OR (Jiangzhi Tongmai) OR (Xuezhitong))) AND TS=(Randomized)) AND TS=(Clinical trial)
Cochrane Library	Hyperlipidemia OR Dyslipidemia in Title Abstract Keyword AND (Xuezhikang) OR (Dantian Jiangzhi) OR (Jiangzhiling) OR (Hedan) OR (Pushen) OR (Songling Xuemaikang) OR (Jiangzhi Tongluo) OR (Jiangzhi Tongmai) OR (Xuezhitong) in Title Abstract Keyword AND Randomized in Title Abstract Keyword AND clinical trial in Title Abstract Keyword
China National Knowledge Infrastructure (CNKI)	(篇关摘:高脂血症 + 高血脂 +血脂异常(模糊))AND(篇关摘:血脂康 +丹田降脂丸 + 降脂灵 + 蒲参 + 松龄血脉康 + 降脂通络 + 降脂通脉 + 血滞通(模糊))AND(篇关摘:随机(模糊))AND(篇关摘:临床(模糊))
Wanfang	全部:(高脂血症 OR 高血脂 OR 血脂异常) and 全部:(血脂康 OR 丹田降脂 OR 降脂灵 OR 蒲参 OR 松龄血脉康 OR 降脂通络 OR 降脂通脉 OR 血滞通) and 全部:(随机) and 全部:(临床)
Database of Chinese sci-tech periodicals (VIP)	任意字段=高脂血症 + 高血脂 +血脂异常 AND 任意字段=血脂康 + 丹田降脂丸 + 降脂灵 + 蒲参 + 松龄血脉康 + 降脂通络 + 降脂通脉 + 血滞通 AND 任意字段=随机 AND 任意字段=临床
Chinese Biomedical Literature Database (CBM)	(“高脂血症"[常用字段:智能] OR “高血脂"[常用字段:智能] OR “血脂异常”[常用字段:智能]) AND (“血脂康"[常用字段:智能] OR “丹田降脂” [常用字段:智能] OR “降脂灵” [常用字段:智能] OR “蒲参” [常用字段:智能] OR “松龄血脉康” [常用字段:智能] OR “降脂通络” [常用字段:智能] OR “降脂通脉” [常用字段:智能] OR “血滞通” [常用字段:智能]) AND “随机” [常用字段:智能] AND “临床” [常用字段:智能]

S5. Details of the included studies.

Study ID	Sample Size	Male/ Female		Mean Age ± Standard Deviation (SD)		Treatment group interventions	Duration (weeks)	Outcomes
		T	C	T	C			
Wang QC 2012[1]	60	30	30	78.8±6.9		TT+DTJZ	12	②③④⑤
Kuang DD 2019[2]	96	17/31	19/29	58.43±6.25	58.59±6.06	TT+DTJZ	12	①②③④⑤
Sun JY 2012[3]	90	26/19	25/20	49.02±9.19	48.23±9.44	TT+DTJZ	8	①②③④⑤
Lv WH 2013[4]	316	92/66	90/68	56.98±6.11	57.76±6.35	TT+DTJZ	4	①②③④
Liang ZM 2015[5]	112	29/27	28/28	68.4±4.1	69.3±5.2	TT+DTJZ	8	①②③④⑤
Zhang XL 2017[6]	100	25/25	23/27	56±10	55±11	TT+JZL	12	②③④⑤
Zhu TW 2021[7]	80	21/19	22/18	55.38±3.62	55.46±3.72	TT+JZL	12	①②③④⑤
Hu XW 2015[8]	98	35/13	29/21	37.42±4.87	36.79±7.11	TT+JZL	4	②③④⑤
Guo XP 2010[9]	68	34	34	43.26±9.12		TT+JZL	4	①②③④⑤
Bao FY 2009[10]	112	56	56	Not reported		TT+JZTL	16	①②③④
Xie Y 2014[11]	122	60	62	55.16±9.77	53.72±10.62	TT+JZTL	8	②③④⑤
Guo YW 2017[12]	116	32/26	30/28	64.28±1.65	64.26±1.62	TT+JZTL	12	②③④⑤
Wen CT 2013[13]	110	55	55	50.15±9.86	50.67±10.28	TT+JZTL	12	①②③④⑤
Xiao B 2014[14]	80	40	40	Not reported		TT+JZTM	8	①②③④⑤
Liu FG 2011[15]	60	30	30	Not reported		TT+JZTM	30d	②③④⑤
Lu HM 2021[16]	92	26/20	24/22	55.18±10.85	55.96±11.34	TT+JZTM	4	①②③④⑤
You HS 2022[17]	100	26/24	25/25	62.39±2.67	62.29±2.84	TT+JZTM	12	①②③④⑤
Wang H 2019[18]	30	9/6	6/9	67.6±8.3	68.2±8.3	TT+JZTM	8	②③④⑤
Gu SM 2015[19]	60	30	30	85		TT+PS	8	①②③④⑤
Shao ZX 2016[20]	105	38/32	21/14	63.91±7.95	61.49±9.42	TT+PS	12	②③④⑤
Li X 2016[21]	76	18/12	21/25	47.50±10.46	46.36±10.38	TT+PS	8	①②③④⑤
Chen X 2016[22]	40	20	20	61.54±10.65		TT+PS	8	②③④⑤
Cao CY 2014[23]	84	28/14	29/13	58	59	TT+PS	8	①②③④⑤
Zhang SY 2013[24]	60	15/15	13/17	61.8±10.3	61.0±9.4	TT+PS	8	②③④⑤
Cao XC 2021[25]	64	19/13	17/15	60.09±5.61	59.25±5.53	TT+PS	12	①②③④⑤

Mu M 2014[26]	86	25/18	23/20	61	55	TT+PS	8	①②③④⑤
Wu NJ2020[27]	79	21/19	20/19	44.8±6.5	45.6±6.4	TT+PS	Not reported	①②③④⑤
Yu GL 2013[28]	80	12/28	16/24	56.43±9.92	55.40±8.89	TT+PS	10	②③④⑤
Zhou Y 2017[29]	80	19/22	21/18	58.6±9.82	57.8±8.59	TT+SLXMK	24	②③④⑤
Ning Y 2012[30]	100	27/23	26/24	56±7	55±8	TT+SLXMK	4	①②③④⑤
Feng ZP 2013[31]	600	162/138	162/138	49.79±1.37	50.07±1.31	TT+SLXMK	8	①
Cai ZQ 2015[32]	96	26/22	29/19	63	61	TT+SLXMK	12	①②③④⑤
Li HL 2011[33]	120	63	57	72±3		TT+SLXMK	6	①②③④
Zhou P 2012[34]	70	19/16	21/14	40-70	41-70	TT+SLXMK	12	①
Chen Y 2019[35]	60	21/9	20/10	Not reported		TT+XZK	12	①
Gui YF 2019[36]	72	15/21	18/18	57.7±20.3	56±16.4	TT+XZK	8	②③
Wang ZZ 2013[37]	54	27	27	44.2±4.9		TT+XZK	12	①②③④⑤
Zhou M 2017[38]	488	134/110	140/104	52.6±5.4	53.1±5.5	TT+XZK	12	①②③④⑤
Ye JG 2021[39]	120	35/25	34/26	53.2±11.8	52.7±11.4	TT+XZK	2	①②③④⑤
Yu Q 2021[40]	76	18/20	20/18	58.69±6.44	58.46±6.28	TT+XZK	12	②③④⑤
Shi C 2018[41]	124	34/28	35/27	62.13±7.27	63.03±7.52	TT+XZK	8	①②③④⑤
Tian L 2022[42]	60	17/13	18/12	53.49±3.05	64.02±3.11	TT+XZK	12	①②③④⑤
Qu J 2020[43]	78	22/17	25/14	50.08±4.19	51.08±4.27	TT+XZK	12	①②④⑤
Xu BT 2021[44]	100	27/23	26/24	56.54±3.25	55.21±3.65	TT+XZK	24	①
Sun XJ 2018[45]	96	28/20	27/21	63.34±7.29	63.39±7.64	TT+XZK	12	①②③④⑤
Jiang CH 2012[46]	170	42/43	40/45	61.7±6.9	61.1±7.4	TT+XZK	12	①②③④⑤
Huang CC 2014[47]	55	18/12	17/8	59.5	58.2	TT+XZK	2	②③
Zhou Z 2010[48]	78	21/18	22/17	52.4±5.5	53.2±4.9	TT+XZK	4	①②③④⑤
Lian L 2015[49]	150	48/30	42/30	51.8±11.2	56.3±11.2	TT+XZK	4	①
Wei GR 2011[50]	76	38	38	Not reported		TT+XZK	4	②③④⑤
Chen HY 2014[51]	84	27/15	29/13	70.2±8.4	69.7±8.1	TT+XZK	8	①②③④⑤
Liu YS 2022[52]	118	36/23	39/20	63.72±5.48	64.02±5.88	TT+XZK	4	②③④⑤
Hu GY 2015[53]	120	34/26	29/31	53.1	57.5	TT+XZK	8	①②③④
Wang Z 2012[54]	78	39	39	59.7±6.4		TT+XZK	12	①②③④

								⑤
Jiang F 2013[55]	56	16/12	15/13	68.1±2.2	67.4±2.3	TT+XZK	12	①②③④⑤
Ma JB 2015[56]	80	16/24	17/23	40-70	40-70	TT+XZK	20	②③④⑤
Li RG 2023[57]	200	53/47	54/46	68.02±4.13	65.62±4.45	TT+XZK	4	①②③④⑤
Zhou L 2024[58]	82	23/18	22/19	51.34±4.12	52.01±4.06	TT+XZK	12	①②③④⑤
Fu YJ 2017[59]	150	36/39	38/37	63.2±9.3	61.8±9.3	TT+XZK	4	①②③④⑤
Xu XC 2024[60]	100	29/21	28/22	65.59±4.12	65.49±4.96	TT+XZK	4	①②③④⑤
Qiang SC 2023[61]	180	46/44	47/43	61.47±5.89	61.39±5.82	TT+XZK	24	②③④⑤
Shi WH 2011[62]	120	60	60	Not reported		TT+XZT	8	②③④⑤
Zhou HL 2015[63]	100	26/24	31/19	42.7±12.8	43.4±11.9	TT+XZT	16	①②③④⑤
Chen Q 2022[64]	60	15/15	19/11	51.2±8.1	51.8±8.5	TT+XZT	12	①②③④⑤
Yang DM 2014[65]	80	25/15	21/19	65.7±8.3	67.5±11	TT+XZT	12	①②③④⑤
Xiong G 2019[66]	100	50	50	Not reported		TT+XZT	8	①②③④⑤
Kuang ZB 2010[67]	120	36/24	35/25	54.5±3.4	53.8±4.2	TT+XZT	16	②③④⑤

Abbreviations: T-Treatment Group, C-Control Group, DTJZ-Dantian Jiangzhi Pill, JZL-Jiangzhiling Tablet, JZTL-Jiangzhi Tongluo Soft Capsule, JZTM-Jiangzhi Tongmai Capsule, PS-Pushen Capsule, SLXMK-Songling Xuemaikang Capsule, XZK-Xuezhikang Capsule, XZT-Xuezhitong Capsule, TT-Statin; ①Clinical Total Effective Rate, ②Total Cholesterol (TC) Index, ③Triglyceride (TG) Index, ④High-Density Lipoprotein Cholesterol (HDL-C) Index, ⑤Low-Density Lipoprotein Cholesterol (LDL-C) Index

References of included 67 RCTs:

[1] Wang, Q. C., Jiang, X. P., & Wu, D. H. (2012). Clinical observation of atorvastatin combined with Dantian Jiangzhi Pill in the treatment of elderly patients with hyperlipidemia. *Modern Journal of Integrated Traditional Chinese and Western Medicine*, 21(36), 4047-4048.
[2] Kuang, D. D. (2019). Randomized parallel controlled study of Dantian Jiangzhi Pill combined with atorvastatin in the treatment of hyperlipidemia. *Journal of Practical Traditional Chinese Internal Medicine*, 8.
[3] Sun, J. Y., Ma, X. M., Liu, Z. H., et al. (2012). Clinical observation of the combined use of Dantian Jiangzhi Pill and simvastatin in the treatment of hyperlipidemia. *Progress in Modern Biomedicine*, 12(31), 6138-6141.
[4] Lv, W. H. (2013). Efficacy observation of Dantian Jiangzhi Pill in the treatment of hyperlipidemia. *Journal of Clinical Rational Drug Use,* 6(21), 76-76.
[5] Liang, Z. M., & Jiang, L. P. (2015). Clinical efficacy of Dantian Jiangzhi Pill in the treatment of elderly patients with hyperlipidemia. *Chinese Journal of Gerontology,* 35(11), 3.
[6] Zhang, X. L., Shen, Y., Duan, X. B., et al. (2017). The impact of Jiangzhi Ling tablets combined with atorvastatin calcium on carotid plaques in patients with hyperlipidemia. *Journal of Integrated Traditional and Western Medicine on Cardiovascular and Cerebrovascular Diseases*, 15(9), 1083-1085.
[7] Zhu, T. W. (2021). The impact of Jiangzhi Ling tablets combined with rosuvastatin on serum indices in patients with hyperlipidemia. *Chinese Science and Technology Journal Database (Full Text Edition) Medical and Health*, 2021(10), 2.

[8] Hu, X. W. (2015). The impact of Jiangzhi Ling tablets combined with simvastatin on blood lipid and liver function indices in patients with fatty liver and hyperlipidemia. *Chinese Journal of Clinical Research*, 7(14), 78-79.
[9] Guo, X. P., Yuan, B. Z., & Cheng, Y. F. (2010). Clinical observation of Jiangzhi Ling tablets combined with simvastatin in the treatment of hyperlipidemia. *Journal of Integrated Traditional and Western Medicine on Cardiovascular and Cerebrovascular Diseases*, 8(12), 1426-1427.
[10] Bao, F. Y., & Wang, C. X. (2009). Efficacy observation of 56 cases treated with Jiangzhi Tongluo Capsule and atorvastatin for hyperlipidemia. *Shandong Medical Journal*, 49(19), 97-98.
[11] Xie, Y., He, Y. B., Zhang, S. X., et al. (2014). Clinical study of Jiangzhi Tongluo soft capsules combined with atorvastatin calcium tablets in the treatment of mixed hyperlipidemia. *Chinese Journal of Integrated Medicine*, 34(9), 1059-1063.
[12] Guo, Y. W. (2017). Clinical efficacy of Jiangzhi Tongluo soft capsules combined with rosuvastatin in the treatment of type 2 diabetes mellitus with hyperlipidemia. *Clinical Research*, 25(4), 93-94.
[13] Wen, C. T., & Han, Y. X. (2013). Efficacy observation of Jiangzhi Tongluo soft capsules combined with simvastatin in the treatment of hyperlipidemia. *Chinese and Foreign Health Digest*, 20(33), 102-102.
[14] Xiao, B. (2014). Clinical observation of atorvastatin calcium combined with Jiangzhi Tongmai Capsule in the treatment of hyperlipidemia. *Chinese Modern Doctor*, 52(15), 44-46.
[15] Liu, F. G., & Chen, J. (2011). Efficacy observation of Jiangzhi Tongmai Capsule combined with atorvastatin calcium in the treatment of mixed hyperlipidemia. *China Medical Guide*, 8(27), 71-72.
[16] Lu, H. M. (2021). Analysis of the effect and safety of Jiangzhi Tongmai Capsule combined with simvastatin in the treatment of hyperlipidemia. *Chinese and Foreign Medical Research*, 19(35), 4.
[17] You, H. S. (2022). Discussion on the effect and safety of Jiangzhi Tongmai Capsule combined with simvastatin in the treatment of hyperlipidemia. *Chinese Science and Technology Journal Database (Full Text Edition) Medical and Health*, 2022(9), 4.
[18] Wang, H. (2019). Clinical efficacy observation of Jiangzhi Tongmai combined with simvastatin in the treatment of hyperlipidemia. *China Health Care Nutrition*, 2019.
[19] Gu, S. M., & Sun, Y. (2015). Efficacy observation of Pu Shen Capsule in the treatment of elderly patients with hyperlipidemia. *People's Military Doctor*, 58(1), 71-72.
[20] Shao, X. Z., & Gu, N. (2016). The impact of Pu Shen Capsule combined with atorvastatin on blood lipid levels in patients with coronary heart disease and blood lipid disorders. *Journal of Integrated Traditional and Western Medicine on Cardiovascular and Cerebrovascular Diseases*, 14(3), 282-284.
[21] Li, X. (2016). Randomized parallel controlled study of Pu Shen Capsule combined with atorvastatin calcium in the treatment of hyperlipidemia. *Journal of Practical Traditional Chinese Internal Medicine*, (1), 73-75.
[22] Chen, X. (2016). Clinical study of Pu Shen Capsule combined with atorvastatin calcium in the treatment of mixed hyperlipidemia. *Journal of Integrated Traditional Chinese and Western Medicine on Cardiovascular and Cerebrovascular Diseases (Electronic Edition)*, 4(15), 191-192.
[23] Cao, C. Y., & Zhu, H. S. (2014). Clinical observation of Pu Shen Capsule combined with atorvastatin in the treatment of hyperlipidemia. **Practical Integrated Traditional Chinese and Western Medicine Clinic*, (6), 25-26.
[24] Zhang, S. Y., & Wu, Z. Y. (2013). Efficacy observation of Pu Shen Capsule combined with rosuvastatin calcium in the treatment of coronary heart disease with mixed hyperlipidemia. *Practical Cardiovascular and Pulmonary Vascular Disease Magazine*, 21(3), 52-54.
[25] Cao, X. C., Zhao, H. Z., Kong, D. Y., et al. (2021). Efficacy observation of Pu Shen Capsule combined with rosuvastatin calcium in the treatment of coronary artery slow flow with blood lipid disorders. *Journal of Integrated Traditional and Western Medicine on Cardiovascular and Cerebrovascular Diseases*, 19(11), 1861-1863.
[26] Mu, M. (2014). Treatment of 43 cases of middle-aged and elderly patients with mixed hyperlipidemia with Pu Shen Capsule combined with rosuvastatin tablets. *China Pharmaceutical News*, 23(24), 102-103.
[27] Wu, N. J. (2020). Clinical efficacy of Pu Shen Capsule combined with rosuvastatin in the treatment of type 2 diabetes mellitus with hyperlipidemia. *Journal of Qiqihar Medical College*, 41(20), 2551-2553.
[28] Yu, G. L., Yan, H., Hu, M. M., et al. (2013). Clinical observation of Pu Shen Capsule in the treatment of hyperlipidemia. *Journal of Nanjing University of Chinese Medicine*, 29(6), 594-595.

[29] Zhou, Y. (2017). Efficacy observation of Songling Blood Vessel Health Capsule combined with atorvastatin calcium tablets in the treatment ofpatients with blood lipid disorders and carotid atherosclerosis. *New Chinese Medicine*, 49(11), 15-17.
[30] Ning, Y., & Ding, H. L. (2012). Randomized controlled clinical trial of Songling Blood Vessel Health Capsule combined with simvastatin capsules in the treatment of hyperlipidemia (Liver Yang Hyperactivity Syndrome). *Seek Medical Advice and Ask for Medicine: The Lower Half Month*, 12, 767-768.
[31] Feng, Z. P. (2013). Randomized parallel controlled study of Songling Blood Vessel Health Capsule combined with simvastatin in the treatment of hyperlipidemia. *Journal of Practical Traditional Chinese Internal Medicine*, (5), 29-30.
[32] Cai, Z. Q., Yuan, H. Y., & Yu, Y. (2015). Clinical evaluation of 48 cases of angina pectoris with hyperlipidemia treated with Songling Blood Vessel Health Capsule combined with simvastatin. *China Pharmaceutical News*, 24(17), 29-30.
[33] Li, H. L., Liu, Y., & Zuo, X. W. (2011). Comparative efficacy of Songling Blood Vessel Health Capsule and Lescol in the treatment of elderly patients with hyperlipidemia. *Practical Cardiovascular and Pulmonary Vascular Disease Magazine*, 19(5), 769-770.
[34] Zhou, P. (2012). Efficacy observation of Songling Blood Vessel Health combined with simvastatin in the treatment of hyperlipidemia. *Practical Cardiovascular and Pulmonary Vascular Disease Magazine*, 20(7), 1203-1204.
[35] Chen, Y., Shen, J. Q., Luo, H., et al. (2019). Clinical analysis of atorvastatin and lipid-lowering capsule in the treatment of primary hyperlipidemia. *Health Preservation and Health Care Guide*, 2019.
[36] Gui, Y. F., & Zhang, C. L. (2019). Clinical study of patients with hyperlipidemia based on integrated traditional Chinese and Western medicine therapy. *World Latest Medicine Information*, 2019.
[37] Wang, Z. Z. (2013). Clinical efficacy observation of lipid-lowering capsule combined with atorvastatin in the treatment of hyperlipidemia. *China Medical Guide*, 11(26), 235-236.
[38] Zou, M. (2017). Efficacy and impact on blood lipids and inflammatory factors of lipid-lowering capsule combined with atorvastatin in the treatment of hyperlipidemia. *World Traditional Chinese Medicine*, 12(8), 1824-1827.
[39] Ye, J. G. (2021). Clinical efficacy of lipid-lowering capsule combined with atorvastatin in the treatment of hyperlipidemia. 2021.
[40] Yu, Q. (2021). Analysis of the application effect of lipid-lowering capsule combined with atorvastatin in the treatment of hyperlipidemia. *Integrated Traditional Chinese and Western Medicine on Cardiovascular and Cerebrovascular Diseases (Electronic Edition)*, 9(33).
[41] Shi, C., Wang, X., & Zhu, H. L. (2018). Clinical observation of lipid-lowering capsule combined with fluvastatin sodium in the treatment of hyperlipidemia. *World Clinical Medicine*, 39(1), 43-46.
[42] Tian, L. (2022). Clinical observation of lipid-lowering capsule combined with pitavastatin in the treatment of coronary heart disease (CHD) with hyperlipidemia. *Chinese Science and Technology Journal Database (Abstract Edition) Medical and Health*, 2022(8), 3.
[43] Qu, J., Ren, Y. Y., & Zhang, X. Y. (2020). The effect and impact on blood lipid metabolism and cardiac function of lipid-lowering capsule combined with pitavastatin in the treatment of coronary heart disease with hyperlipidemia. *Clinical Medical Research and Practice*, 2020.
[44] Xu, B. T., Liu, X. H., Li, Y. M., et al. (2023). Clinical efficacy observation of lipid-lowering capsule combined with rosuvastatin calcium tablets in the treatment of primary hyperlipidemia. 2023, 7.
[45] Sun, X. J., Liu, X. Y., & He, S. M. (2018). The lipid-lowering effect and impact on 24-hour urinary albumin excretion and plasma endothelin-1 level of lipid-lowering capsule combined with simvastatin. *Drug Evaluation Research*, 41(6), 4.
[46] Jiang, C. H., & Chen, Y. Q. (2012). Clinical efficacy observation of lipid-lowering capsule combined with simvastatin in the treatment of hyperlipidemia. *Straits Pharmaceutical,* 24(11), 2.
[47] Huang, C. T., & Huang, H. (2014). Clinical observation of the treatment of hyperlipidemia by integrated traditional Chinese and Western medicine. Medical Information: *Medical and Computer Applications*, 2014(35), 2.
[48] Zhou, Z. (2010). Clinical observation of 39 cases of hyperlipidemia treated by integrated traditional Chinese and Western medicine. *Traditional Chinese Medicine Guide*, 16(4), 17-19.

[49] Tian, L. (2015). Clinical analysis of 78 cases of hyperlipidemia treated by integrated traditional Chinese and Western medicine. *Chinese Clinical Research*, 7(2), 94-95.
[50] Wei, G. R., Huang, Y. C., & Chang, L. P. (2011). Clinical observation of 38 cases of elderly hyperlipidemia treated by integrated traditional Chinese and Western medicine. *Primary Medical Forum*, 1, 73-75.
[51] Chen, H. Y. (2014). Clinical observation of 42 cases of elderly hyperlipidemia treated by integrated traditional Chinese and Western medicine. *Integrated Traditional Chinese and Western Medicine on Cardiovascular and Cerebrovascular Diseases (Electronic Edition)*, 4, 27-28.
[52] Liu, Y. S. (2022). The impact of lipid-lowering capsule combined with atorvastatin on the lipid-lowering effect and cardiovascular events in patients with coronary artery atherosclerotic heart disease and hyperlipidemia. *China Drug Economics*, 17(9), 4.
[53] Hu, G. Y. (2015). Clinical efficacy observation of lipid-lowering capsule combined with atorvastatin in the treatment of hyperlipidemia. *China Primary Health Care*, 6, 3.
[54] Wang, Z. (2012). Clinical efficacy observation of lipid-lowering capsule combined with atorvastatin in the treatment of diabetic hyperlipidemia. *Chinese and Foreign Medical Research*, 10(21), 2.
[55] Jiang, F. (2013). Efficacy observation of lipid-lowering capsule combined with simvastatin in the treatment of elderly hyperlipidemia. *China Medical Guide*, 11(3), 2.
[56] Ma, J. B. (2015). Efficacy observation of lipid-lowering capsule combined with simvastatin in the treatment of coronary heart disease with blood lipid disorders. *Contemporary Medicine*, 015, 133-134.
[57] Li, R. (2023). The efficacy analysis of statins alone and in combination with Xinshou Ketai in the treatment of hyperlipidemia. *World Latest Medical Information*, 63-66.
[58] Zhou, L. (2024). Effects of Xinshou Ketai capsule combined with atorvastatin calcium tablets on coronary heart disease with hyperlipidemia and its impact on lipid metabolism and cardiac function. *Modern Medicine and Health Research*, 8(1), 20-25.
[59] Fu, Y. J. (2017). Observation of the effect of Xinshou Ketai capsule combined with atorvastatin calcium on hyperlipidemia. *Journal of Traditional Chinese Medicine*, 57(8), 782-786.
[60] Xu, X. C. (2024). The efficacy of Xinshou Ketai capsule combined with atorvastatin in the treatment of patients with hyperlipidemia. *Chinese Journal of Integrated Traditional and Western Medicine*, 34(3), 204-210.
[61] Qiang, S. C. (2023). Evaluation of the impact of Xinshou Ketai capsule combined with rosuvastatin on blood lipids and inflammatory factors in patients with hyperlipidemia. *Chinese Journal of Clinical Pharmacology*, 39(5), 431-435.
[62] Shi, W. H. (2011). Clinical treatment analysis of hyperlipidemia. *China Medical Guide*, 9(14), 301-302.
[63] Zhou, H. L. (2015). Efficacy observation of Xue Zhi Tong capsule combined with atorvastatin in the treatment of patients with hyperlipidemia. *Cardiovascular Disease Prevention and Control Knowledge: Academic Edition*, 3, 68-69.
[64] Chen, Q., Ma, X., Gao, F., et al. (2022). The impact of Xue Zhi Tong capsule combined with atorvastatin calcium on patients with peritoneal dialysis and hyperlipidemia. *Journal of Chengdu Medical College*, 17(5), 5.
[65] Yang, D. M., He, H. W., & Lu, Z. F. (2014). Efficacy observation of Xue Zhi Tong capsule combined with atorvastatin calcium in the treatment of elderly patients with hyperlipidemia. *People's Military Doctor*, 57(4), 2.
[66] Xiong, G., & Wang, L. Z. (2019). Efficacy observation of Xue Zhi Tong capsule combined with atorvastatin in the treatment of hyperlipidemia with fatty liver. *Journal of Integrated Traditional and Western Medicine on Cardiovascular and Cerebrovascular Diseases*, 17(2), 3.
[67] Kuang, Z. B. (2010). Efficacy observation of Xue Zhi Tong capsule combined with atorvastatin in the treatment of hyperlipidemia. *China Medical Guide*, 8(35), 2.

S6. Traffic plot of bias risk assessment.

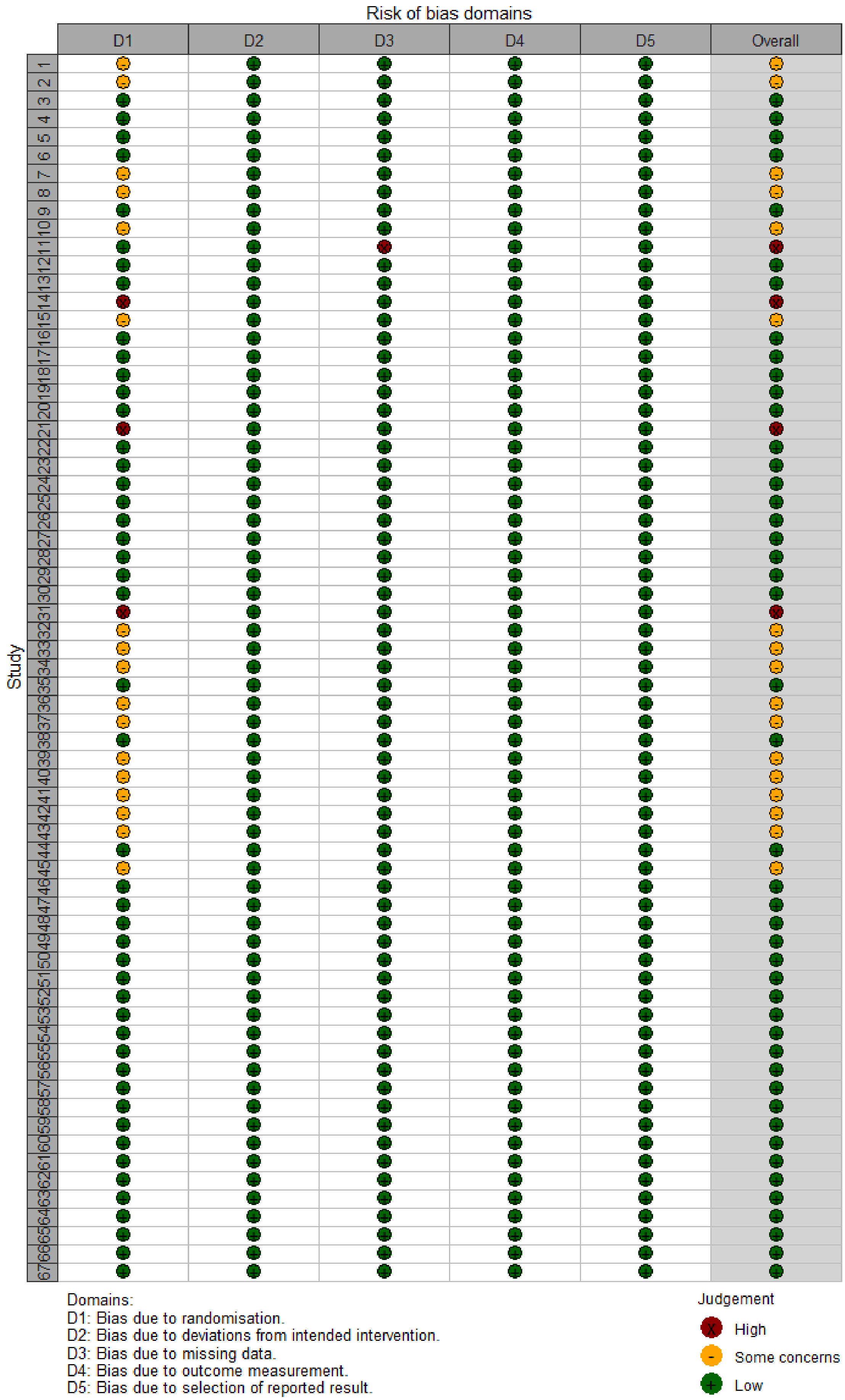

S7. Forest plot based on the difference in intervention measures

S7.1 Clinical total effective rate forest plot.

Study or Subgroup	Experimental Events	Experimental Total	Control Events	Control Total	Weight	Odds Ratio M-H, Fixed, 95% CI
**1.2.1 TT+DTJZ**						
Kuang DD 2019	45	48	36	48	1.6%	5.00 [1.31, 19.07]
Liang ZM 2015	53	56	46	56	1.7%	3.84 [1.00, 14.80]
Lv WH 2013	145	158	129	158	7.4%	2.51 [1.25, 5.03]
Sun JY 2012	43	45	40	45	1.2%	2.69 [0.49, 14.64]
**Subtotal (95% CI)**		**307**		**307**	**11.9%**	**3.05 [1.79, 5.17]**
Total events	286		251			
Heterogeneity: Chi² = 0.96, df = 3 (P = 0.81); I² = 0%						
Test for overall effect: Z = 4.12 (P < 0.0001)						
**1.2.2 TT+JZL**						
Guo XP 2010	33	34	29	34	0.6%	5.69 [0.63, 51.57]
Zhu TW 2021	38	40	32	40	1.1%	4.75 [0.94, 23.98]
**Subtotal (95% CI)**		**74**		**74**	**1.7%**	**5.08 [1.38, 18.71]**
Total events	71		61			
Heterogeneity: Chi² = 0.02, df = 1 (P = 0.90); I² = 0%						
Test for overall effect: Z = 2.44 (P = 0.01)						
**1.2.3 TT+JZTL**						
Bao FY 2009	51	56	39	56	2.4%	4.45 [1.51, 13.10]
Wen CT 2013	52	55	45	55	1.7%	3.85 [1.00, 14.87]
**Subtotal (95% CI)**		**111**		**111**	**4.1%**	**4.20 [1.81, 9.77]**
Total events	103		84			
Heterogeneity: Chi² = 0.03, df = 1 (P = 0.87); I² = 0%						
Test for overall effect: Z = 3.33 (P = 0.0009)						
**1.2.4 TT+JZTM**						
Lu HM 2021	43	46	35	46	1.6%	4.50 [1.17, 17.42]
Xiao B 2014	36	40	27	40	1.9%	4.33 [1.27, 14.78]
You HS 2022	50	50	44	50	0.3%	14.75 [0.81, 269.34]
**Subtotal (95% CI)**		**136**		**136**	**3.8%**	**5.24 [2.22, 12.37]**
Total events	129		106			
Heterogeneity: Chi² = 0.63, df = 2 (P = 0.73); I² = 0%						
Test for overall effect: Z = 3.79 (P = 0.0002)						
**1.2.5 TT+PS**						
Cao CY 2014	38	42	35	42	2.3%	1.90 [0.51, 7.05]
Cao XC 2021	29	32	22	32	1.4%	4.39 [1.08, 17.89]
Gu SM 2015	24	30	18	30	2.5%	2.67 [0.84, 8.46]
Li X 2016	28	30	24	46	0.9%	12.83 [2.73, 60.27]
Mu M 2014	39	43	37	43	2.4%	1.58 [0.41, 6.05]
Wu NJ 2020	37	40	31	39	1.6%	3.18 [0.78, 13.04]
**Subtotal (95% CI)**		**217**		**232**	**11.1%**	**3.37 [1.98, 5.73]**
Total events	195		167			
Heterogeneity: Chi² = 5.13, df = 5 (P = 0.40); I² = 2%						
Test for overall effect: Z = 4.49 (P < 0.00001)						
**1.2.6 TT+SLXMK**						
Cai ZQ 2015	41	48	40	48	4.1%	1.17 [0.39, 3.53]
Feng ZP 2013	285	300	254	300	8.8%	3.44 [1.88, 6.31]
Li HL 2011	50	63	40	57	6.0%	1.63 [0.71, 3.76]
Ning Y 2012	49	50	36	50	0.5%	19.06 [2.40, 151.60]
Zhou P 2012	33	35	18	35	0.7%	15.58 [3.23, 75.18]
**Subtotal (95% CI)**		**496**		**490**	**20.1%**	**3.26 [2.19, 4.87]**
Total events	458		388			
Heterogeneity: Chi² = 12.55, df = 4 (P = 0.01); I² = 68%						
Test for overall effect: Z = 5.79 (P < 0.00001)						
**1.2.7 TT+XZK**						
Chen HY 2014	40	42	31	42	1.0%	7.10 [1.46, 34.38]
Chen Y 2019	29	30	24	30	0.6%	7.25 [0.82, 64.46]
Fu YJ 2017	70	75	61	75	2.8%	3.21 [1.09, 9.44]
Hu GY 2015	45	60	34	60	5.9%	2.29 [1.06, 4.98]
Jiang CH 2012	81	85	70	85	2.3%	4.34 [1.38, 13.68]
Jiang F 2013	26	28	23	28	1.1%	2.83 [0.50, 15.99]
Li RG 2023	97	100	90	100	1.9%	3.59 [0.96, 13.47]
Qu J 2020	37	39	31	39	1.1%	4.77 [0.94, 24.16]
Shi C 2018	53	62	43	62	4.3%	2.60 [1.07, 6.33]
Sun XJ 2018	45	48	37	48	1.6%	4.46 [1.16, 17.18]
Tian L 2015	74	78	57	72	2.1%	4.87 [1.53, 15.46]
Tian L 2022	28	30	22	30	1.0%	5.09 [0.98, 26.43]
Wang Z 2012	37	39	30	39	1.1%	5.55 [1.11, 27.66]
Wang ZZ 2013	25	27	21	27	1.1%	3.57 [0.65, 19.59]
Xu BT 2021	47	50	36	50	1.5%	6.09 [1.63, 22.82]
Xu XC 2024	48	50	42	50	1.2%	4.57 [0.92, 22.73]
Ye JG 2021	58	60	51	60	1.2%	5.12 [1.06, 24.79]
Zhou L 2024	38	41	31	41	1.6%	4.09 [1.03, 16.15]
Zhou M 2017	233	244	203	244	6.4%	4.28 [2.14, 8.54]
Zhou Z 2010	37	39	31	39	1.1%	4.77 [0.94, 24.16]
**Subtotal (95% CI)**		**1227**		**1221**	**40.8%**	**3.97 [3.03, 5.22]**
Total events	1148		968			
Heterogeneity: Chi² = 5.04, df = 19 (P = 1.00); I² = 0%						
Test for overall effect: Z = 9.92 (P < 0.00001)						
**1.2.8 TT+XZT**						
Chen Q 2022	28	30	21	30	1.0%	6.00 [1.17, 30.72]
Xiong G 2019	48	50	43	50	1.2%	3.91 [0.77, 19.83]
Yang DM 2014	36	40	31	40	2.2%	2.61 [0.73, 9.32]
Zhou HL 2015	46	50	39	50	2.2%	3.24 [0.96, 11.00]
**Subtotal (95% CI)**		**170**		**170**	**6.5%**	**3.57 [1.78, 7.15]**
Total events	158		134			
Heterogeneity: Chi² = 0.65, df = 3 (P = 0.88); I² = 0%						
Test for overall effect: Z = 3.59 (P = 0.0003)						
**Total (95% CI)**		**2738**		**2741**	**100.0%**	**3.70 [3.11, 4.42]**
Total events	2548		2159			
Heterogeneity: Chi² = 27.68, df = 45 (P = 0.98); I² = 0%						
Test for overall effect: Z = 14.59 (P < 0.00001)						
Test for subgroup differences: Chi² = 2.23, df = 7 (P = 0.95), I² = 0%						

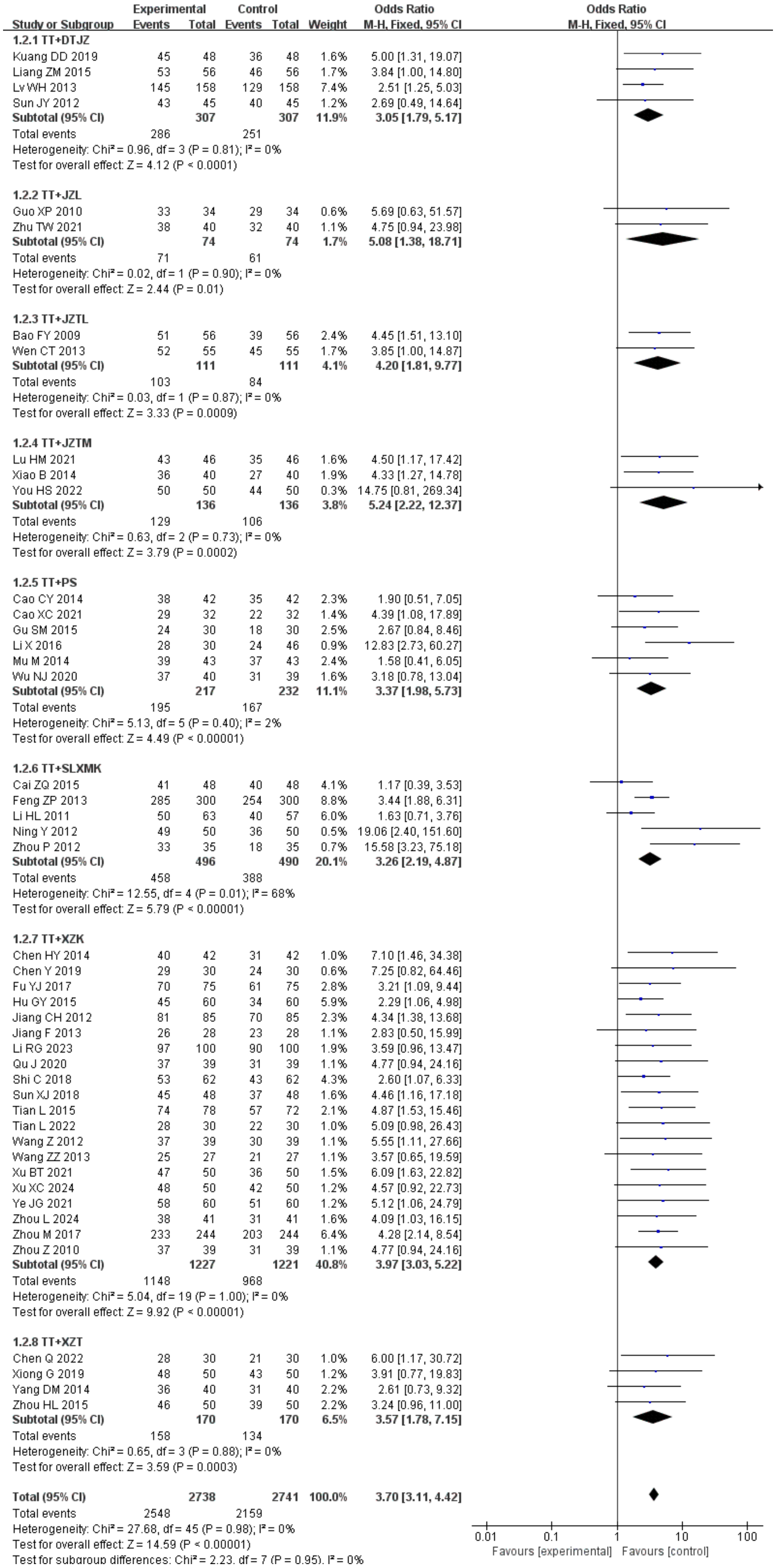

S7.2 TC forest plot.

Study or Subgroup	Experimental Mean	Experimental SD	Experimental Total	Control Mean	Control SD	Control Total	Weight	Mean Difference IV, Random, 95% CI
**2.2.2 TT+DTJZ**								
Kuang DD 2019	-1.95	0.7731106	48	-0.77	0.76019734	48	1.8%	-1.18 [-1.49, -0.87]
Liang ZM 2015	-2.1	0.55344376	56	-1.43	0.53563047	56	1.9%	-0.67 [-0.87, -0.47]
Lv WH 2013	-3.01	1.28035151	158	-1.29	1.41265707	158	1.8%	-1.72 [-2.02, -1.42]
Sun JY 2012	-1.53	0.81871851	45	-0.96	0.73013697	45	1.8%	-0.57 [-0.89, -0.25]
Wang QC 2012	-3.25	0.80018748	30	-2.67	0.77659513	30	1.6%	-0.58 [-0.98, -0.18]
**Subtotal (95% CI)**			**337**			**337**	**8.9%**	**-0.95 [-1.38, -0.51]**
Heterogeneity: Tau² = 0.22; Chi² = 43.85, df = 4 (P < 0.00001); I² = 91%								
Test for overall effect: Z = 4.27 (P < 0.0001)								
**2.2.3 TT+JZL**								
Guo XP 2010	-2.33	1.57645171	34	-0.96	1.59144588	34	1.1%	-1.37 [-2.12, -0.62]
Hu XW 2015	-1.39	1.02532922	48	-0.89	1.05910339	50	1.6%	-0.50 [-0.91, -0.09]
Zhang XL 2017	-1.7	0.81902381	50	-1.02	0.79730797	50	1.8%	-0.68 [-1.00, -0.36]
Zhu TW 2021	-2.07	0.93375586	40	-1.28	0.96145723	40	1.6%	-0.79 [-1.21, -0.37]
**Subtotal (95% CI)**			**172**			**174**	**6.1%**	**-0.73 [-0.98, -0.48]**
Heterogeneity: Tau² = 0.02; Chi² = 4.12, df = 3 (P = 0.25); I² = 27%								
Test for overall effect: Z = 5.68 (P < 0.00001)								
**2.2.4 TT+JZTL**								
Bao FY 2009	-2.95	2.09148273	56	-2.08	2.27118912	56	1.0%	-0.87 [-1.68, -0.06]
Guo YW 2017	-2.53	1.04	58	-1.84	1.0359054	58	1.7%	-0.69 [-1.07, -0.31]
Wen CT 2013	-1.8	1.30984732	55	-1.23	1.32011363	55	1.5%	-0.57 [-1.06, -0.08]
Xie Y 2014	-1.72	0.9606248	60	-1.61	0.82655913	62	1.8%	-0.11 [-0.43, 0.21]
**Subtotal (95% CI)**			**229**			**231**	**5.9%**	**-0.49 [-0.85, -0.14]**
Heterogeneity: Tau² = 0.07; Chi² = 7.15, df = 3 (P = 0.07); I² = 58%								
Test for overall effect: Z = 2.75 (P = 0.006)								
**2.2.5 TT+JZTM**								
Liu FG 2011	-1.42	1.08871484	30	-1.42	1.04417432	30	1.4%	0.00 [-0.54, 0.54]
Lu HM 2021	-2.51	0.72746134	46	-1.73	0.73321211	46	1.8%	-0.78 [-1.08, -0.48]
Wang H 2019	-1.43	0.84929382	15	-0.73	1.06887792	15	1.2%	-0.70 [-1.39, -0.01]
Xiao B 2014	-2.25	0.52564246	40	-1.26	0.49517674	40	1.9%	-0.99 [-1.21, -0.77]
You HS 2022	-2.73	0.61830413	50	-2.07	0.72380937	50	1.9%	-0.66 [-0.92, -0.40]
**Subtotal (95% CI)**			**181**			**181**	**8.1%**	**-0.69 [-0.96, -0.41]**
Heterogeneity: Tau² = 0.06; Chi² = 12.24, df = 4 (P = 0.02); I² = 67%								
Test for overall effect: Z = 4.95 (P < 0.00001)								
**2.2.6 TT+PS**								
Cao CY 2014	-1.75	0.76393717	42	-1.61	0.74343796	42	1.8%	-0.14 [-0.46, 0.18]
Cao XC 2021	-1.66	0.71463277	32	-1.72	0.94694245	32	1.6%	0.06 [-0.35, 0.47]
Chen X 2016	-0.78	0.16370706	20	-0.63	0.11789826	20	2.0%	-0.15 [-0.24, -0.06]
Gu SM 2015	-1.66	0.73184698	30	-1.35	0.74719475	30	1.7%	-0.31 [-0.68, 0.06]
Li X 2016	-2.63	1.17460632	30	-2.18	1.17204949	46	1.4%	-0.45 [-0.99, 0.09]
Mu M 2014	-1.38	1.08448144	43	-1.31	0.99579114	43	1.6%	-0.07 [-0.51, 0.37]
Shao ZX 2016	-0.88	1.31730786	70	-1.09	1.18646534	35	1.5%	0.21 [-0.29, 0.71]
Wu NJ 2020	-1.83	1.03793063	40	-1.1	1.6214808	39	1.3%	-0.73 [-1.33, -0.13]
Yu GL 2013	-1.73	1.16116321	40	-1.76	1.35723985	40	1.4%	0.03 [-0.52, 0.58]
Zhang SY 2013	-1.6	0.76151166	30	-1.59	0.80205985	30	1.6%	-0.01 [-0.41, 0.39]
**Subtotal (95% CI)**			**377**			**357**	**15.9%**	**-0.14 [-0.23, -0.05]**
Heterogeneity: Tau² = 0.00; Chi² = 9.45, df = 9 (P = 0.40); I² = 5%								
Test for overall effect: Z = 3.08 (P = 0.002)								
**2.2.7 TT+SLXMK**								
Cai ZQ 2015	-2.06	1.09995454	48	-1.18	1.07698654	48	1.6%	-0.88 [-1.32, -0.44]
Li HL 2011	-0.86	2.27501648	63	-0.93	2.24626356	57	1.0%	0.07 [-0.74, 0.88]
Ning Y 2012	-3.6	1.15325626	50	-2.39	1.15325626	50	1.6%	-1.21 [-1.66, -0.76]
Zhou Y 2017	-2.37	1.13767306	41	-1.61	1.2061509	39	1.4%	-0.76 [-1.27, -0.25]
**Subtotal (95% CI)**			**202**			**194**	**5.6%**	**-0.79 [-1.20, -0.37]**
Heterogeneity: Tau² = 0.11; Chi² = 7.52, df = 3 (P = 0.06); I² = 60%								
Test for overall effect: Z = 3.69 (P = 0.0002)								
**2.2.8 TT+XZK**								
Chen HY 2014	-3.91	0.61506097	42	-3.5	0.55018179	42	1.9%	-0.41 [-0.66, -0.16]
Fu YJ 2017	-2	0.87177979	75	-1.2	0.9539392	75	1.8%	-0.80 [-1.09, -0.51]
Gui YF 2019	-2.21	1.00881118	36	-1.55	1.02586549	36	1.5%	-0.66 [-1.13, -0.19]
Hu GY 2015	-2	1.12343224	60	-1.43	1.06719258	60	1.7%	-0.57 [-0.96, -0.18]
Huang CC 2014	-2.37	0.99050492	30	-2.4	0.98305646	25	1.4%	0.03 [-0.49, 0.55]
Jiang CH 2012	-0.83	0.55380502	85	-0.47	0.54990908	85	2.0%	-0.36 [-0.53, -0.19]
Jiang F 2013	-1.72	0.49122296	28	-1.44	0.57662813	28	1.8%	-0.28 [-0.56, 0.00]
Li RG 2023	-1.52	1.5400974	100	-0.66	1.44564864	100	1.6%	-0.86 [-1.27, -0.45]
Liu YS 2022	-2.25	1.06296754	59	-1.93	1.04919016	59	1.7%	-0.32 [-0.70, 0.06]
Ma JB 2015	-1.06	0.68439754	40	-1.16	0.57887823	40	1.8%	0.10 [-0.18, 0.38]
Qiang SC 2023	-1.78	0.55434646	90	-1.08	0.59	90	2.0%	-0.70 [-0.87, -0.53]
Qu J 2020	-1.47	1.21024791	39	-0.98	1.30219046	39	1.4%	-0.49 [-1.05, 0.07]
Shi C 2018	-1.96	0.80074965	62	-1.33	0.79699435	62	1.8%	-0.63 [-0.91, -0.35]
Sun XJ 2018	-1.46	0.55569776	48	-0.59	0.55973208	48	1.9%	-0.87 [-1.09, -0.65]
Tian L 2022	-1.94	0.81504601	30	-0.84	0.77948701	30	1.6%	-1.10 [-1.50, -0.70]
Wang Z 2012	-2.02	0.94556861	39	-1.91	0.7014984	39	1.7%	-0.11 [-0.48, 0.26]
Wang ZZ 2013	-2.87	0.68789534	27	-1.05	0.71042241	27	1.7%	-1.82 [-2.19, -1.45]
Wei GR 2011	-1.691	0.69733995	38	-1.514	0.43675622	38	1.9%	-0.18 [-0.44, 0.08]
Xu XC 2024	-3.06	0.67089492	50	-1.53	0.62385896	50	1.9%	-1.53 [-1.78, -1.28]
Ye JG 2021	-3	1.73493516	60	-2.3	1.85202592	60	1.2%	-0.70 [-1.34, -0.06]
Yu Q 2021	-1.55	1.15060854	38	-0.68	1.21786699	38	1.4%	-0.87 [-1.40, -0.34]
Zhou L 2024	-1.54	0.9477869	41	-1.03	0.94694245	41	1.6%	-0.51 [-0.92, -0.10]
Zhou M 2017	-1.51	1.52502459	244	-0.67	1.46563979	244	1.8%	-0.84 [-1.11, -0.57]
Zhou Z 2010	-2.96	0.79542442	39	-2.99	0.59354865	39	1.8%	0.03 [-0.28, 0.34]
**Subtotal (95% CI)**			**1400**			**1395**	**40.9%**	**-0.60 [-0.78, -0.42]**
Heterogeneity: Tau² = 0.17; Chi² = 193.25, df = 23 (P < 0.00001); I² = 88%								
Test for overall effect: Z = 6.49 (P < 0.00001)								
**2.2.9 TT+XZT**								
Chen Q 2022	-1.76	0.95566731	30	-1	0.80721744	30	1.6%	-0.76 [-1.21, -0.31]
Kuang ZB 2010	-2.14	1.27	60	-2.12	0.88898819	60	1.7%	-0.02 [-0.41, 0.37]
Shi WH 2011	-1.73	3.12635571	60	-1.15	2.20469953	60	0.8%	-0.58 [-1.55, 0.39]
Xiong G 2019	-3.72	0.46518813	50	-2.96	0.4927474	50	1.9%	-0.76 [-0.95, -0.57]
Yang DM 2014	-2.33	1.95138412	40	-1.16	1.40758659	40	1.1%	-1.17 [-1.92, -0.42]
Zhou HL 2015	-2.16	1.26003968	50	-1.1	1.1931471	50	1.5%	-1.06 [-1.54, -0.58]
**Subtotal (95% CI)**			**290**			**290**	**8.6%**	**-0.70 [-1.02, -0.37]**
Heterogeneity: Tau² = 0.10; Chi² = 15.74, df = 5 (P = 0.008); I² = 68%								
Test for overall effect: Z = 4.19 (P < 0.0001)								
**Total (95% CI)**			**3188**			**3159**	**100.0%**	**-0.59 [-0.70, -0.48]**
Heterogeneity: Tau² = 0.16; Chi² = 461.51, df = 61 (P < 0.00001); I² = 87%								
Test for overall effect: Z = 10.15 (P < 0.00001)								
Test for subgroup differences: Chi² = 58.15, df = 7 (P < 0.00001), I² = 88.0%								

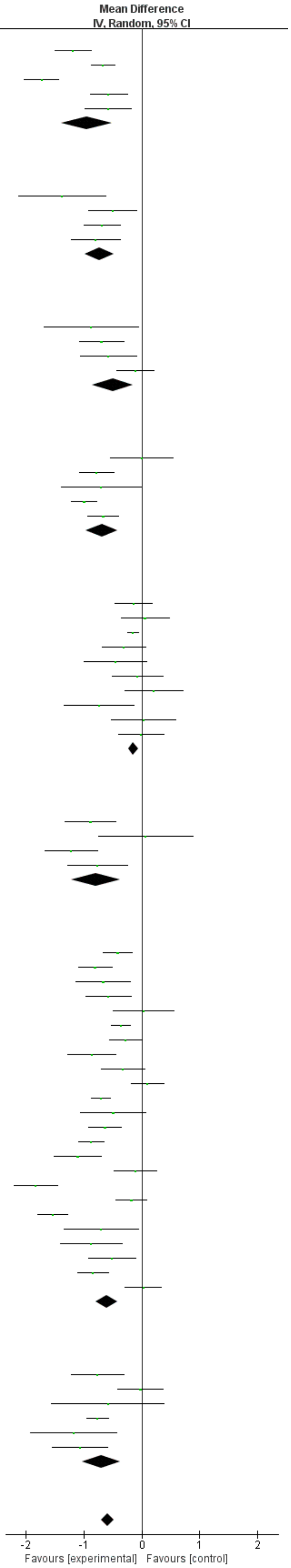

S7.3 TG forest plot.

Study or Subgroup	Experimental Mean	Experimental SD	Experimental Total	Control Mean	Control SD	Control Total	Weight	Mean Difference IV, Random, 95% CI
**3.1.1 TT+DTJZ**								
Kuang DD 2019	-1.23	0.63788714	48	-0.52	0.67734777	48	1.9%	-0.71 [-0.97, -0.45]
Liang ZM 2015	-1.34	0.48877398	56	-0.84	0.49426713	56	2.2%	-0.50 [-0.68, -0.32]
Lv WH 2013	-1.03	0.77826731	158	-0.37	0.77672389	158	2.2%	-0.66 [-0.83, -0.49]
Sun JY 2012	-1	0.23065125	45	-0.73	0.29614186	45	2.4%	-0.27 [-0.38, -0.16]
Wang QC 2012	-1.46	1.04541858	30	-1.24	0.87846457	30	1.2%	-0.22 [-0.71, 0.27]
**Subtotal (95% CI)**			**337**			**337**	**9.8%**	**-0.49 [-0.69, -0.28]**
Heterogeneity: Tau² = 0.04; Chi² = 20.71, df = 4 (P = 0.0004); I² = 81%								
Test for overall effect: Z = 4.70 (P < 0.00001)								
**3.1.2 TT+JZL**								
Guo XP 2010	-1.48	0.75940766	34	-1.38	0.78083289	34	1.5%	-0.10 [-0.47, 0.27]
Hu XW 2015	-0.82	1.7351657	48	-0.3	1.72217885	50	0.8%	-0.52 [-1.20, 0.16]
Zhang XL 2017	-1.27	0.61587336	50	-0.57	0.66550732	50	1.9%	-0.70 [-0.95, -0.45]
Zhu TW 2021	-1.49	0.62193247	40	-0.93	0.69346954	40	1.8%	-0.56 [-0.85, -0.27]
**Subtotal (95% CI)**			**172**			**174**	**6.0%**	**-0.49 [-0.76, -0.21]**
Heterogeneity: Tau² = 0.04; Chi² = 7.10, df = 3 (P = 0.07); I² = 58%								
Test for overall effect: Z = 3.50 (P = 0.0005)								
**3.1.3 TT+JZTL**								
Bao FY 2009	-1.07	1.26526677	56	-0.75	0.8141253	56	1.4%	-0.32 [-0.71, 0.07]
Guo YW 2017	-1.26	0.89213228	58	-0.53	1.01680873	58	1.6%	-0.73 [-1.08, -0.38]
Wen CT 2013	-1.03	1.04129727	55	-0.7	1.05484596	55	1.4%	-0.33 [-0.72, 0.06]
Xie Y 2014	-1.26	1.26348724	60	-0.73	1.54476535	62	1.1%	-0.53 [-1.03, -0.03]
**Subtotal (95% CI)**			**229**			**231**	**5.6%**	**-0.49 [-0.70, -0.28]**
Heterogeneity: Tau² = 0.00; Chi² = 3.21, df = 3 (P = 0.36); I² = 6%								
Test for overall effect: Z = 4.64 (P < 0.00001)								
**3.1.4 TT+JZTM**								
Liu FG 2011	-0.43	0.95582425	30	-0.25	1.27432335	30	1.0%	-0.18 [-0.75, 0.39]
Lu HM 2021	-1.28	1.54618886	46	-0.42	1.56144164	46	0.9%	-0.86 [-1.50, -0.22]
Wang H 2019	-0.97	0.96994845	15	-0.4	0.90537285	15	0.8%	-0.57 [-1.24, 0.10]
Xiao B 2014	-1.16	0.53674948	40	-1.05	0.46936127	40	2.0%	-0.11 [-0.33, 0.11]
You HS 2022	-1.27	1.27996094	50	-0.38	1.23121891	50	1.2%	-0.89 [-1.38, -0.40]
**Subtotal (95% CI)**			**181**			**181**	**5.8%**	**-0.48 [-0.86, -0.10]**
Heterogeneity: Tau² = 0.12; Chi² = 12.13, df = 4 (P = 0.02); I² = 67%								
Test for overall effect: Z = 2.51 (P = 0.01)								
**3.1.5 TT+PS**								
Cao CY 2014	-2.31	1.10891839	42	-1.97	1.00642933	42	1.3%	-0.34 [-0.79, 0.11]
Cao XC 2021	-2.29	1.3716049	32	-1.83	1.31053424	32	0.8%	-0.46 [-1.12, 0.20]
Chen X 2016	-1.4	0.3508561	20	-0.79	0.34394767	20	2.1%	-0.61 [-0.83, -0.39]
Gu SM 2015	-1.11	0.8908423	30	-0.92	0.9367497	30	1.2%	-0.19 [-0.65, 0.27]
Li X 2016	-1.43	0.77543536	30	-1.33	0.8107404	46	1.5%	-0.10 [-0.46, 0.26]
Mu M 2014	-2.19	0.78619336	43	-1.31	1.4650256	43	1.1%	-0.88 [-1.38, -0.38]
Shao ZX 2016	-2.38	0.67357256	70	-1.29	0.74054034	35	1.8%	-1.09 [-1.38, -0.80]
Wu NJ 2020	-1.63	1.6021548	40	-1.12	2.10256986	39	0.6%	-0.51 [-1.34, 0.32]
Yu GL 2013	-1.07	1.26099167	40	-0.37	0.83719771	40	1.2%	-0.70 [-1.17, -0.23]
Zhang SY 2013	-2.33	1.11498879	30	-1.41	1.12769677	30	1.0%	-0.92 [-1.49, -0.35]
**Subtotal (95% CI)**			**377**			**357**	**12.6%**	**-0.59 [-0.81, -0.36]**
Heterogeneity: Tau² = 0.08; Chi² = 25.18, df = 9 (P = 0.003); I² = 64%								
Test for overall effect: Z = 5.10 (P < 0.00001)								
**3.1.6 TT+SLXMK**								
Cai ZQ 2015	-0.95	0.90812995	48	-0.71	0.55973208	48	1.8%	-0.24 [-0.54, 0.06]
Li HL 2011	-0.68	1.04990476	63	-0.39	0.84445248	57	1.6%	-0.29 [-0.63, 0.05]
Ning Y 2012	-0.9	0.72505172	50	-0.12	0.75504967	50	1.8%	-0.78 [-1.07, -0.49]
Zhou Y 2017	-0.61	0.86954011	41	-0.48	0.52716221	39	1.7%	-0.13 [-0.44, 0.18]
**Subtotal (95% CI)**			**202**			**194**	**6.9%**	**-0.36 [-0.66, -0.07]**
Heterogeneity: Tau² = 0.07; Chi² = 10.84, df = 3 (P = 0.01); I² = 72%								
Test for overall effect: Z = 2.42 (P = 0.02)								
**3.1.7 TT+XZK**								
Chen HY 2014	-0.99	0.33867388	42	-0.68	0.35538711	42	2.3%	-0.31 [-0.46, -0.16]
Fu YJ 2017	-0.5	0.34641016	75	-0.3	0.4	75	2.4%	-0.20 [-0.32, -0.08]
Gui YF 2019	-0.76	0.83372657	36	-0.37	0.54616847	36	1.7%	-0.39 [-0.72, -0.06]
Hu GY 2015	-1.09	1.08087927	60	-0.89	0.95268043	60	1.5%	-0.20 [-0.56, 0.16]
Huang CC 2014	-0.72	0.77090856	30	-1	0.82310388	25	1.3%	0.28 [-0.14, 0.70]
Jiang CH 2012	-0.69	0.40447497	85	-0.4	0.41146081	85	2.4%	-0.29 [-0.41, -0.17]
Jiang F 2013	-1.39	0.71965269	28	-1.4	0.70448563	28	1.5%	0.01 [-0.36, 0.38]
Li RG 2023	-0.45	0.47507894	100	-0.2	0.50507425	100	2.3%	-0.25 [-0.39, -0.11]
Liu YS 2022	-1.13	0.50209561	59	-0.91	0.62385896	59	2.1%	-0.22 [-0.42, -0.02]
Ma JB 2015	-0.78	0.51215232	40	-0.55	0.61506097	40	1.9%	-0.23 [-0.48, 0.02]
Qiang SC 2023	-0.69	0.22113344	90	-0.37	0.22271058	90	2.5%	-0.32 [-0.38, -0.26]
Shi C 2018	-1.25	0.46227697	62	-0.68	0.45177428	62	2.2%	-0.57 [-0.73, -0.41]
Sun XJ 2018	-0.51	0.33645208	48	-0.28	0.37589892	48	2.3%	-0.23 [-0.37, -0.09]
Tian L 2022	-1.46	0.3207803	30	-0.69	0.32511536	30	2.2%	-0.77 [-0.93, -0.61]
Wang Z 2012	-0.69	0.29715316	39	-0.34	0.41509035	39	2.2%	-0.35 [-0.51, -0.19]
Wang ZZ 2013	-1.42	0.3241913	27	-0.75	0.28213472	27	2.2%	-0.67 [-0.83, -0.51]
Wei GR 2011	-1.938	1.61448072	38	-2.114	1.40982588	38	0.8%	0.18 [-0.51, 0.86]
Xu XC 2024	-1.44	0.2251666	50	-0.51	0.24	50	2.4%	-0.93 [-1.02, -0.84]
Ye JG 2021	-1.3	1.11355287	60	-0.7	0.9539392	60	1.5%	-0.60 [-0.97, -0.23]
Yu Q 2021	-0.43	0.42579338	38	-0.16	0.45508241	38	2.1%	-0.27 [-0.47, -0.07]
Zhou L 2024	-2.68	0.41605288	41	-2.13	0.37040518	41	2.2%	-0.55 [-0.72, -0.38]
Zhou M 2017	-0.43	0.46508064	244	-0.21	0.50119856	244	2.4%	-0.22 [-0.31, -0.13]
Zhou Z 2010	-1.1	0.7005712	39	-1.01	0.78083289	39	1.7%	-0.09 [-0.42, 0.24]
**Subtotal (95% CI)**			**1361**			**1356**	**46.3%**	**-0.34 [-0.46, -0.23]**
Heterogeneity: Tau² = 0.06; Chi² = 244.84, df = 22 (P < 0.00001); I² = 91%								
Test for overall effect: Z = 6.09 (P < 0.00001)								
**3.1.8 TT+XZT**								
Chen Q 2022	-1.52	0.98747152	30	-1.14	0.90796476	30	1.2%	-0.38 [-0.86, 0.10]
Kuang ZB 2010	-1.23	1.2805858	60	-0.98	0.97872366	60	1.4%	-0.25 [-0.66, 0.16]
Shi WH 2011	-1.82	1.20328716	60	-1.34	1.57248211	60	1.1%	-0.48 [-0.98, 0.02]
Xiong G 2019	-1.32	1.2994999	50	-0.83	1.30610107	50	1.1%	-0.49 [-1.00, 0.02]
Yang DM 2014	-1.53	1.39581517	40	-0.65	1.3777155	40	0.9%	-0.88 [-1.49, -0.27]
Zhou HL 2015	-1.23	1.28980619	50	-0.65	1.25407336	50	1.1%	-0.58 [-1.08, -0.08]
**Subtotal (95% CI)**			**290**			**290**	**6.9%**	**-0.47 [-0.67, -0.27]**
Heterogeneity: Tau² = 0.00; Chi² = 3.20, df = 5 (P = 0.67); I² = 0%								
Test for overall effect: Z = 4.58 (P < 0.00001)								
**Total (95% CI)**			**3149**			**3120**	**100.0%**	**-0.42 [-0.50, -0.35]**
Heterogeneity: Tau² = 0.05; Chi² = 344.09, df = 60 (P < 0.00001); I² = 83%								
Test for overall effect: Z = 11.54 (P < 0.00001)								
Test for subgroup differences: Chi² = 5.36, df = 7 (P = 0.62), I² = 0%								

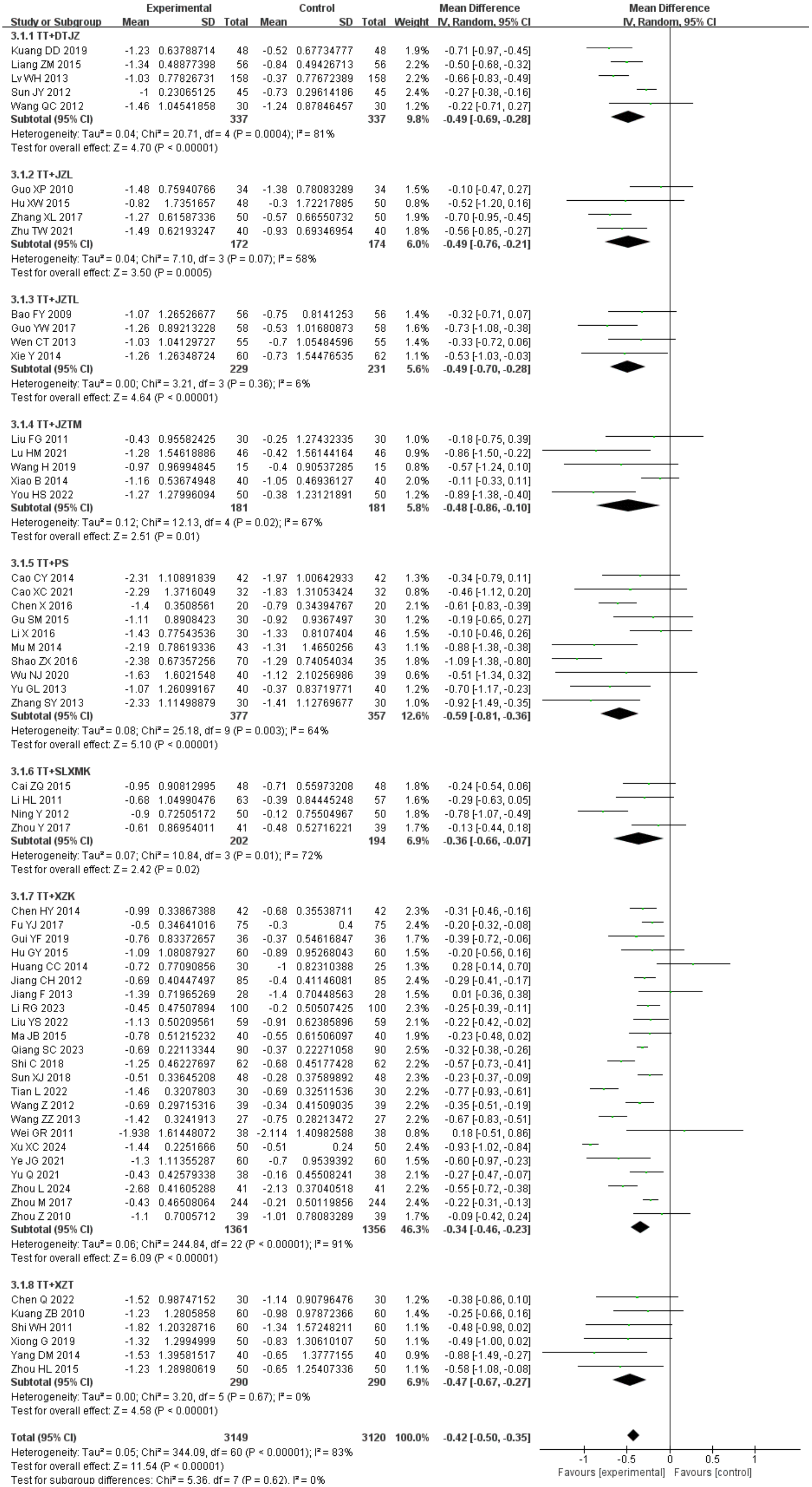

S7.4 HDL-C forest plot.

Study or Subgroup	Experimental Mean	Experimental SD	Experimental Total	Control Mean	Control SD	Control Total	Weight	Mean Difference IV, Random, 95% CI
**4.2.1 TT+DTJZ**								
Wang QC 2012	0.32	0.24062419	30	0.26	0.28583212	30	1.7%	0.06 [-0.07, 0.19]
Sun JY 2012	0.32	0.18734994	45	0.17	0.1473092	45	2.0%	0.15 [0.08, 0.22]
Lv WH 2013	0.69	0.38196858	158	0.32	0.28618176	158	1.9%	0.37 [0.30, 0.44]
Liang ZM 2015	0.52	0.40632499	56	0.15	0.3897435	56	1.7%	0.37 [0.22, 0.52]
Kuang DD 2019	0.92	0.60099917	48	0.07	0.64552304	48	1.2%	0.85 [0.60, 1.10]
**Subtotal (95% CI)**			**337**			**337**	**8.5%**	**0.33 [0.16, 0.51]**
Heterogeneity: Tau² = 0.03; Chi² = 50.19, df = 4 (P < 0.00001); I² = 92%								
Test for overall effect: Z = 3.73 (P = 0.0002)								
**4.2.2 TT+JZL**								
Zhu TW 2021	0.55	0.25632011	40	0.23	0.27838822	40	1.8%	0.32 [0.20, 0.44]
Zhang XL 2017	0.87	0.62553977	50	0.05	0.6819824	50	1.2%	0.82 [0.56, 1.08]
Hu XW 2015	0.36	0.35171011	48	0.19	0.37363083	50	1.7%	0.17 [0.03, 0.31]
Guo XP 2010	0.38	0.33600595	34	0.1	0.37589892	34	1.6%	0.28 [0.11, 0.45]
**Subtotal (95% CI)**			**172**			**174**	**6.3%**	**0.37 [0.17, 0.58]**
Heterogeneity: Tau² = 0.04; Chi² = 18.98, df = 3 (P = 0.0003); I² = 84%								
Test for overall effect: Z = 3.60 (P = 0.0003)								
**4.2.3 TT+JZTL**								
Xie Y 2014	0.06	0.26210685	60	0.03	0.3	62	1.9%	0.03 [-0.07, 0.13]
Wen CT 2013	0.6	0.67089492	55	0.35	0.55973208	55	1.3%	0.25 [0.02, 0.48]
Guo YW 2017	2.03	0.57861905	58	1.75	0.6285698	58	1.4%	0.28 [0.06, 0.50]
Bao FY 2009	0.42	0.76216796	56	-0.13	0.53702886	56	1.3%	0.55 [0.31, 0.79]
**Subtotal (95% CI)**			**229**			**231**	**5.8%**	**0.26 [0.03, 0.49]**
Heterogeneity: Tau² = 0.05; Chi² = 17.90, df = 3 (P = 0.0005); I² = 83%								
Test for overall effect: Z = 2.21 (P = 0.03)								
**4.2.4 TT+JZTM**								
You HS 2022	0.81	0.27784888	50	0.3	0.33600595	50	1.8%	0.51 [0.39, 0.63]
Xiao B 2014	0.26	0.22338308	40	0.21	0.14798649	40	1.9%	0.05 [-0.03, 0.13]
Wang H 2019	1.31	0.7192357	15	0.11	0.91	15	0.4%	1.20 [0.61, 1.79]
Lu HM 2021	0.88	0.27838822	46	0.32	0.27055498	46	1.8%	0.56 [0.45, 0.67]
Liu FG 2011	0.01	0.23895606	30	0.03	0.24062419	30	1.8%	-0.02 [-0.14, 0.10]
**Subtotal (95% CI)**			**181**			**181**	**7.7%**	**0.38 [0.10, 0.67]**
Heterogeneity: Tau² = 0.09; Chi² = 98.43, df = 4 (P < 0.00001); I² = 96%								
Test for overall effect: Z = 2.62 (P = 0.009)								
**4.2.5 TT+PS**								
Zhang SY 2013	1.12	0.25238859	30	0.03	0.34394767	30	1.7%	1.09 [0.94, 1.24]
Yu GL 2013	0.21	0.41581246	40	0.1	0.33511192	40	1.6%	0.11 [-0.06, 0.28]
Wu NJ 2020	0.19	0.25514702	40	0.09	0.50408333	39	1.5%	0.10 [-0.08, 0.28]
Shao ZX 2016	0.06	0.29137605	70	0.04	0.11135529	35	1.9%	0.02 [-0.06, 0.10]
Mu M 2014	0.66	0.25238859	40	0.11	0.32741411	43	1.8%	0.55 [0.43, 0.67]
Li X 2016	0.18	0.38691084	30	0.11	0.49152823	46	1.5%	0.07 [-0.13, 0.27]
Gu SM 2015	0.25	0.62864935	30	0.16	0.21656408	30	1.3%	0.09 [-0.15, 0.33]
Chen X 2016	0.24	0.17088008	20	0.26	0.08544004	20	1.9%	-0.02 [-0.10, 0.06]
Cao XC 2021	1.07	0.16643317	32	0.66	0.10440306	32	2.0%	0.41 [0.34, 0.48]
Cao CY 2014	0.62	0.23065125	42	0.01	0.27838822	42	1.8%	0.61 [0.50, 0.72]
**Subtotal (95% CI)**			**377**			**357**	**16.9%**	**0.31 [0.10, 0.51]**
Heterogeneity: Tau² = 0.10; Chi² = 282.72, df = 9 (P < 0.00001); I² = 97%								
Test for overall effect: Z = 2.93 (P = 0.003)								
**4.2.6 TT+SLXMK**								
Zhou Y 2017	0.61	0.43554564	41	0.36	0.6385139	39	1.3%	0.25 [0.01, 0.49]
Ning Y 2012	0.21	0.56	50	0.04	0.56026779	50	1.4%	0.17 [-0.05, 0.39]
Li HL 2011	1.37	0.90072193	63	0.84	1.03595367	57	0.9%	0.53 [0.18, 0.88]
Cai ZQ 2015	0.29	0.75345869	48	0.32	0.67535176	48	1.1%	-0.03 [-0.32, 0.26]
**Subtotal (95% CI)**			**202**			**194**	**4.6%**	**0.21 [0.02, 0.40]**
Heterogeneity: Tau² = 0.02; Chi² = 6.15, df = 3 (P = 0.10); I² = 51%								
Test for overall effect: Z = 2.17 (P = 0.03)								
**4.2.7 TT+XZK**								
Zhou Z 2010	0.34	0.2116601	39	0.11	0.20952327	39	1.9%	0.23 [0.14, 0.32]
Zhou M 2017	0.48	0.47465777	244	0.2	0.4613025	244	1.9%	0.28 [0.20, 0.36]
Zhou L 2024	0.56	0.45431267	41	0.37	0.40706265	41	1.5%	0.19 [0.00, 0.38]
Yu Q 2021	0.58	0.30805844	38	0.15	0.36373067	38	1.7%	0.43 [0.28, 0.58]
Ye JG 2021	0.5	0.34641016	60	0.3	0.26457513	60	1.8%	0.20 [0.09, 0.31]
Xu XC 2024	0.45	0.52373658	50	0.17	0.33151169	50	1.6%	0.28 [0.11, 0.45]
Wei GR 2011	0.018	0.85378393	38	0.013	0.15418171	38	1.1%	0.00 [-0.27, 0.28]
Wang ZZ 2013	0.51	0.14177447	27	0.16	0.09539392	27	2.0%	0.35 [0.29, 0.41]
Wang Z 2012	0.29	0.13453624	39	0.11	0.12288206	39	2.0%	0.18 [0.12, 0.24]
Tian L 2022	0.86	0.23515952	30	0.4	0.24269322	30	1.8%	0.46 [0.34, 0.58]
Sun XJ 2018	0.11	0.18734994	48	0.04	0.19313208	48	1.9%	0.07 [-0.01, 0.15]
Shi C 2018	0.26	0.34394767	62	0.05	0.27221315	62	1.8%	0.21 [0.10, 0.32]
Qu J 2020	0.6	0.42508823	39	0.34	0.31953091	39	1.6%	0.26 [0.09, 0.43]
Qiang SC 2023	0.31	0.10148892	90	0.13	0.09165151	90	2.0%	0.18 [0.15, 0.21]
Ma JB 2015	0.16	0.22715633	40	0.15	0.24062419	40	1.9%	0.01 [-0.09, 0.11]
Liu YS 2022	0.57	0.16522712	59	0.45	0.19924859	59	2.0%	0.12 [0.05, 0.19]
Li RG 2023	0.45	0.46486557	100	0.18	0.46032597	100	1.8%	0.27 [0.14, 0.40]
Jiang F 2013	0.42	0.30116441	28	0.22	0.30512293	28	1.6%	0.20 [0.04, 0.36]
Jiang CH 2012	0.36	0.15394804	85	0.2	0.14525839	85	2.0%	0.16 [0.12, 0.20]
Hu GY 2015	0.67	0.44170126	60	0.29	0.84184322	60	1.3%	0.38 [0.14, 0.62]
Fu YJ 2017	0.2	0.30512293	75	0.08	0.20518284	75	1.9%	0.12 [0.04, 0.20]
Chen HY 2014	0.4	0.15524175	42	0.29	0.13114877	42	2.0%	0.11 [0.05, 0.17]
**Subtotal (95% CI)**			**1334**			**1334**	**39.0%**	**0.21 [0.17, 0.25]**
Heterogeneity: Tau² = 0.01; Chi² = 102.17, df = 21 (P < 0.00001); I² = 79%								
Test for overall effect: Z = 9.95 (P < 0.00001)								
**4.2.8 TT+XZT**								
Zhou HL 2015	0.46	0.18520259	50	0.11	0.2946184	50	1.9%	0.35 [0.25, 0.45]
Yang DM 2014	0.35	0.22649503	40	0.22	0.16522712	40	1.9%	0.13 [0.04, 0.22]
Xiong G 2019	0.48	0.2007486	50	0.09	0.16522712	50	2.0%	0.39 [0.32, 0.46]
Shi WH 2011	0.33	0.27622455	60	0.19	0.20223748	60	1.9%	0.14 [0.05, 0.23]
Kuang ZB 2010	0.44	0.19467922	60	0.11	0.12529964	60	2.0%	0.33 [0.27, 0.39]
Chen Q 2022	0.51	0.42579338	30	0.21	0.40632499	30	1.4%	0.30 [0.09, 0.51]
**Subtotal (95% CI)**			**290**			**290**	**11.0%**	**0.27 [0.18, 0.37]**
Heterogeneity: Tau² = 0.01; Chi² = 35.17, df = 5 (P < 0.00001); I² = 86%								
Test for overall effect: Z = 5.62 (P < 0.00001)								
**Total (95% CI)**			**3122**			**3098**	**100.0%**	**0.27 [0.22, 0.31]**
Heterogeneity: Tau² = 0.02; Chi² = 669.54, df = 59 (P < 0.00001); I² = 91%								
Test for overall effect: Z = 11.87 (P < 0.00001)								
Test for subgroup differences: Chi² = 7.08, df = 7 (P = 0.42), I² = 1.1%								

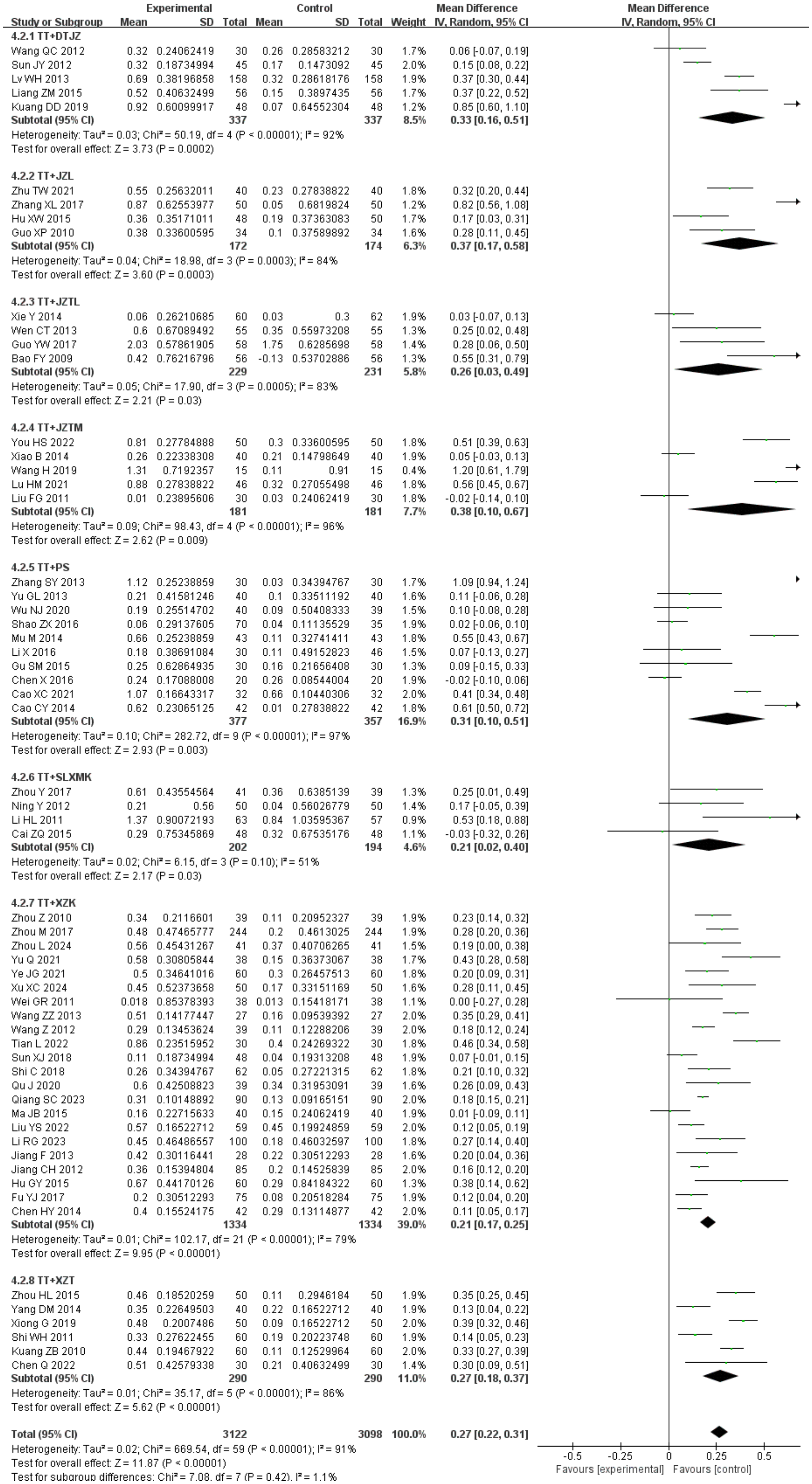

S7.5 LDL-C forest plot.

Study or Subgroup	Experimental Mean	Experimental SD	Experimental Total	Control Mean	Control SD	Control Total	Weight	Mean Difference IV, Random, 95% CI
**5.1.1 TT+DTJZ**								
Kuang DD 2019	-4.99	0.70632854	48	-4.02	0.63379808	48	1.9%	-0.97 [-1.24, -0.70]
Liang ZM 2015	-2.64	0.32908965	56	-1.77	0.49568135	56	2.1%	-0.87 [-1.03, -0.71]
Sun JY 2012	-0.61	0.42567593	45	-0.36	0.48445846	45	2.1%	-0.25 [-0.44, -0.06]
Wang QC 2012	-2.07	0.71630999	30	-1.76	0.66121101	30	1.7%	-0.31 [-0.66, 0.04]
**Subtotal (95% CI)**			**179**			**179**	**7.8%**	**-0.61 [-0.98, -0.23]**
Heterogeneity: Tau² = 0.13; Chi² = 34.06, df = 3 (P < 0.00001); I² = 91%								
Test for overall effect: Z = 3.15 (P = 0.002)								
**5.1.2 TT+JZL**								
Guo XP 2010	-1.78	0.73123184	34	-1.14	0.75901252	34	1.7%	-0.64 [-0.99, -0.29]
Hu XW 2015	-1.37	0.81461647	48	-0.79	0.815414	50	1.7%	-0.58 [-0.90, -0.26]
Zhang XL 2017	-2.06	0.78172885	50	-1.09	0.74545288	50	1.8%	-0.97 [-1.27, -0.67]
Zhu TW 2021	-2.13	0.88475985	40	-1.79	0.93578844	40	1.6%	-0.34 [-0.74, 0.06]
**Subtotal (95% CI)**			**172**			**174**	**6.8%**	**-0.65 [-0.91, -0.39]**
Heterogeneity: Tau² = 0.04; Chi² = 6.81, df = 3 (P = 0.08); I² = 56%								
Test for overall effect: Z = 4.97 (P < 0.00001)								
**5.1.3 TT+JZTL**								
Guo YW 2017	-2.01	0.98787651	58	-1.44	0.99859902	58	1.6%	-0.57 [-0.93, -0.21]
Wen CT 2013	-1.25	0.3176476	55	-0.57	0.32511536	55	2.2%	-0.68 [-0.80, -0.56]
Xie Y 2014	-1.39	0.81191132	60	-1.41	0.64093681	62	1.9%	0.02 [-0.24, 0.28]
**Subtotal (95% CI)**			**173**			**175**	**5.7%**	**-0.41 [-0.88, 0.05]**
Heterogeneity: Tau² = 0.15; Chi² = 22.94, df = 2 (P < 0.0001); I² = 91%								
Test for overall effect: Z = 1.75 (P = 0.08)								
**5.1.4 TT+JZTM**								
Liu FG 2011	-1.3	1.04589674	30	-1.21	0.95	30	1.3%	-0.09 [-0.60, 0.42]
Lu HM 2021	-0.58	0.90205321	46	-0.33	0.87469995	46	1.6%	-0.25 [-0.61, 0.11]
Wang H 2019	-1.21	1.59771712	15	-0.74	1.64328938	15	0.5%	-0.47 [-1.63, 0.69]
Xiao B 2014	-2.14	0.46872167	40	-1.74	0.53113087	40	2.0%	-0.40 [-0.62, -0.18]
You HS 2022	-0.56	1.3716049	50	-0.31	1.32094663	50	1.3%	-0.25 [-0.78, 0.28]
**Subtotal (95% CI)**			**181**			**181**	**6.7%**	**-0.32 [-0.49, -0.16]**
Heterogeneity: Tau² = 0.00; Chi² = 1.58, df = 4 (P = 0.81); I² = 0%								
Test for overall effect: Z = 3.82 (P = 0.0001)								
**5.1.5 TT+PS**								
Cao CY 2014	-1.82	0.82637764	42	-1.46	0.83216585	42	1.7%	-0.36 [-0.71, -0.01]
Cao XC 2021	-1.41	0.27622455	32	-1.34	0.26153394	32	2.2%	-0.07 [-0.20, 0.06]
Chen X 2016	-0.35	0.21794495	20	-0.25	0.10535654	20	2.2%	-0.10 [-0.21, 0.01]
Gu SM 2015	-0.9	0.79303216	30	-0.47	0.91536878	30	1.5%	-0.43 [-0.86, 0.00]
Li X 2016	-1.86	0.6819824	30	-1.16	1.30414723	46	1.4%	-0.70 [-1.15, -0.25]
Mu M 2014	-1.49	0.71267103	43	-1.62	0.40112342	43	1.9%	0.13 [-0.11, 0.37]
Shao ZX 2016	-0.45	0.53563047	70	-0.55	0.6408588	35	1.9%	0.10 [-0.15, 0.35]
Wu NJ 2020	-1.04	0.89286057	40	-0.87	0.55018179	39	1.7%	-0.17 [-0.50, 0.16]
Yu GL 2013	-1.02	0.97247108	40	-0.91	0.7922752	40	1.6%	-0.11 [-0.50, 0.28]
Zhang SY 2013	-1.23	0.81682311	30	-1.28	0.76726788	30	1.5%	0.05 [-0.35, 0.45]
**Subtotal (95% CI)**			**377**			**357**	**17.7%**	**-0.11 [-0.22, 0.00]**
Heterogeneity: Tau² = 0.01; Chi² = 17.91, df = 9 (P = 0.04); I² = 50%								
Test for overall effect: Z = 1.88 (P = 0.06)								
**5.1.6 TT+SLXMK**								
Cai ZQ 2015	-0.7	0.7014984	48	-0.68	0.47031904	48	2.0%	-0.02 [-0.26, 0.22]
Ning Y 2012	-0.9	0.71756533	50	-0.7	0.67638746	50	1.9%	-0.20 [-0.47, 0.07]
Zhou Y 2017	-2.35	0.66460515	41	-1.61	0.63976558	39	1.8%	-0.74 [-1.03, -0.45]
**Subtotal (95% CI)**			**139**			**137**	**5.7%**	**-0.31 [-0.73, 0.10]**
Heterogeneity: Tau² = 0.12; Chi² = 14.83, df = 2 (P = 0.0006); I² = 87%								
Test for overall effect: Z = 1.48 (P = 0.14)								
**5.1.7 TT+XZK**								
Chen HY 2014	-4.03	0.47947888	42	-3.59	0.4596738	42	2.0%	-0.44 [-0.64, -0.24]
Fu YJ 2017	-0.9	0.52915026	75	-0.5	0.55677644	75	2.1%	-0.40 [-0.57, -0.23]
Jiang CH 2012	-0.81	0.35383612	85	-0.57	0.35594944	85	2.2%	-0.24 [-0.35, -0.13]
Jiang F 2013	-1.05	0.6780118	28	-1.12	0.67357256	28	1.7%	0.07 [-0.28, 0.42]
Li RG 2023	-0.54	0.81184974	100	-0.27	0.78076885	100	2.0%	-0.27 [-0.49, -0.05]
Liu YS 2022	-1.31	0.11357817	59	-1.29	0.14933184	59	2.3%	-0.02 [-0.07, 0.03]
Ma JB 2015	-1.03	0.50764161	40	-1.08	0.4932545	40	2.0%	0.05 [-0.17, 0.27]
Qiang SC 2023	-1.67	0.44034078	90	-1.12	0.44530888	90	2.2%	-0.55 [-0.68, -0.42]
Qu J 2020	-1.34	0.59573484	39	-0.92	0.64505814	39	1.9%	-0.42 [-0.70, -0.14]
Shi C 2018	-1.56	0.71463277	62	-1.11	0.76216796	62	1.9%	-0.45 [-0.71, -0.19]
Sun XJ 2018	-2.03	0.29546573	48	-1.31	0.23259407	48	2.2%	-0.72 [-0.83, -0.61]
Tian L 2022	-1.34	0.67668309	30	-0.71	0.63316664	30	1.7%	-0.63 [-0.96, -0.30]
Wang Z 2012	-1.33	0.47507894	39	-0.84	0.3207803	39	2.1%	-0.49 [-0.67, -0.31]
Wang ZZ 2013	-1.54	0.42673177	27	-0.71	0.44305756	27	2.0%	-0.83 [-1.06, -0.60]
Wei GR 2011	-0.836	1.16808861	38	-0.827	1.18584822	38	1.2%	-0.01 [-0.54, 0.52]
Xu XC 2024	-3.28	0.87068938	50	-2.13	0.79642953	50	1.7%	-1.15 [-1.48, -0.82]
Ye JG 2021	-1.6	1.22882057	60	-1.1	1.30766968	60	1.4%	-0.50 [-0.95, -0.05]
Yu Q 2021	-0.57	0.5850641	38	-0.27	0.61991935	38	1.9%	-0.30 [-0.57, -0.03]
Zhou L 2024	-1.34	0.56320511	41	-0.92	0.59253692	41	1.9%	-0.42 [-0.67, -0.17]
Zhou M 2017	-0.55	0.79542442	244	-0.27	0.78172885	244	2.2%	-0.28 [-0.42, -0.14]
Zhou Z 2010	-1.66	0.52564246	39	-1.77	0.54249424	39	2.0%	0.11 [-0.13, 0.35]
**Subtotal (95% CI)**			**1274**			**1274**	**40.5%**	**-0.38 [-0.52, -0.24]**
Heterogeneity: Tau² = 0.09; Chi² = 282.44, df = 20 (P < 0.00001); I² = 93%								
Test for overall effect: Z = 5.31 (P < 0.00001)								
**5.1.8 TT+XZT**								
Chen Q 2022	-1.65	0.81504601	30	-1.22	0.71630999	30	1.6%	-0.43 [-0.82, -0.04]
Kuang ZB 2010	-1.78	1.1396052	60	-1.61	1.07670795	60	1.6%	-0.17 [-0.57, 0.23]
Shi WH 2011	-1.64	1.15658982	60	-1.35	1.75008571	60	1.2%	-0.29 [-0.82, 0.24]
Xiong G 2019	-2.26	0.37722672	50	-1.7	0.39281039	50	2.1%	-0.56 [-0.71, -0.41]
Yang DM 2014	-1.49	1.16464587	40	-0.44	1.07782188	40	1.3%	-1.05 [-1.54, -0.56]
Zhou HL 2015	-1.75	1.14450863	50	-1.23	1.14057003	50	1.4%	-0.52 [-0.97, -0.07]
**Subtotal (95% CI)**			**290**			**290**	**9.3%**	**-0.50 [-0.70, -0.31]**
Heterogeneity: Tau² = 0.02; Chi² = 8.64, df = 5 (P = 0.12); I² = 42%								
Test for overall effect: Z = 5.02 (P < 0.00001)								
**Total (95% CI)**			**2785**			**2767**	**100.0%**	**-0.38 [-0.46, -0.29]**
Heterogeneity: Tau² = 0.09; Chi² = 535.33, df = 55 (P < 0.00001); I² = 90%								
Test for overall effect: Z = 8.37 (P < 0.00001)								
Test for subgroup differences: Chi² = 25.66, df = 7 (P = 0.0006), I² = 72.7%								

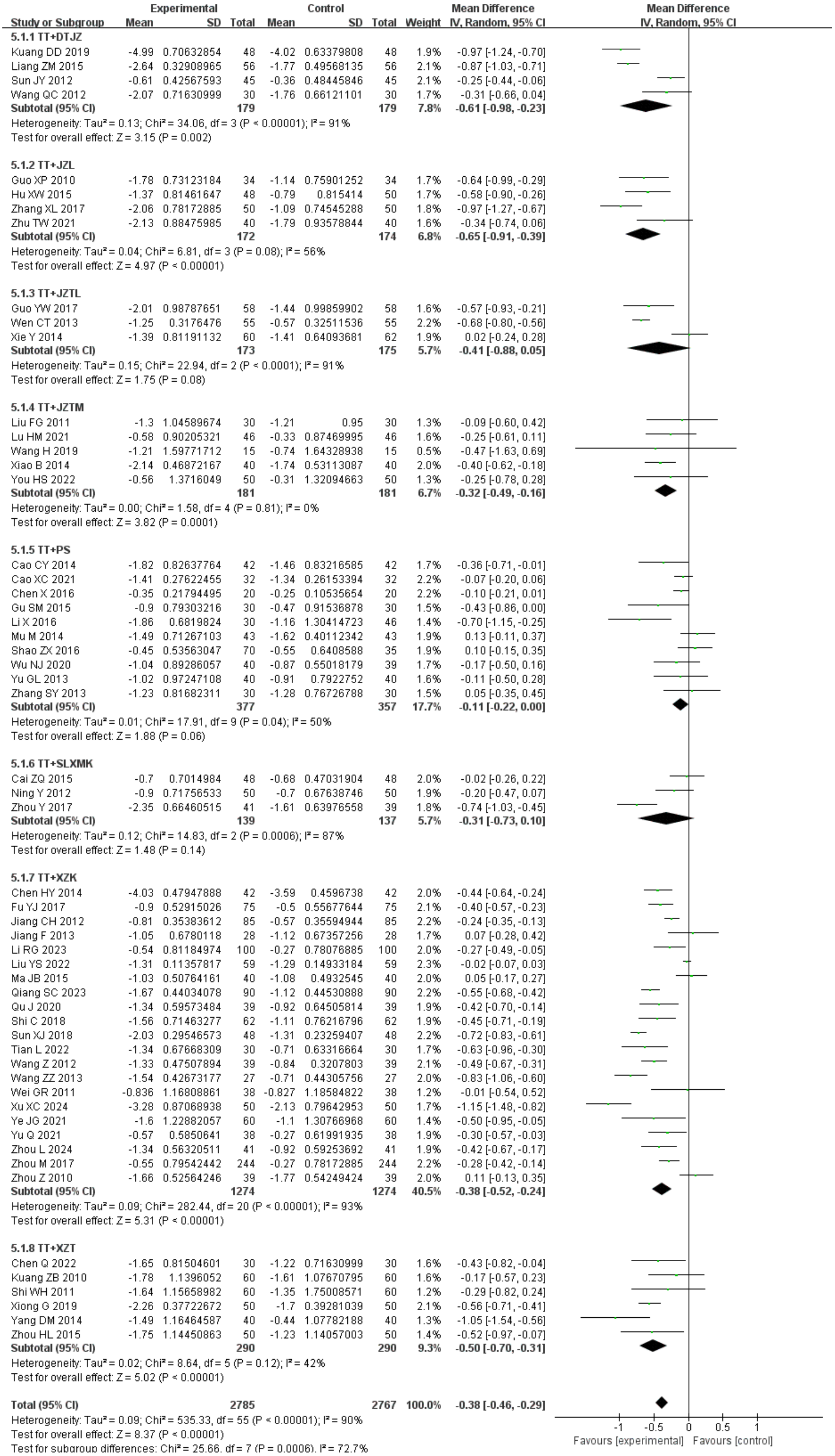

S8. Heterogeneity analysis and subgroup analysis of different interventions for different outcome indicators.

S8.1 Subgroup analysis of clinical total effective rate.

		TT+SLXMK	
**Subgroup**	**Criteria**	**OR[95% CI]**	**Subgroup Difference p-value**
Sample Size	≥120	2.51[1.22, 5.17]	0.4
	<120	6.28[0.84, 46.89]	
Duration	≥12	4.02[0.31, 52.20]	0.89
	<12	3.32[1.30, 8.48]	
Average Age	≥60	1.45[0.74, 2.82]	0.11
	<60	6.06[1.22, 30.13]	
Combined with other diseases	Yes	2.09[1.09, 4.03]	**0.004**
	No	16.77[4.79, 58.75]	

S8.1.1 TT+SLXMK subgroup analysis based on whether or not combined with other diseases (clinical total effective rate).

Study or Subgroup	Experimental Events	Experimental Total	Control Events	Control Total	Weight	Odds Ratio M-H, Random, 95% CI
**1.3.1 Combined with other diseases**						
Cai ZQ 2015	41	48	40	48	21.0%	1.17 [0.39, 3.53]
Feng ZP 2013	285	300	254	300	27.8%	3.44 [1.88, 6.31]
Li HL 2011	50	63	40	57	24.7%	1.63 [0.71, 3.76]
**Subtotal (95% CI)**		**411**		**405**	**73.4%**	**2.09 [1.09, 4.03]**
Total events	376		334			
Heterogeneity: Tau² = 0.16; Chi² = 3.78, df = 2 (P = 0.15); I² = 47%						
Test for overall effect: Z = 2.22 (P = 0.03)						
**1.3.2 Not combined with other diseases**						
Ning Y 2012	49	50	36	50	11.1%	19.06 [2.40, 151.60]
Zhou P 2012	33	35	18	35	15.4%	15.58 [3.23, 75.18]
**Subtotal (95% CI)**		**85**		**85**	**26.6%**	**16.77 [4.79, 58.75]**
Total events	82		54			
Heterogeneity: Tau² = 0.00; Chi² = 0.02, df = 1 (P = 0.88); I² = 0%						
Test for overall effect: Z = 4.41 (P < 0.0001)						
**Total (95% CI)**		**496**		**490**	**100.0%**	**3.49 [1.48, 8.21]**
Total events	458		388			
Heterogeneity: Tau² = 0.59; Chi² = 12.55, df = 4 (P = 0.01); I² = 68%						
Test for overall effect: Z = 2.86 (P = 0.004)						
Test for subgroup differences: Chi² = 8.32, df = 1 (P = 0.004), I² = 88.0%						

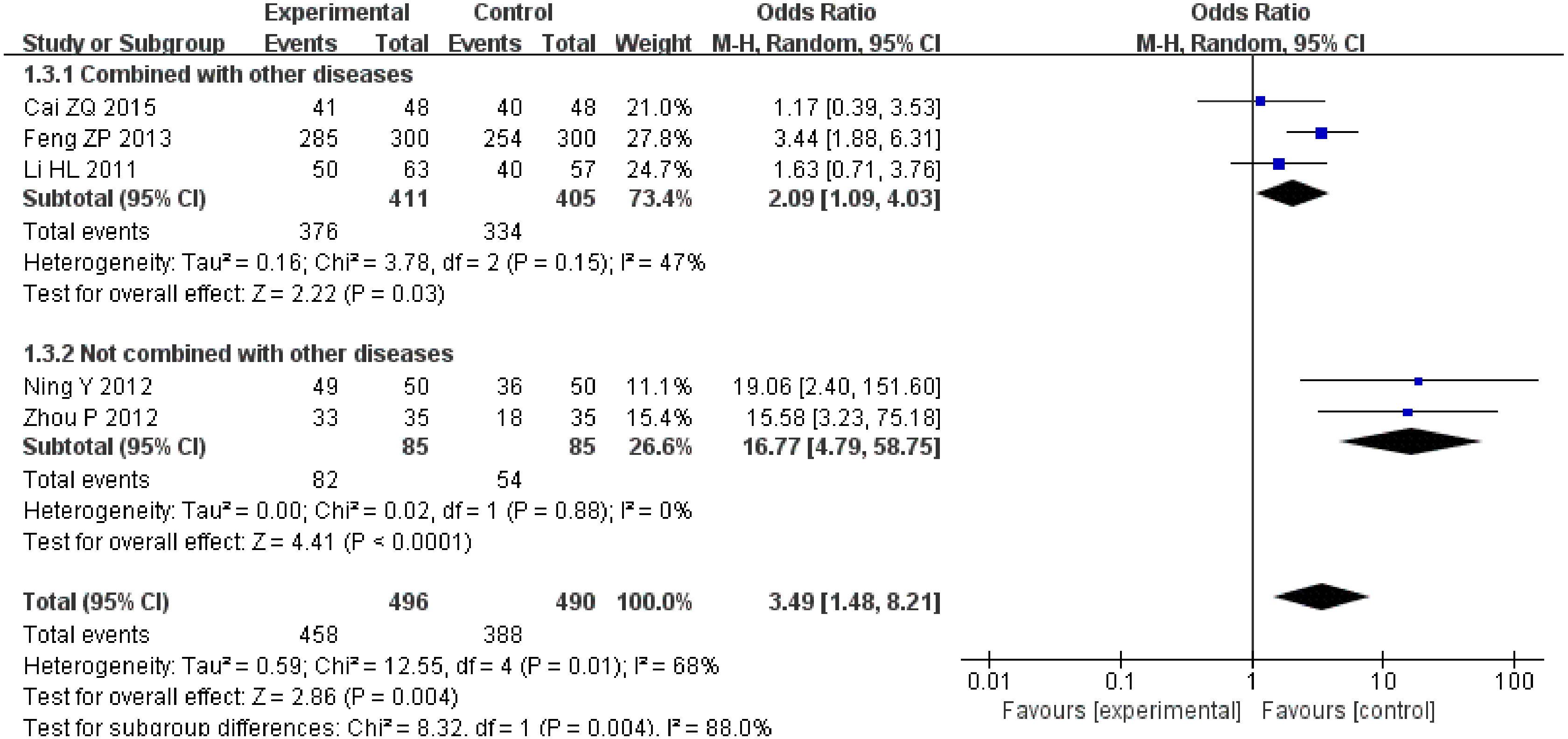

S8.2 Subgroup analysis of TC.

	TT+DTJZ			TT+JZTL			TT+JZTM		
Subgroup	Criteria	MD[95% CI]	Subgroup Difference p-value	Criteria	MD[95% CI]	Subgroup Difference p-value	Criteria	MD[95% CI]	Subgroup Difference p-value
Sample Size	≥100	-1.19 [-2.22, -0.16]	0.48	≥115	-0.39 [-0.96, 0.18]	0.47	≥80	-0.82 [-1.03, -0.62]	0.16
	<100	-0.79 [-1.20, -0.37]		<115	-0.65 [-1.07, -0.23]		<80	-0.32 [-1.00, 0.37]	
Duration	≥12	-0.89 [-1.48, -0.31]	0.84				≥8	-0.82 [-1.07, -0.57]	0.33
	<12	-0.98 [-1.67, -0.30]					<8	-0.42 [-1.19, 0.34]	
Average Age	≥60	-0.65 [-0.83, -0.47]	0.14	≥54	-0.39 [-0.96, 0.18]	0.47	≥60	-0.39 [-0.96, 0.18]	0.47
	<60	-1.16 [-1.81, -0.51]		<54	-0.65 [-1.07, -0.23]		<60	-0.65 [-1.07, -0.23]	
Combined with other diseases	Yes	-0.57 [-0.82, -0.32]	0.08				Yes	-0.91 [-1.11, -0.71]	0.07
	No	-1.18 [-1.81, -0.55]					No	-0.47 [-0.90, -0.04]	
Treatment with different TT	Atorvastatin	-0.89 [-1.48, -0.31]	0.84				Atorvastatin	-0.53 [-1.49, 0.44]	0.71
	Simvastatin	-0.98[-1.67, -0.30]					Simvastatin	-0.71 [-0.90, -0.52]	

	TT+SLXMK			TT+XZK			TT+XZT		
Subgroup	Criteria	MD[95% CI]	Subgroup Difference p-value	Criteria	MD[95% CI]	Subgroup Difference p-value	Criteria	MD[95% CI]	Subgroup Difference p-value
Sample Size	≥100	-0.62 [-1.87, 0.64]	0.75	≥95	-0.75 [-0.96, -0.54]	**0.003**	≥100	-0.12 [-0.53, 0.30]	**0.002**
	<100	-0.83 [-1.16, -0.50]		<95&>75	-0.26 [-0.46, -0.06]		<100	-0.81 [-0.97, -0.65]	
				≤75	-0.80 [-1.61, 0.01]				
Duration	≥12	-0.83 [-1.16, -0.50]	0.75	≥12	-0.66 [-0.94, -0.39]	0.33	≥12	-0.72 [-1.26, -0.17]	0.9
	<12	-0.62 [-1.87, 0.64]		<12	-0.51 [-0.68, -0.34]		<12	-0.75 [-0.94, -0.57]	
Average Age	≥60	-0.47 [-1.39, 0.45]	0.31	≥60	-0.57 [-0.80, -0.34]	0.74	≥50	-0.63 [-1.05, -0.22]	0.27
	<60	-1.00 [-1.44, -0.56]		<60	-0.64 [-0.95, -0.32]		<50	-0.96 [-1.40, -0.53]	
Combined with other diseases				Yes	-0.57 [-0.91, -0.23]	0.83			
				No	-0.61 [-0.83, -0.39]				
Treatment with different TT				Atorvastatin	-0.74 [-1.02, -0.46]	**0.002**			
				Simvastatin	-0.17 [-0.38, 0.04]				
				Pitavastatin	-0.83 [-1.42, -0.23]				

S8.2.1 TT+XZK subgroup analysis based on sample size (TC).

Study or Subgroup	Experimental Mean	Experimental SD	Experimental Total	Control Mean	Control SD	Control Total	Weight	Mean Difference IV, Random, 95% CI
**2.17.1 SampleSize ≥95**								
Fu YJ 2017	-2	0.87177979	75	-1.2	0.9539392	75	4.4%	-0.80 [-1.09, -0.51]
Hu GY 2015	-2	1.12343224	60	-1.43	1.06719258	60	4.0%	-0.57 [-0.96, -0.18]
Jiang CH 2012	-0.83	0.55380502	85	-0.47	0.54990908	85	4.8%	-0.36 [-0.53, -0.19]
Li RG 2023	-1.52	1.5400974	100	-0.66	1.44564864	100	4.0%	-0.86 [-1.27, -0.45]
Liu YS 2022	-2.25	1.06296754	59	-1.93	1.04919016	59	4.1%	-0.32 [-0.70, 0.06]
Qiang SC 2023	-1.78	0.55434646	90	-1.08	0.59	90	4.8%	-0.70 [-0.87, -0.53]
Shi C 2018	-1.96	0.80074965	62	-1.33	0.79699435	62	4.5%	-0.63 [-0.91, -0.35]
Sun XJ 2018	-1.46	0.55569776	48	-0.59	0.55973208	48	4.6%	-0.87 [-1.09, -0.65]
Xu XC 2024	-3.06	0.67089492	50	-1.53	0.62385896	50	4.5%	-1.53 [-1.78, -1.28]
Ye JG 2021	-3	1.73493516	60	-2.3	1.85202592	60	3.1%	-0.70 [-1.34, -0.06]
Zhou M 2017	-1.51	1.52502459	244	-0.67	1.46563979	244	4.5%	-0.84 [-1.11, -0.57]
**Subtotal (95% CI)**			**933**			**933**	**47.3%**	**-0.75 [-0.96, -0.54]**
Heterogeneity: Tau² = 0.10; Chi² = 65.66, df = 10 (P < 0.00001); I² = 85%								
Test for overall effect: Z = 6.99 (P < 0.00001)								
**2.17.2 75 < SampleSize<95**								
Chen HY 2014	-3.91	0.61506097	42	-3.5	0.55018179	42	4.6%	-0.41 [-0.66, -0.16]
Ma JB 2015	-1.06	0.68439754	40	-1.16	0.57887823	40	4.5%	0.10 [-0.18, 0.38]
Qu J 2020	-1.47	1.21024791	39	-0.98	1.30219046	39	3.4%	-0.49 [-1.05, 0.07]
Wang Z 2012	-2.02	0.94556861	39	-1.91	0.7014984	39	4.1%	-0.11 [-0.48, 0.26]
Wei GR 2011	-1.691	0.69733995	38	-1.514	0.43675622	38	4.5%	-0.18 [-0.44, 0.08]
Yu Q 2021	-1.55	1.15060854	38	-0.68	1.21786699	38	3.5%	-0.87 [-1.40, -0.34]
Zhou L 2024	-1.54	0.9477869	41	-1.03	0.94694245	41	4.0%	-0.51 [-0.92, -0.10]
Zhou Z 2010	-2.96	0.79542442	39	-2.99	0.59354865	39	4.3%	0.03 [-0.28, 0.34]
**Subtotal (95% CI)**			**316**			**316**	**32.9%**	**-0.26 [-0.46, -0.06]**
Heterogeneity: Tau² = 0.05; Chi² = 18.78, df = 7 (P = 0.009); I² = 63%								
Test for overall effect: Z = 2.51 (P = 0.01)								
**2.17.3 SampleSize ≤75**								
Gui YF 2019	-2.21	1.00881118	36	-1.55	1.02586549	36	3.7%	-0.66 [-1.13, -0.19]
Huang CC 2014	-2.37	0.99050492	30	-2.4	0.98305646	25	3.5%	0.03 [-0.49, 0.55]
Jiang F 2013	-1.72	0.49122296	28	-1.44	0.57662813	28	4.5%	-0.28 [-0.56, 0.00]
Tian L 2022	-1.94	0.81504601	30	-0.84	0.77948701	30	4.0%	-1.10 [-1.50, -0.70]
Wang ZZ 2013	-2.87	0.68789534	27	-1.05	0.71042241	27	4.1%	-1.82 [-2.19, -1.45]
**Subtotal (95% CI)**			**151**			**146**	**19.8%**	**-0.77 [-1.42, -0.12]**
Heterogeneity: Tau² = 0.50; Chi² = 53.90, df = 4 (P < 0.00001); I² = 93%								
Test for overall effect: Z = 2.34 (P = 0.02)								
**Total (95% CI)**			**1400**			**1395**	**100.0%**	**-0.60 [-0.78, -0.42]**
Heterogeneity: Tau² = 0.17; Chi² = 193.25, df = 23 (P < 0.00001); I² = 88%								
Test for overall effect: Z = 6.49 (P < 0.00001)								
Test for subgroup differences: Chi² = 11.83, df = 2 (P = 0.003), I² = 83.1%								

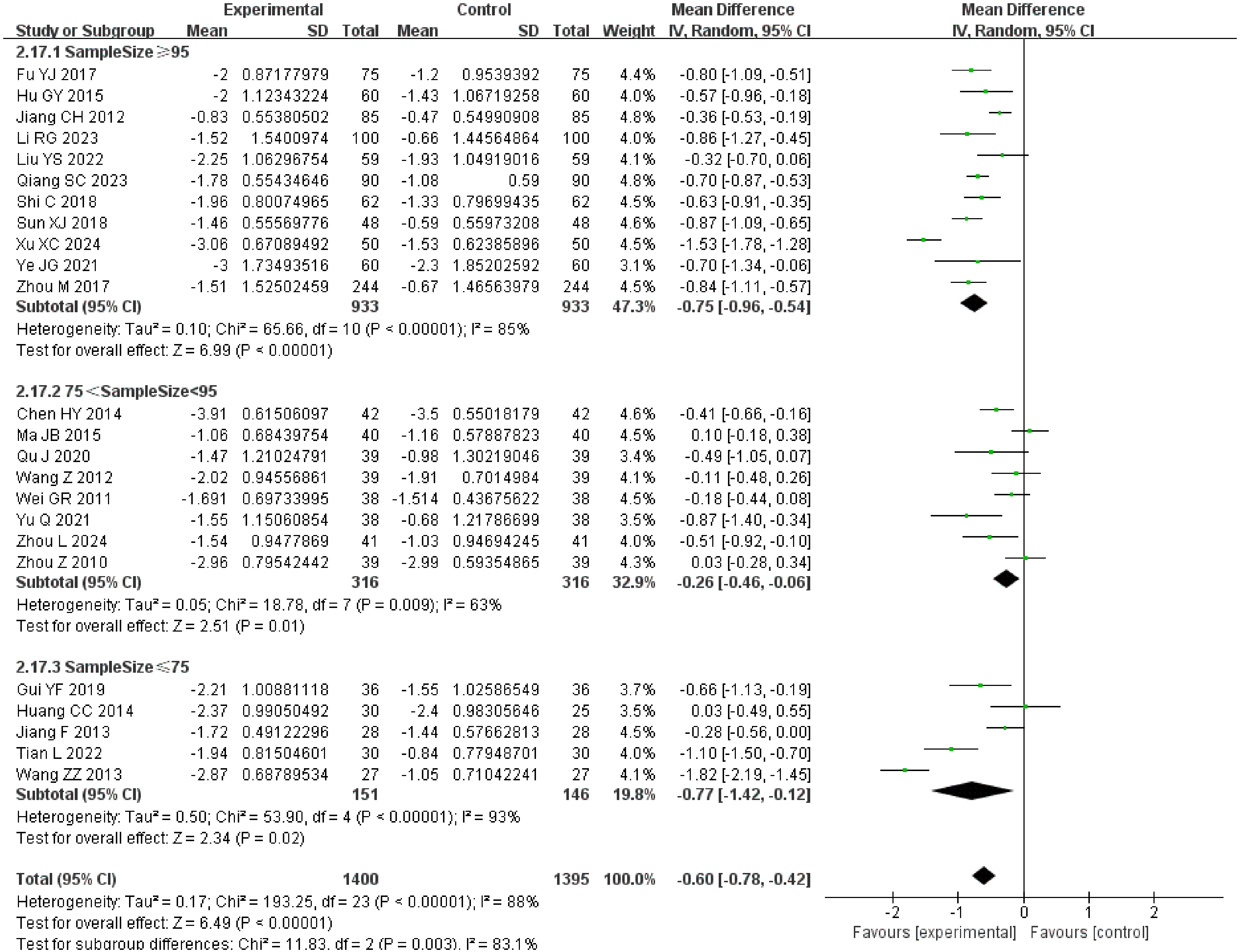

S8.2.2 TT+XZK subgroup analysis based on different TT (TC).

	Experimental			Control				Mean Difference
**Study or Subgroup**	**Mean**	**SD**	**Total**	**Mean**	**SD**	**Total**	**Weight**	**IV, Random, 95% CI**
**2.25.1 Atorvastatin**								
Chen HY 2014	-3.91	0.61506097	42	-3.5	0.55018179	42	5.2%	-0.41 [-0.66, -0.16]
Fu YJ 2017	-2	0.87177979	75	-1.2	0.9539392	75	5.1%	-0.80 [-1.09, -0.51]
Hu GY 2015	-2	1.12343224	60	-1.43	1.06719258	60	4.7%	-0.57 [-0.96, -0.18]
Jiang F 2013	-1.72	0.49122296	28	-1.44	0.57662813	28	5.1%	-0.28 [-0.56, 0.00]
Li RG 2023	-1.52	1.5400974	100	-0.66	1.44564864	100	4.6%	-0.86 [-1.27, -0.45]
Liu YS 2022	-2.25	1.06296754	59	-1.93	1.04919016	59	4.8%	-0.32 [-0.70, 0.06]
Wang Z 2012	-2.02	0.94556861	39	-1.91	0.7014984	39	4.8%	-0.11 [-0.48, 0.26]
Wang ZZ 2013	-2.87	0.68789534	27	-1.05	0.71042241	27	4.8%	-1.82 [-2.19, -1.45]
Xu XC 2024	-3.06	0.67089492	50	-1.53	0.62385896	50	5.2%	-1.53 [-1.78, -1.28]
Ye JG 2021	-3	1.73493516	60	-2.3	1.85202592	60	3.8%	-0.70 [-1.34, -0.06]
Yu Q 2021	-1.55	1.15060854	38	-0.68	1.21786699	38	4.2%	-0.87 [-1.40, -0.34]
Zhou L 2024	-1.54	0.9477869	41	-1.03	0.94694245	41	4.7%	-0.51 [-0.92, -0.10]
Zhou M 2017	-1.51	1.52502459	244	-0.67	1.46563979	244	5.1%	-0.84 [-1.11, -0.57]
**Subtotal (95% CI)**			**863**			**863**	**62.0%**	**-0.74 [-1.02, -0.46]**
Heterogeneity: Tau² = 0.23; Chi² = 105.32, df = 12 (P < 0.00001); I² = 89%								
Test for overall effect: Z = 5.15 (P < 0.00001)								
**2.25.2 Simvastatin**								
Gui YF 2019	-2.21	1.00881118	36	-1.55	1.02586549	36	4.4%	-0.66 [-1.13, -0.19]
Huang CC 2014	-2.37	0.99050492	30	-2.4	0.98305646	25	4.2%	0.03 [-0.49, 0.55]
Jiang CH 2012	-0.83	0.55380502	85	-0.47	0.54990908	85	5.4%	-0.36 [-0.53, -0.19]
Ma JB 2015	-1.06	0.68439754	40	-1.16	0.57887823	40	5.1%	0.10 [-0.18, 0.38]
Wei GR 2011	-1.691	0.69733995	38	-1.514	0.43675622	38	5.2%	-0.18 [-0.44, 0.08]
Zhou Z 2010	-2.96	0.79542442	39	-2.99	0.59354865	39	5.0%	0.03 [-0.28, 0.34]
**Subtotal (95% CI)**			**268**			**263**	**29.3%**	**-0.17 [-0.38, 0.04]**
Heterogeneity: Tau² = 0.04; Chi² = 14.59, df = 5 (P = 0.01); I² = 66%								
Test for overall effect: Z = 1.57 (P = 0.12)								
**2.25.3 Pitavastatin**								
Qu J 2020	-1.47	1.21024791	39	-0.98	1.30219046	39	4.1%	-0.49 [-1.05, 0.07]
Tian L 2022	-1.94	0.81504601	30	-0.84	0.77948701	30	4.7%	-1.10 [-1.50, -0.70]
**Subtotal (95% CI)**			**69**			**69**	**8.8%**	**-0.83 [-1.42, -0.23]**
Heterogeneity: Tau² = 0.12; Chi² = 3.01, df = 1 (P = 0.08); I² = 67%								
Test for overall effect: Z = 2.73 (P = 0.006)								
**Total (95% CI)**			**1200**			**1195**	**100.0%**	**-0.58 [-0.80, -0.36]**
Heterogeneity: Tau² = 0.22; Chi² = 183.83, df = 20 (P < 0.00001); I² = 89%								
Test for overall effect: Z = 5.22 (P < 0.00001)								
Test for subgroup differences: Chi² = 12.30, df = 2 (P = 0.002), I² = 83.7%								

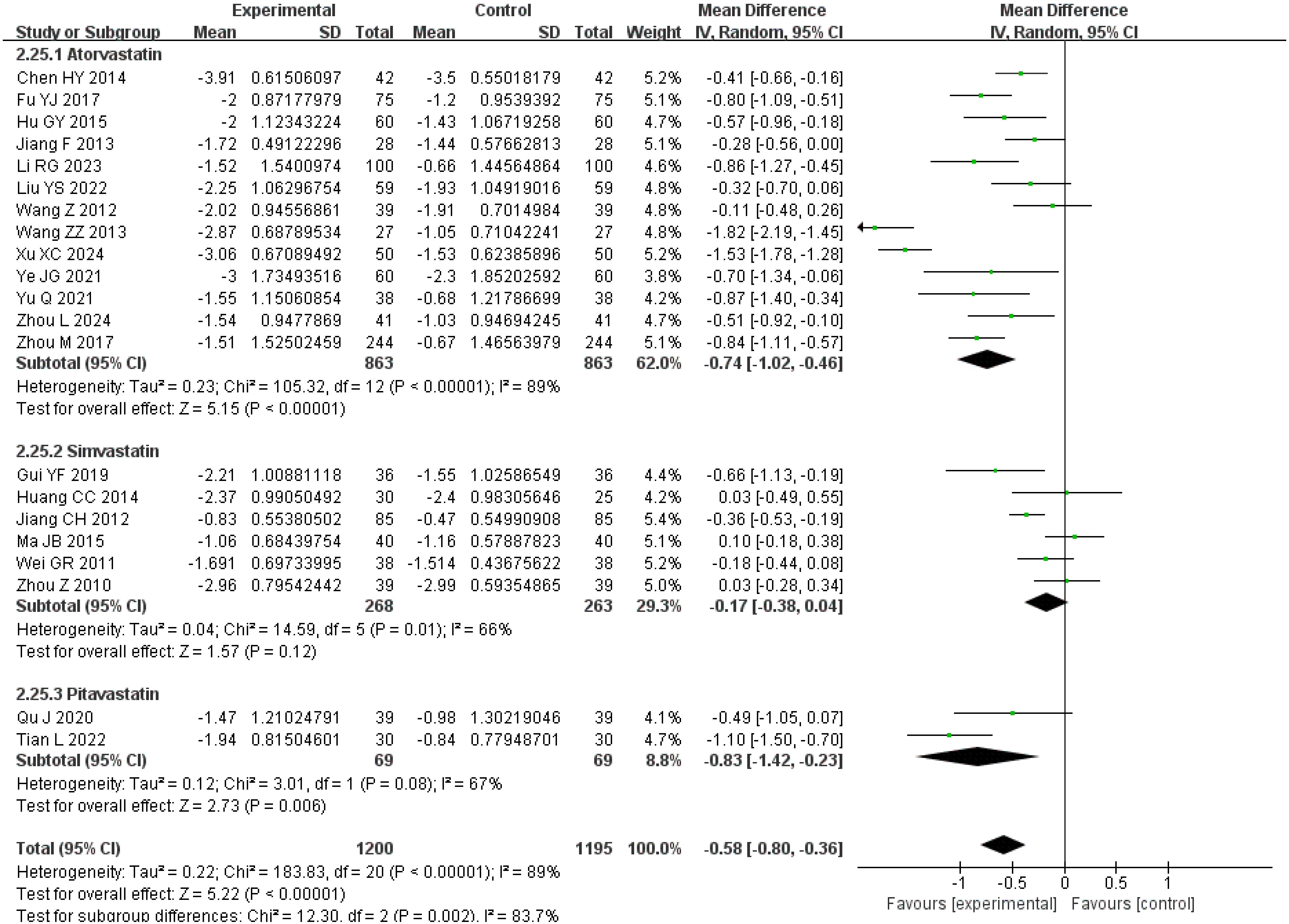

S8.2.3 TT+XZT subgroup analysis based on sample size (TC).

Study or Subgroup	Experimental Mean	Experimental SD	Experimental Total	Control Mean	Control SD	Control Total	Weight	Mean Difference IV, Random, 95% CI
**2.21.1 SampleSize >100**								
Kuang ZB 2010	-2.14	1.27	60	-2.12	0.88898819	60	19.8%	-0.02 [-0.41, 0.37]
Shi WH 2011	-1.73	3.12635571	60	-1.15	2.20469953	60	8.0%	-0.58 [-1.55, 0.39]
**Subtotal (95% CI)**			**120**			**120**	**27.8%**	**-0.12 [-0.53, 0.30]**
Heterogeneity: Tau² = 0.01; Chi² = 1.10, df = 1 (P = 0.29); I² = 9%								
Test for overall effect: Z = 0.55 (P = 0.58)								
**2.21.2 SampleSize ≤100**								
Chen Q 2022	-1.76	0.95566731	30	-1	0.80721744	30	18.2%	-0.76 [-1.21, -0.31]
Xiong G 2019	-3.72	0.46518813	50	-2.96	0.4927474	50	25.4%	-0.76 [-0.95, -0.57]
Yang DM 2014	-2.33	1.95138412	40	-1.16	1.40758659	40	11.3%	-1.17 [-1.92, -0.42]
Zhou HL 2015	-2.16	1.26003968	50	-1.1	1.1931471	50	17.3%	-1.06 [-1.54, -0.58]
**Subtotal (95% CI)**			**170**			**170**	**72.2%**	**-0.81 [-0.97, -0.65]**
Heterogeneity: Tau² = 0.00; Chi² = 2.25, df = 3 (P = 0.52); I² = 0%								
Test for overall effect: Z = 9.99 (P < 0.00001)								
**Total (95% CI)**			**290**			**290**	**100.0%**	**-0.70 [-1.02, -0.37]**
Heterogeneity: Tau² = 0.10; Chi² = 15.74, df = 5 (P = 0.008); I² = 68%								
Test for overall effect: Z = 4.19 (P < 0.0001)								
Test for subgroup differences: Chi² = 9.27, df = 1 (P = 0.002), I² = 89.2%								

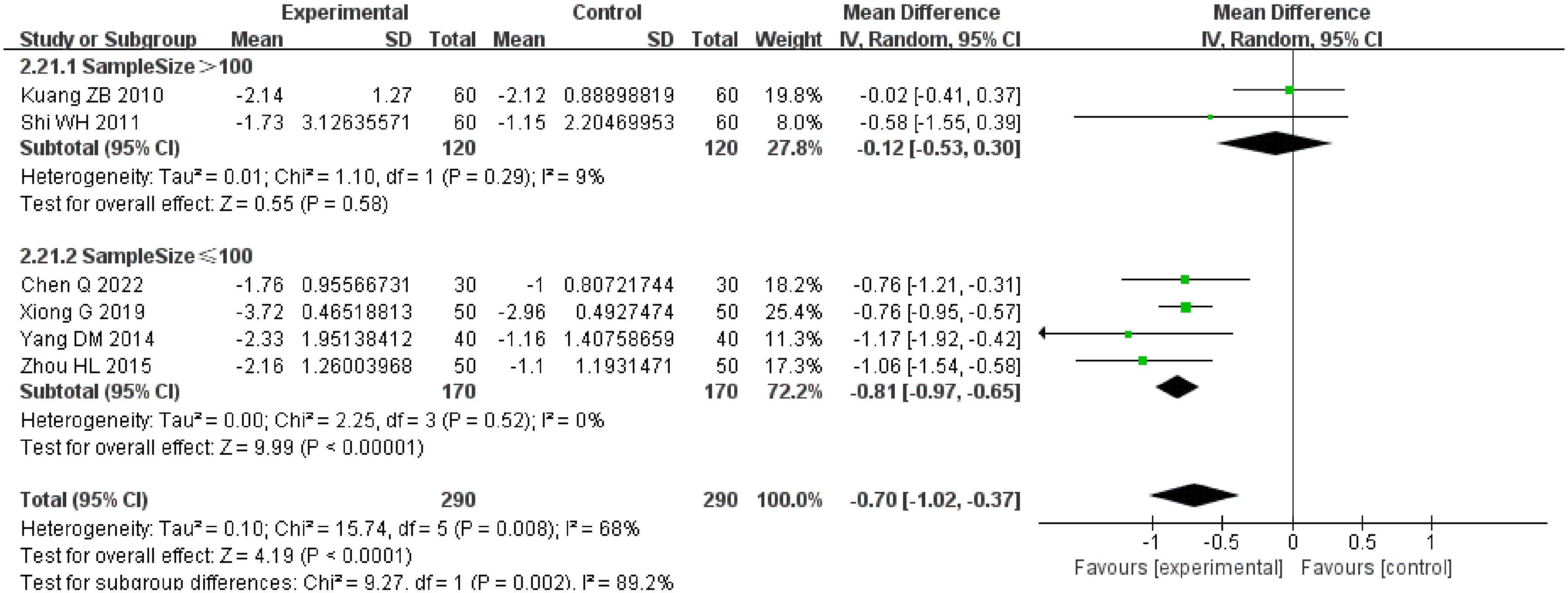

S8.3 TG

	TT+DTJZ			TT+JZL			TT+JZTM		
Subgroup	Criteria	MD[95% CI]	Subgroup Difference p-value	Criteria	MD[95% CI]	Subgroup Difference p-value	Criteria	MD[95% CI]	Subgroup Difference p-value
Sample Size	≥95	-0.61 [-0.73, -0.49]	**0.0001**	≥95	-0.68 [-0.91, -0.44]	0.2	≥90	-0.88 [-1.27, -0.49]	**0.001**
	<95	-0.27 [-0.37, -0.16]		<95	-0.34 [-0.79, 0.11]		<90	-0.16 [-0.35, 0.04]	
Duration	≥12	-0.51 [-0.98, -0.04]	0.88	≥12	-0.64 [-0.83, -0.45]	**0.04**	≥8	-0.48 [-1.03, 0.06]	0.96
	<12	-0.47 [-0.71, -0.23]		<12	-0.21 [-0.56, 0.15]		<8	-0.51 [-1.17, 0.16]	
Average Age	≥60	-0.46 [-0.66, -0.26]	0.68	≥50	-0.64 [-0.83, -0.45]	**0.04**	≥60	-0.48 [-1.03, 0.06]	0.96
	<60	-0.53 [-0.84, -0.22]		<50	-0.21 [-0.56, 0.15]		<60	-0.51 [-1.17, 0.16]	
Combined with other diseases	Yes	-0.27 [-0.37, -0.16]	**0.0001**	Yes	-0.55 [-0.82, -0.29]	0.67	Yes	-0.42 [-1.15, 0.30]	0.74
	No	-0.61 [-0.73, -0.49]		No	-0.42 [-1.00, 0.17]		No	-0.57 [-1.00, -0.13]	
Treatment with different TT	Atorvastatin	-0.51 [-0.98, -0.04]	0.88				Atorvastatin	-0.12 [-0.33, 0.09]	**0.0007**
	Simvastatin	-0.47 [-0.71, -0.23]					Simvastatin	-0.80 [-1.14, -0.46]	

	TT+PS			TT+SLXMK			TT+XZK		
Subgroup	Criteria	MD[95% CI]	Subgroup Difference p-value	Criteria	MD[95% CI]	Subgroup Difference p-value	Criteria	MD[95% CI]	Subgroup Difference p-value
Sample Size	≥80	-0.78 [-1.12, -0.44]	0.13	≥100	-0.54 [-1.02, -0.06]	0.19	≥100	-0.38 [-0.56, -0.20]	0.59
	<80	-0.45 [-0.70, -0.19]		<100	-0.19 [-0.40, 0.03]		<100	-0.32 [-0.46, -0.18]	
Duration	≥10	-0.83 [-1.20, -0.46]	0.13				≥12	-0.37 [-0.48, -0.26]	0.61
	<10	-0.49 [-0.74, -0.23]					<12	-0.31 [-0.52, -0.09]	
Average Age	≥58	-0.66 [-0.91, -0.41]	0.3	≥60	-0.26 [-0.49, -0.04]	0.57	≥60	-0.32 [-0.48, -0.15]	0.6
	<58	-0.40 [-0.82, 0.03]		<60	-0.46 [-1.09, 0.18]		<60	-0.38 [-0.53, -0.22]	
Combined with other diseases	Yes	-0.87 [-1.18, -0.57]	**0.04**				Yes	-0.35 [-0.50, -0.19]	0.92
	No	-0.47 [-0.70, -0.23]					No	-0.34 [-0.49, -0.19]	
Treatment with different TT	Atorvastatin	-0.55 [-0.95, -0.15]	0.4				Atorvastatin	-0.37 [-0.54, -0.20]	0.1
	Rosuvastatin	-0.76 [-1.03, -0.49]					Simvastatin	-0.18 [-0.34, -0.01]	

S8.3.1 TT+PS subgroup analysis based on whether or not combined with other diseases (TG).

Study or Subgroup	Experimental Mean	Experimental SD	Experimental Total	Control Mean	Control SD	Control Total	Weight	Mean Difference IV, Random, 95% CI
**3.17.1 Combined with other diseases**								
Cao XC 2021	-2.29	1.3716049	32	-1.83	1.31053424	32	7.0%	-0.46 [-1.12, 0.20]
Shao ZX 2016	-2.38	0.67357256	70	-1.29	0.74054034	35	13.4%	-1.09 [-1.38, -0.80]
Wu NJ 2020	-1.63	1.6021548	40	-1.12	2.10256986	39	5.2%	-0.51 [-1.34, 0.32]
Zhang SY 2013	-2.33	1.11498879	30	-1.41	1.12769677	30	8.2%	-0.92 [-1.49, -0.35]
**Subtotal (95% CI)**			**172**			**136**	**33.8%**	**-0.87 [-1.18, -0.57]**
Heterogeneity: Tau² = 0.03; Chi² = 4.11, df = 3 (P = 0.25); I² = 27%								
Test for overall effect: Z = 5.59 (P < 0.00001)								
**3.17.2 Not combined with other diseases**								
Cao CY 2014	-2.31	1.10891839	42	-1.97	1.00642933	42	10.2%	-0.34 [-0.79, 0.11]
Chen X 2016	-1.4	0.3508561	20	-0.79	0.34394767	20	14.9%	-0.61 [-0.83, -0.39]
Gu SM 2015	-1.11	0.8908423	30	-0.92	0.9367497	30	10.0%	-0.19 [-0.65, 0.27]
Li X 2016	-1.43	0.77543536	30	-1.33	0.8107404	46	11.9%	-0.10 [-0.46, 0.26]
Mu M 2014	-2.19	0.78619336	43	-1.31	1.4650256	43	9.4%	-0.88 [-1.38, -0.38]
Yu GL 2013	-1.07	1.26099167	40	-0.37	0.83719771	40	9.9%	-0.70 [-1.17, -0.23]
**Subtotal (95% CI)**			**205**			**221**	**66.2%**	**-0.47 [-0.70, -0.23]**
Heterogeneity: Tau² = 0.04; Chi² = 10.80, df = 5 (P = 0.06); I² = 54%								
Test for overall effect: Z = 3.90 (P < 0.0001)								
**Total (95% CI)**			**377**			**357**	**100.0%**	**-0.59 [-0.81, -0.36]**
Heterogeneity: Tau² = 0.08; Chi² = 25.18, df = 9 (P = 0.003); I² = 64%								
Test for overall effect: Z = 5.10 (P < 0.00001)								
Test for subgroup differences: Chi² = 4.31, df = 1 (P = 0.04), I² = 76.8%								

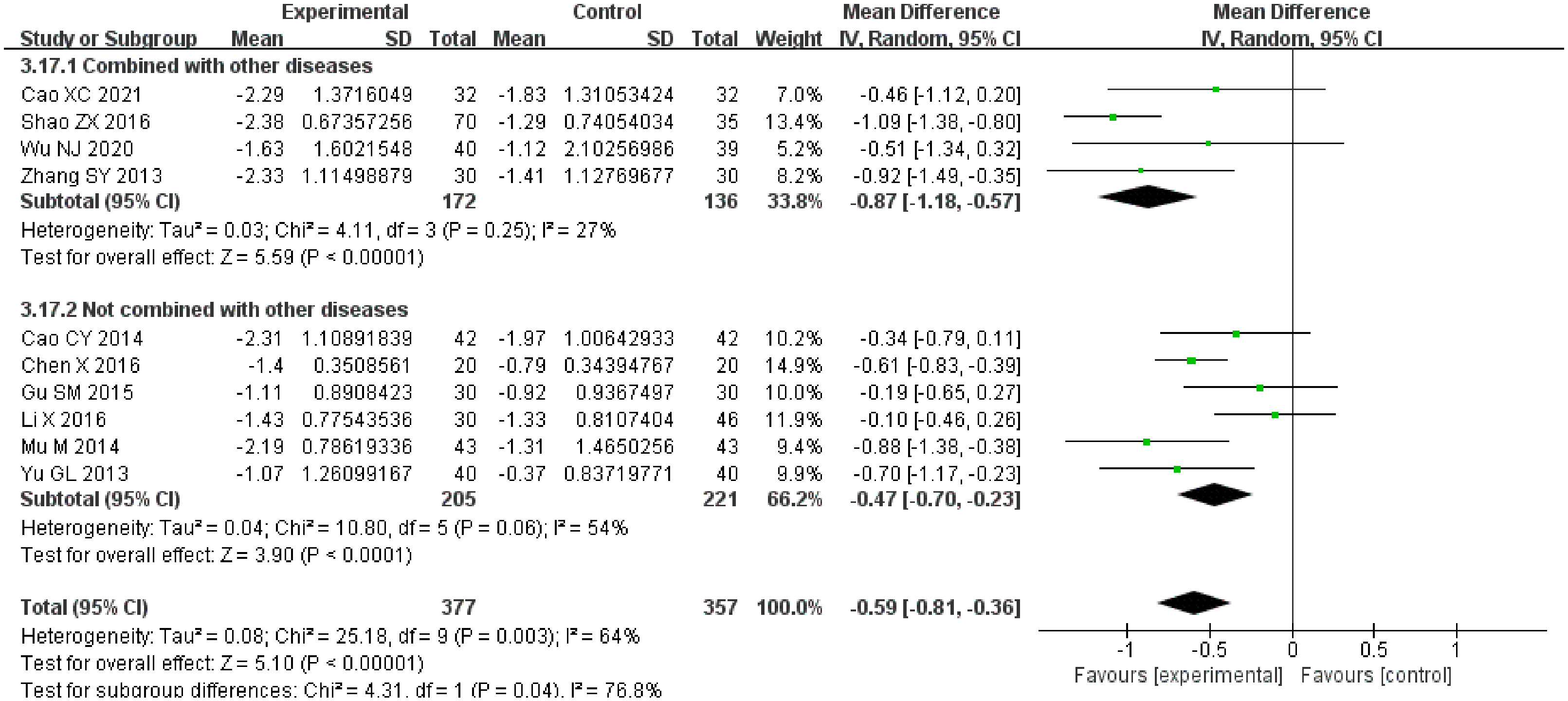

S8.3.2 TT+DTJZ subgroup analysis based on sample size (TG).

Study or Subgroup	Experimental Mean	Experimental SD	Experimental Total	Control Mean	Control SD	Control Total	Weight	Mean Difference IV, Random, 95% CI
**3.4.1 SampleSize ≥95**								
Kuang DD 2019	-1.23	0.63788714	48	-0.52	0.67734777	48	18.8%	-0.71 [-0.97, -0.45]
Liang ZM 2015	-1.34	0.48877398	56	-0.84	0.49426713	56	22.4%	-0.50 [-0.68, -0.32]
Lv WH 2013	-1.03	0.77826731	158	-0.37	0.77672389	158	22.9%	-0.66 [-0.83, -0.49]
**Subtotal (95% CI)**			**262**			**262**	**64.1%**	**-0.61 [-0.73, -0.49]**
Heterogeneity: Tau² = 0.00; Chi² = 2.28, df = 2 (P = 0.32); I² = 12%								
Test for overall effect: Z = 9.81 (P < 0.00001)								
**3.4.2 SampleSize <95**								
Sun JY 2012	-1	0.23065125	45	-0.73	0.29614186	45	25.3%	-0.27 [-0.38, -0.16]
Wang QC 2012	-1.46	1.04541858	30	-1.24	0.87846457	30	10.6%	-0.22 [-0.71, 0.27]
**Subtotal (95% CI)**			**75**			**75**	**35.9%**	**-0.27 [-0.37, -0.16]**
Heterogeneity: Tau² = 0.00; Chi² = 0.04, df = 1 (P = 0.84); I² = 0%								
Test for overall effect: Z = 4.90 (P < 0.00001)								
**Total (95% CI)**			**337**			**337**	**100.0%**	**-0.49 [-0.69, -0.28]**
Heterogeneity: Tau² = 0.04; Chi² = 20.71, df = 4 (P = 0.0004); I² = 81%								
Test for overall effect: Z = 4.70 (P < 0.00001)								
Test for subgroup differences: Chi² = 17.05, df = 1 (P < 0.0001), I² = 94.1%								

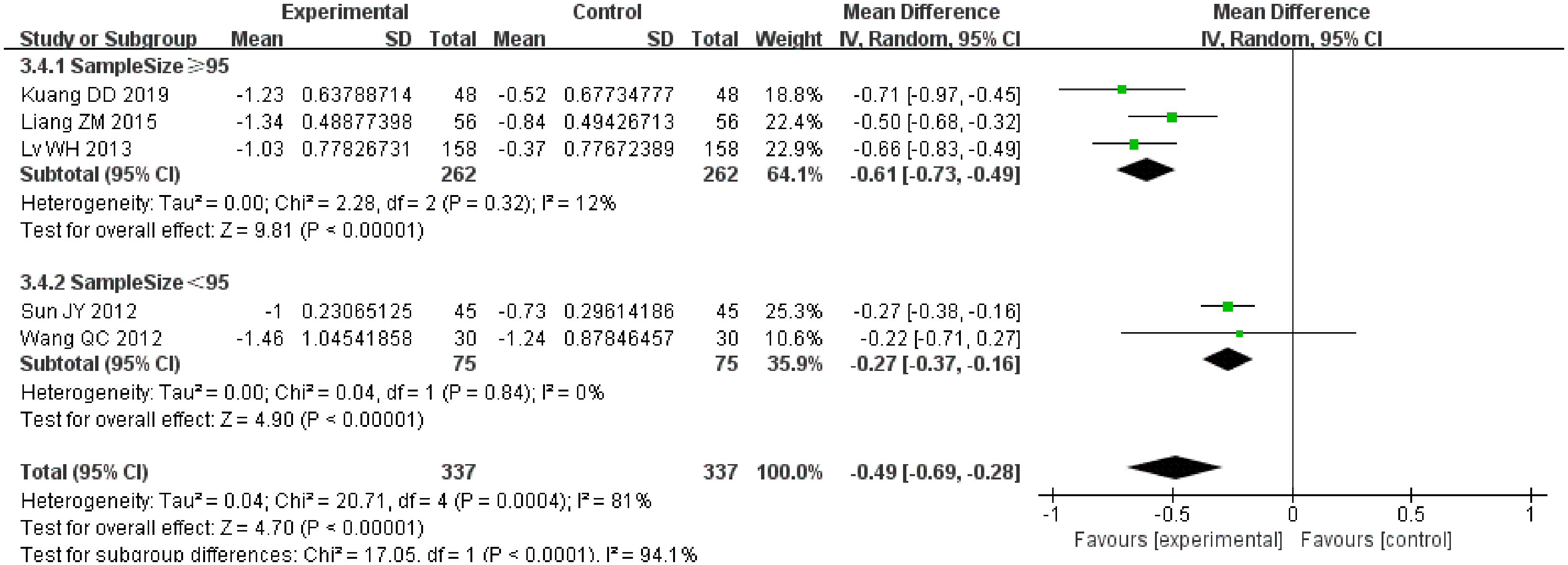

S8.3.3 TT+DTJZ subgroup analysis based on whether or not combined with other diseases (TG).

Study or Subgroup	Experimental Mean	Experimental SD	Experimental Total	Control Mean	Control SD	Control Total	Weight	Mean Difference IV, Random, 95% CI
**3.2.1 Combined with other diseases**								
Sun JY 2012	-1	0.23065125	45	-0.73	0.29614186	45	25.3%	-0.27 [-0.38, -0.16]
Wang QC 2012	-1.46	1.04541858	30	-1.24	0.87846457	30	10.6%	-0.22 [-0.71, 0.27]
**Subtotal (95% CI)**			**75**			**75**	**35.9%**	**-0.27 [-0.37, -0.16]**
Heterogeneity: Tau² = 0.00; Chi² = 0.04, df = 1 (P = 0.84); I² = 0%								
Test for overall effect: Z = 4.90 (P < 0.00001)								
**3.2.2 Not combined with other diseases**								
Kuang DD 2019	-1.23	0.63788714	48	-0.52	0.67734777	48	18.8%	-0.71 [-0.97, -0.45]
Liang ZM 2015	-1.34	0.48877398	56	-0.84	0.49426713	56	22.4%	-0.50 [-0.68, -0.32]
Lv WH 2013	-1.03	0.77826731	158	-0.37	0.77672389	158	22.9%	-0.66 [-0.83, -0.49]
**Subtotal (95% CI)**			**262**			**262**	**64.1%**	**-0.61 [-0.73, -0.49]**
Heterogeneity: Tau² = 0.00; Chi² = 2.28, df = 2 (P = 0.32); I² = 12%								
Test for overall effect: Z = 9.81 (P < 0.00001)								
**Total (95% CI)**			**337**			**337**	**100.0%**	**-0.49 [-0.69, -0.28]**
Heterogeneity: Tau² = 0.04; Chi² = 20.71, df = 4 (P = 0.0004); I² = 81%								
Test for overall effect: Z = 4.70 (P < 0.00001)								
Test for subgroup differences: Chi² = 17.05, df = 1 (P < 0.0001), I² = 94.1%								

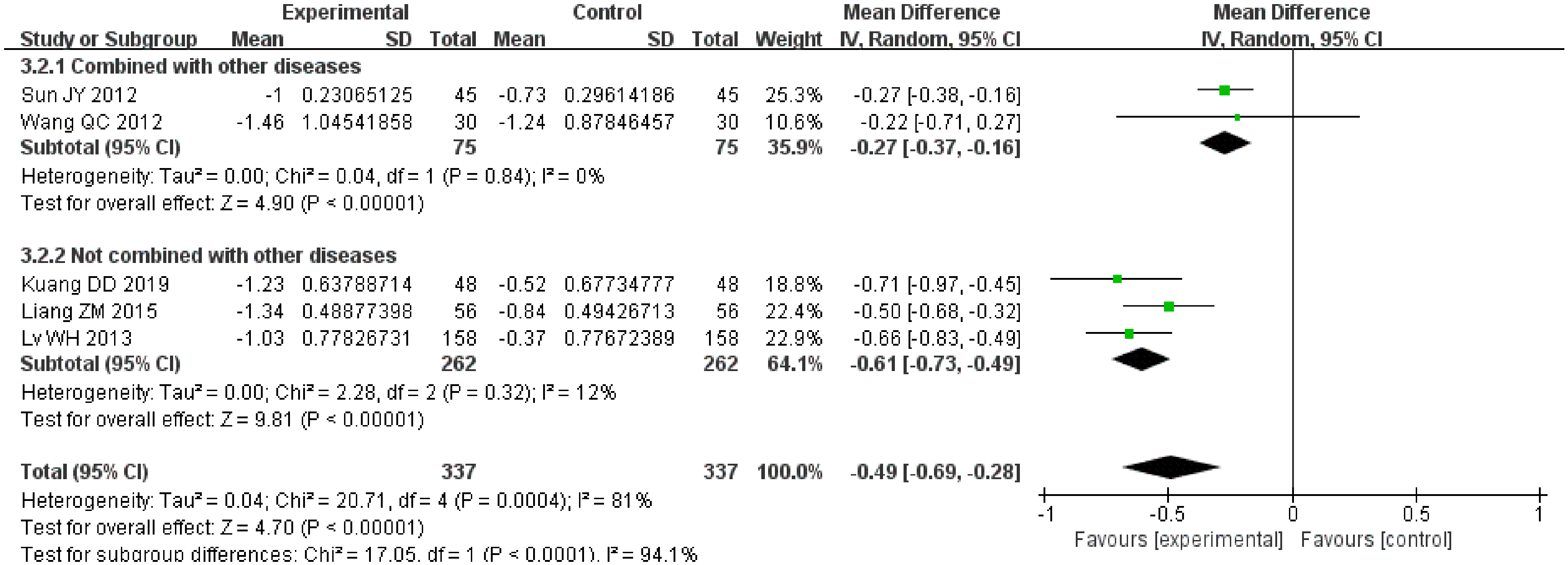

S8.3.4 TT+JZL subgroup analysis based on treatment duration (TG).

Study or Subgroup	Experimental Mean	Experimental SD	Experimental Total	Control Mean	Control SD	Control Total	Weight	Mean Difference IV, Random, 95% CI
**3.8.1 Duration ≥12**								
Zhang XL 2017	-1.27	0.61587336	50	-0.57	0.66550732	50	32.9%	-0.70 [-0.95, -0.45]
Zhu TW 2021	-1.49	0.62193247	40	-0.93	0.69346954	40	30.2%	-0.56 [-0.85, -0.27]
**Subtotal (95% CI)**			**90**			**90**	**63.2%**	**-0.64 [-0.83, -0.45]**
Heterogeneity: Tau² = 0.00; Chi² = 0.51, df = 1 (P = 0.47); I² = 0%								
Test for overall effect: Z = 6.61 (P < 0.00001)								
**3.8.2 Duration <12**								
Guo XP 2010	-1.48	0.75940766	34	-1.38	0.78083289	34	25.1%	-0.10 [-0.47, 0.27]
Hu XW 2015	-0.82	1.7351657	48	-0.3	1.72217885	50	11.8%	-0.52 [-1.20, 0.16]
**Subtotal (95% CI)**			**82**			**84**	**36.8%**	**-0.21 [-0.56, 0.15]**
Heterogeneity: Tau² = 0.01; Chi² = 1.12, df = 1 (P = 0.29); I² = 11%								
Test for overall effect: Z = 1.13 (P = 0.26)								
**Total (95% CI)**			**172**			**174**	**100.0%**	**-0.49 [-0.76, -0.21]**
Heterogeneity: Tau² = 0.04; Chi² = 7.10, df = 3 (P = 0.07); I² = 58%								
Test for overall effect: Z = 3.50 (P = 0.0005)								
Test for subgroup differences: Chi² = 4.40, df = 1 (P = 0.04), I² = 77.3%								

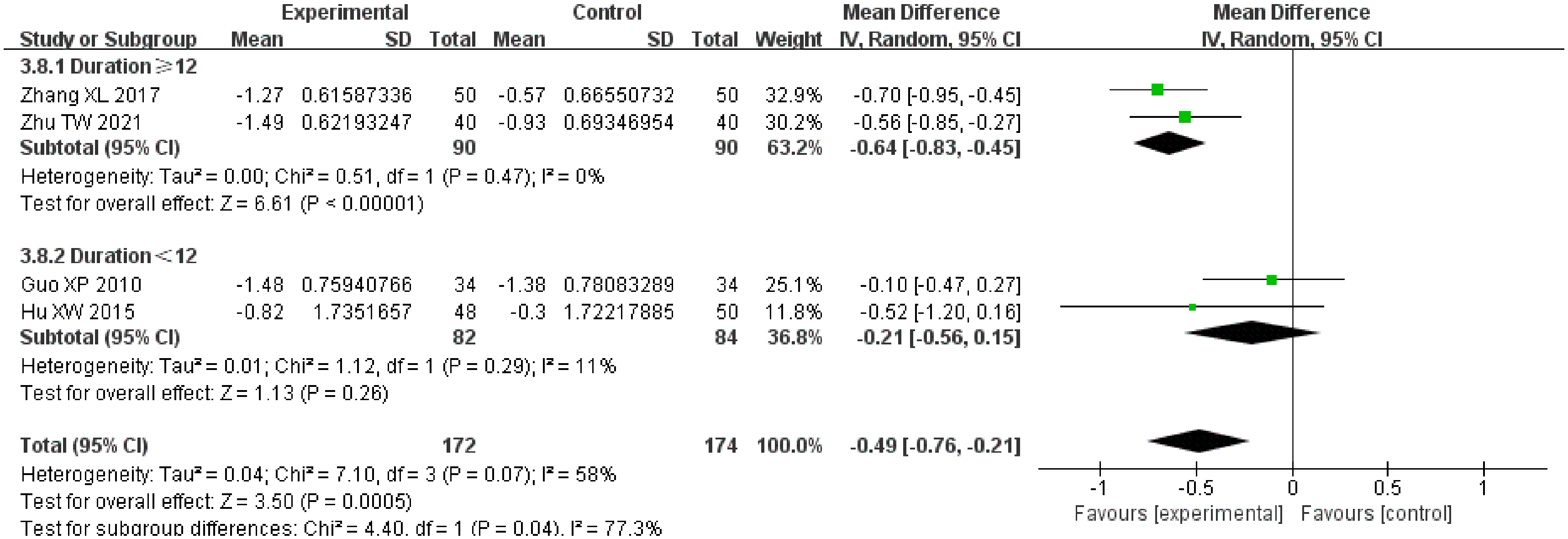

S8.3.5 TT+JZL subgroup analysis based on average age (TG).

Study or Subgroup	Experimental Mean	Experimental SD	Experimental Total	Control Mean	Control SD	Control Total	Weight	Mean Difference IV, Random, 95% CI
**3.6.1 AverageAge ≥50**								
Zhang XL 2017	-1.27	0.61587336	50	-0.57	0.66550732	50	32.9%	-0.70 [-0.95, -0.45]
Zhu TW 2021	-1.49	0.62193247	40	-0.93	0.69346954	40	30.2%	-0.56 [-0.85, -0.27]
**Subtotal (95% CI)**			**90**			**90**	**63.2%**	**-0.64 [-0.83, -0.45]**
Heterogeneity: Tau² = 0.00; Chi² = 0.51, df = 1 (P = 0.47); I² = 0%								
Test for overall effect: Z = 6.61 (P < 0.00001)								
**3.6.2 AverageAge <50**								
Guo XP 2010	-1.48	0.75940766	34	-1.38	0.78083289	34	25.1%	-0.10 [-0.47, 0.27]
Hu XW 2015	-0.82	1.7351657	48	-0.3	1.72217885	50	11.8%	-0.52 [-1.20, 0.16]
**Subtotal (95% CI)**			**82**			**84**	**36.8%**	**-0.21 [-0.56, 0.15]**
Heterogeneity: Tau² = 0.01; Chi² = 1.12, df = 1 (P = 0.29); I² = 11%								
Test for overall effect: Z = 1.13 (P = 0.26)								
**Total (95% CI)**			**172**			**174**	**100.0%**	**-0.49 [-0.76, -0.21]**
Heterogeneity: Tau² = 0.04; Chi² = 7.10, df = 3 (P = 0.07); I² = 58%								
Test for overall effect: Z = 3.50 (P = 0.0005)								
Test for subgroup differences: Chi² = 4.40, df = 1 (P = 0.04), I² = 77.3%								

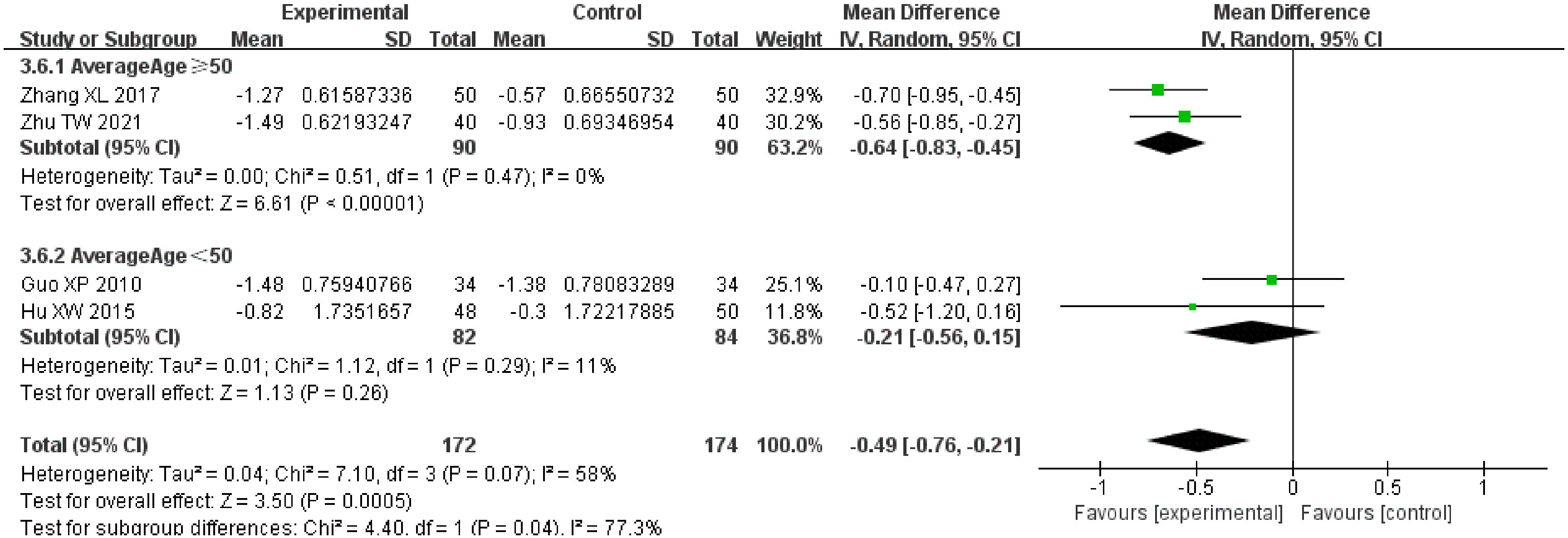

S8.3.6 TT+JZTM subgroup analysis based on sample size (TG).

Study or Subgroup	Experimental Mean	Experimental SD	Experimental Total	Control Mean	Control SD	Control Total	Weight	Mean Difference IV, Random, 95% CI
**3.11.1 SampleSize ≥90**								
Lu HM 2021	-1.28	1.54618886	46	-0.42	1.56144164	46	16.7%	-0.86 [-1.50, -0.22]
You HS 2022	-1.27	1.27996094	50	-0.38	1.23121891	50	20.6%	-0.89 [-1.38, -0.40]
**Subtotal (95% CI)**			**96**			**96**	**37.2%**	**-0.88 [-1.27, -0.49]**
Heterogeneity: Tau² = 0.00; Chi² = 0.01, df = 1 (P = 0.94); I² = 0%								
Test for overall effect: Z = 4.43 (P < 0.00001)								
**3.11.2 SampleSize <90**								
Liu FG 2011	-0.43	0.95582425	30	-0.25	1.27432335	30	18.4%	-0.18 [-0.75, 0.39]
Wang H 2019	-0.97	0.96994845	15	-0.4	0.90537285	15	15.8%	-0.57 [-1.24, 0.10]
Xiao B 2014	-1.16	0.53674948	40	-1.05	0.46936127	40	28.6%	-0.11 [-0.33, 0.11]
**Subtotal (95% CI)**			**85**			**85**	**62.8%**	**-0.16 [-0.35, 0.04]**
Heterogeneity: Tau² = 0.00; Chi² = 1.63, df = 2 (P = 0.44); I² = 0%								
Test for overall effect: Z = 1.57 (P = 0.12)								
**Total (95% CI)**			**181**			**181**	**100.0%**	**-0.48 [-0.86, -0.10]**
Heterogeneity: Tau² = 0.12; Chi² = 12.13, df = 4 (P = 0.02); I² = 67%								
Test for overall effect: Z = 2.51 (P = 0.01)								
Test for subgroup differences: Chi² = 10.50, df = 1 (P = 0.001), I² = 90.5%								

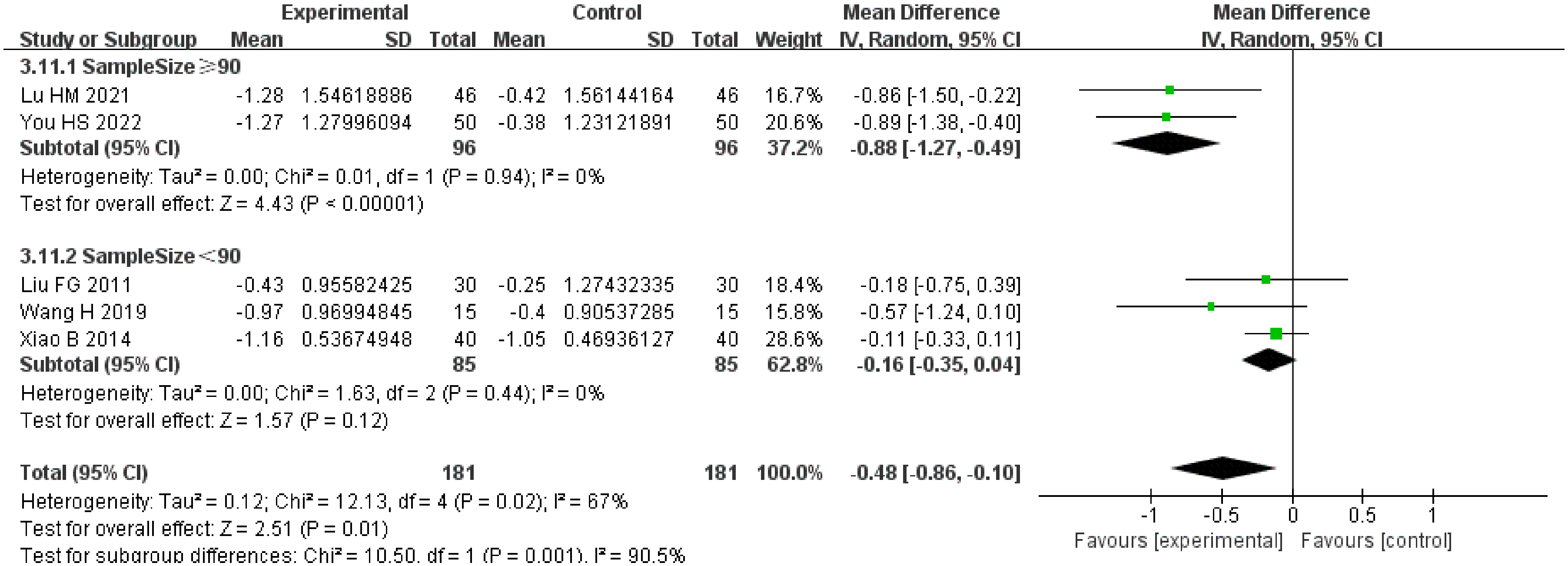

S8.3.7 TT+JZTM subgroup analysis based on different TT (TG).

Study or Subgroup	Experimental Mean	Experimental SD	Experimental Total	Control Mean	Control SD	Control Total	Weight	Mean Difference IV, Random, 95% CI
**3.25.1 Atorvastatin**								
Liu FG 2011	-0.43	0.95582425	30	-0.25	1.27432335	30	18.4%	-0.18 [-0.75, 0.39]
Xiao B 2014	-1.16	0.53674948	40	-1.05	0.46936127	40	28.6%	-0.11 [-0.33, 0.11]
**Subtotal (95% CI)**			**70**			**70**	**47.0%**	**-0.12 [-0.33, 0.09]**
Heterogeneity: Tau² = 0.00; Chi² = 0.05, df = 1 (P = 0.82); I² = 0%								
Test for overall effect: Z = 1.13 (P = 0.26)								
**3.25.2 Simvastatin**								
Lu HM 2021	-1.28	1.54618886	46	-0.42	1.56144164	46	16.7%	-0.86 [-1.50, -0.22]
Wang H 2019	-0.97	0.96994845	15	-0.4	0.90537285	15	15.8%	-0.57 [-1.24, 0.10]
You HS 2022	-1.27	1.27996094	50	-0.38	1.23121891	50	20.6%	-0.89 [-1.38, -0.40]
**Subtotal (95% CI)**			**111**			**111**	**53.0%**	**-0.80 [-1.14, -0.46]**
Heterogeneity: Tau² = 0.00; Chi² = 0.61, df = 2 (P = 0.74); I² = 0%								
Test for overall effect: Z = 4.66 (P < 0.00001)								
**Total (95% CI)**			**181**			**181**	**100.0%**	**-0.48 [-0.86, -0.10]**
Heterogeneity: Tau² = 0.12; Chi² = 12.13, df = 4 (P = 0.02); I² = 67%								
Test for overall effect: Z = 2.51 (P = 0.01)								
Test for subgroup differences: Chi² = 11.47, df = 1 (P = 0.0007), I² = 91.3%								

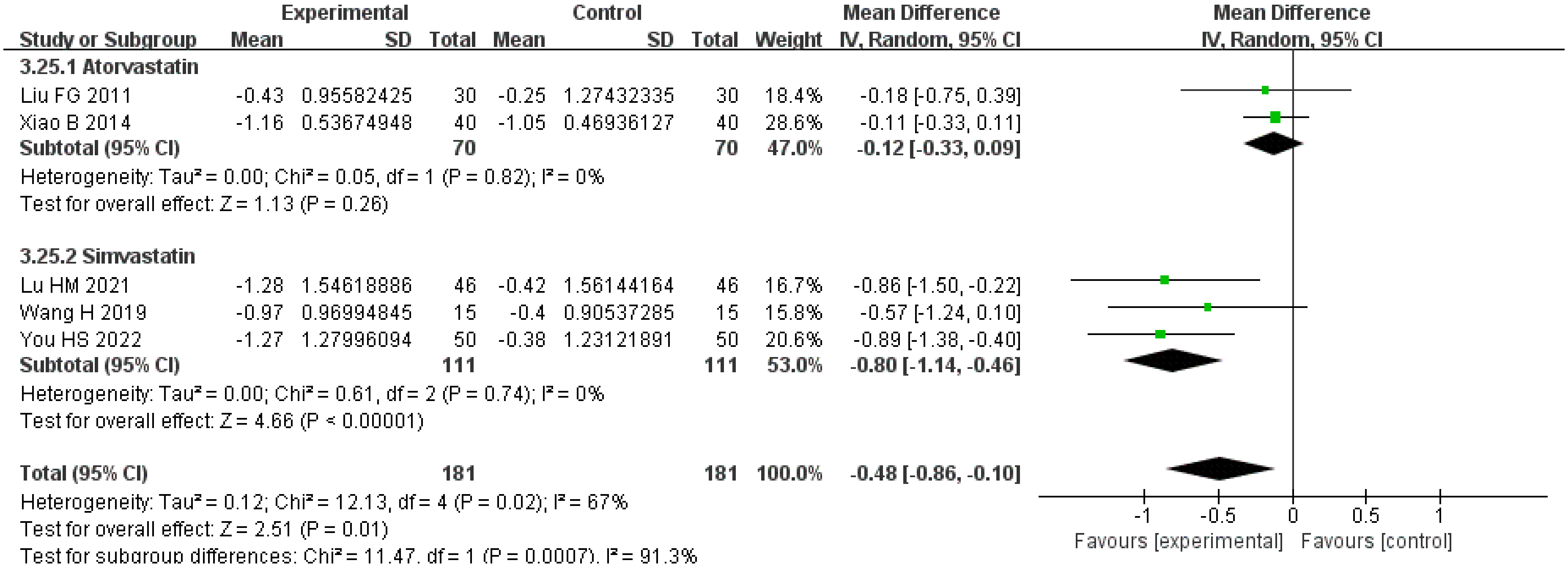

S8.4 HDL-C

	TT+DTJZ			TT+JZL			TT+JZTL		
Subgroup	Criteria	MD[95% CI]	Subgroup Difference p-value	Criteria	MD[95% CI]	Subgroup Difference p-value	Criteria	MD[95% CI]	Subgroup Difference p-value
Sample Size	≥96	0.50 [0.28, 0.71]	**0.002**	≥90	0.49 [-0.15, 1.12]	0.59	≥115	0.14 [-0.11, 0.38]	0.18
	<96	0.12 [0.04, 0.20]		<90	0.31 [0.21, 0.40]		<115	0.40 [0.10, 0.69]	
Duration	≥12	0.24 [0.12, 0.35]	0.7	≥12	0.56 [0.07, 1.05]	0.18			
	<12	0.27 [0.22, 0.31]		<12	0.22 [0.11, 0.33]				
Average Age	≥60	0.21 [-0.09, 0.52]	0.3	≥55	0.56 [0.07, 1.05]	0.18	≥54	0.14 [-0.11, 0.38]	0.18
	<60	0.42 [0.17, 0.68]		<55	0.22 [0.11, 0.33]		<54	0.40 [0.10, 0.69]	
Combined with other diseases	Yes	0.12 [0.04, 0.20]	**0.002**	Yes	0.25 [0.10, 0.40]	0.3			
	No	0.50 [0.28, 0.71]		No	0.54 [0.01, 1.07]				
Treatment with different TT	Atorvastatin	0.45 [-0.33, 1.22]	0.7						
	Simvastatin	0.29 [0.13, 0.46]							

	TT+JZTM			TT+PS			TT+SLXMK		
Subgroup	Criteria	MD[95% CI]	Subgroup Difference p-value	Criteria	MD[95% CI]	Subgroup Difference p-value	Criteria	MD[95% CI]	Subgroup Difference p-value
Sample Size	≥90	0.54 [0.45, 0.62]	**0.01**	≥80	0.32 [-0.00, 0.65]	0.89	≥100	0.32 [-0.03, 0.67]	0.37
	<90	0.18 [-0.08, 0.44]		<80	0.29 [-0.01, 0.60]		<100	0.12 [-0.15, 0.39]	
Duration	≥6	0.49 [0.05, 0.92]	0.55	≥12	0.18 [-0.10, 0.47]	0.34	≥12	0.12 [-0.15, 0.39]	0.37
	<6	0.27 [-0.30, 0.84]		<12	0.40 [0.05, 0.75]		<12	0.32 [-0.03, 0.67]	
Average Age	≥60	0.49 [0.05, 0.92]	0.55	≥60	0.29 [-0.14, 0.73]	0.91	≥60	0.24 [-0.31, 0.79]	0.91
	<60	0.27 [-0.30, 0.84]		<60	0.32 [0.15, 0.49]		<60	0.21 [0.04, 0.37]	
Combined with other diseases	Yes	0.30 [-0.20, 0.80]	0.62	Yes	0.40 [0.02, 0.79]	0.49			
	No	0.48 [-0.01, 0.97]		No	0.24 [-0.02, 0.50]				
Treatment with different TT	Atorvastatin	0.03 [-0.04, 0.10]	**0.00001**	Atorvastatin	0.17 [-0.12, 0.46]	0.09			
	Simvastatin	0.58 [0.42, 0.74]		Rosuvastatin	0.54 [0.22, 0.86]				

	TT+XZK			TT+XZT		
Subgroup	Criteria	MD[95% CI]	Subgroup Difference p-value	Criteria	MD[95% CI]	Subgroup Difference p-value
Sample Size	≥100	0.19 [0.15, 0.22]	0.62	≥100	0.30 [0.20, 0.40]	0.22
	<100	0.21 [0.13, 0.29]		<100	0.19 [0.03, 0.34]	

Duration	≥12	0.22 [0.16, 0.28]	0.24	≥12	0.28 [0.16, 0.39]	0.95
	<12	0.18 [0.13, 0.22]		<12	0.27 [0.02, 0.51]	
Average Age	≥60	0.14 [0.11, 0.18]	**0.0001**	≥52	0.29 [0.15, 0.42]	0.79
	<60	0.29 [0.22, 0.35]		<52	0.26 [0.10, 0.41]	
Combined with other diseases	Yes	0.23 [0.14, 0.32]	0.38			
	No	0.18 [0.15, 0.22]				
Treatment with different TT	Atorvastatin	0.23 [0.17, 0.28]	0.05			
	Simvastatin	0.13 [0.03, 0.22]				
	Pitavastatin	0.37 [0.17, 0.56]				

S8.4.1 TT+DTJZ subgroup analysis based on sample size (HDL-C).

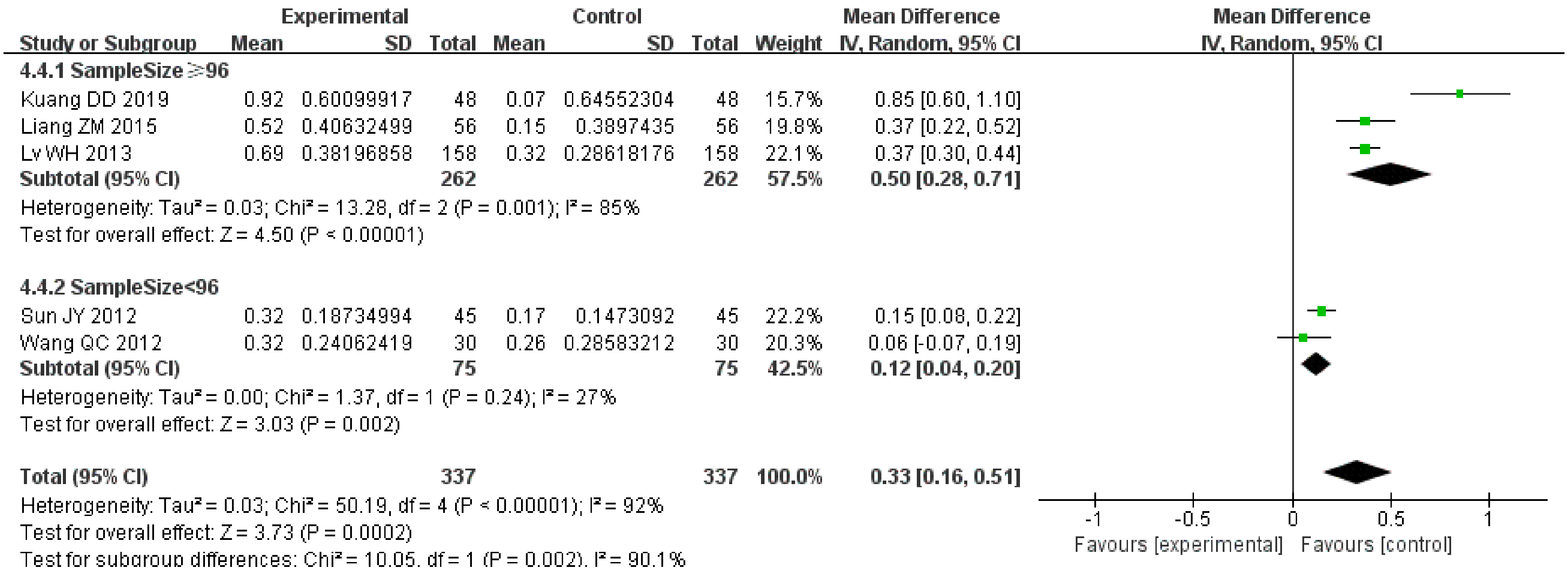

S8.4.2 TT+DTJZ subgroup analysis based on whether or not combined with other diseases (HDL-C).

Study or Subgroup	Experimental Mean	Experimental SD	Experimental Total	Control Mean	Control SD	Control Total	Weight	Mean Difference IV, Random, 95% CI
**4.6.1 Combined with other diseases**								
Sun JY 2012	0.32	0.18734994	45	0.17	0.1473092	45	22.2%	0.15 [0.08, 0.22]
Wang QC 2012	0.32	0.24062419	30	0.26	0.28583212	30	20.3%	0.06 [-0.07, 0.19]
**Subtotal (95% CI)**			**75**			**75**	**42.5%**	**0.12 [0.04, 0.20]**
Heterogeneity: Tau² = 0.00; Chi² = 1.37, df = 1 (P = 0.24); I² = 27%								
Test for overall effect: Z = 3.03 (P = 0.002)								
**4.6.2 Not combined with other diseases**								
Kuang DD 2019	0.92	0.60099917	48	0.07	0.64552304	48	15.7%	0.85 [0.60, 1.10]
Liang ZM 2015	0.52	0.40632499	56	0.15	0.3897435	56	19.8%	0.37 [0.22, 0.52]
Lv WH 2013	0.69	0.38196858	158	0.32	0.28618176	158	22.1%	0.37 [0.30, 0.44]
**Subtotal (95% CI)**			**262**			**262**	**57.5%**	**0.50 [0.28, 0.71]**
Heterogeneity: Tau² = 0.03; Chi² = 13.28, df = 2 (P = 0.001); I² = 85%								
Test for overall effect: Z = 4.50 (P < 0.00001)								
**Total (95% CI)**			**337**			**337**	**100.0%**	**0.33 [0.16, 0.51]**
Heterogeneity: Tau² = 0.03; Chi² = 50.19, df = 4 (P < 0.00001); I² = 92%								
Test for overall effect: Z = 3.73 (P = 0.0002)								
Test for subgroup differences: Chi² = 10.05, df = 1 (P = 0.002), I² = 90.1%								

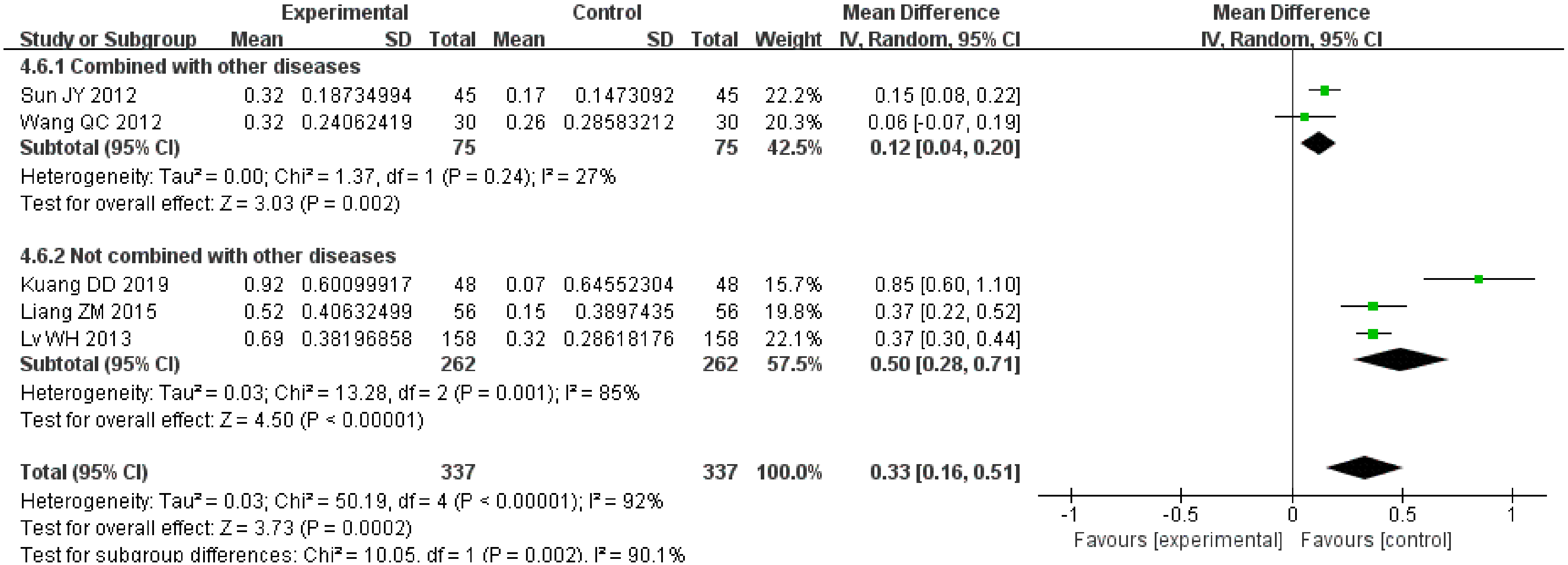

S8.4.3 TT+JZTM subgroup analysis based on sample size (HDL-C).

Study or Subgroup	Experimental Mean	Experimental SD	Experimental Total	Control Mean	Control SD	Control Total	Weight	Mean Difference IV, Random, 95% CI
**4.16.1 SampleSize≥90**								
Lu HM 2021	0.88	0.27838822	46	0.32	0.27055498	46	22.1%	0.56 [0.45, 0.67]
You HS 2022	0.81	0.27784888	50	0.3	0.33600595	50	21.9%	0.51 [0.39, 0.63]
**Subtotal (95% CI)**			**96**			**96**	**44.0%**	**0.54 [0.45, 0.62]**
Heterogeneity: Tau² = 0.00; Chi² = 0.35, df = 1 (P = 0.55); I² = 0%								
Test for overall effect: Z = 12.80 (P < 0.00001)								
**4.16.2 SampleSize<90**								
Liu FG 2011	0.01	0.23895606	30	0.03	0.24062419	30	21.9%	-0.02 [-0.14, 0.10]
Wang H 2019	1.31	0.7192357	15	0.11	0.91	15	11.7%	1.20 [0.61, 1.79]
Xiao B 2014	0.26	0.22338308	40	0.21	0.14798649	40	22.4%	0.05 [-0.03, 0.13]
**Subtotal (95% CI)**			**85**			**85**	**56.0%**	**0.18 [-0.08, 0.44]**
Heterogeneity: Tau² = 0.04; Chi² = 15.99, df = 2 (P = 0.0003); I² = 87%								
Test for overall effect: Z = 1.35 (P = 0.18)								
**Total (95% CI)**			**181**			**181**	**100.0%**	**0.38 [0.10, 0.67]**
Heterogeneity: Tau² = 0.09; Chi² = 98.43, df = 4 (P < 0.00001); I² = 96%								
Test for overall effect: Z = 2.62 (P = 0.009)								
Test for subgroup differences: Chi² = 6.72, df = 1 (P = 0.010), I² = 85.1%								

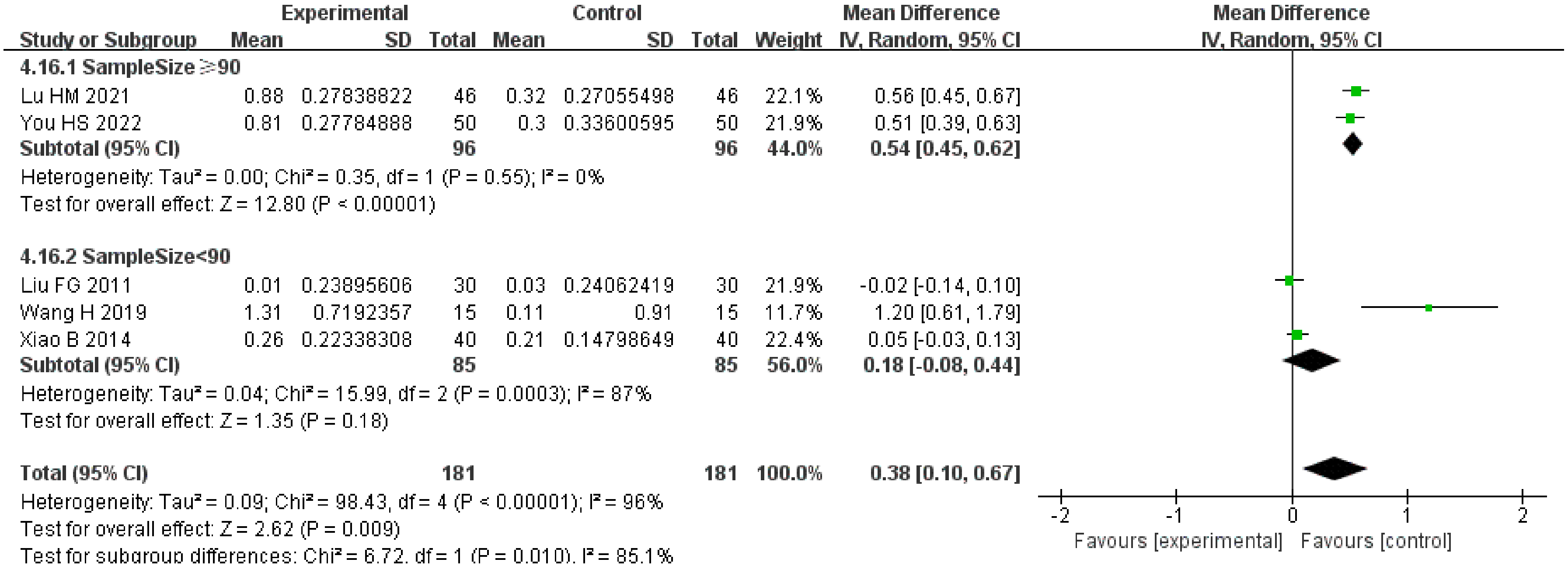

S8.4.4 TT+JZTM subgroup analysis based on different TT (HDL-C).

	Experimental			Control				Mean Difference
**Study or Subgroup**	**Mean**	**SD**	**Total**	**Mean**	**SD**	**Total**	**Weight**	**IV, Random, 95% CI**
**4.14.1 Atorvastatin**								
Liu FG 2011	0.01	0.23895606	30	0.03	0.24062419	30	21.9%	-0.02 [-0.14, 0.10]
Xiao B 2014	0.26	0.22338308	40	0.21	0.14798649	40	22.4%	0.05 [-0.03, 0.13]
**Subtotal (95% CI)**			**70**			**70**	**44.3%**	**0.03 [-0.04, 0.10]**
Heterogeneity: Tau² = 0.00; Chi² = 0.87, df = 1 (P = 0.35); I² = 0%								
Test for overall effect: Z = 0.79 (P = 0.43)								
**4.14.2 Simvastatin**								
Lu HM 2021	0.88	0.27838822	46	0.32	0.27055498	46	22.1%	0.56 [0.45, 0.67]
Wang H 2019	1.31	0.7192357	15	0.11	0.91	15	11.7%	1.20 [0.61, 1.79]
You HS 2022	0.81	0.27784888	50	0.3	0.33600595	50	21.9%	0.51 [0.39, 0.63]
**Subtotal (95% CI)**			**111**			**111**	**55.7%**	**0.58 [0.42, 0.74]**
Heterogeneity: Tau² = 0.01; Chi² = 5.16, df = 2 (P = 0.08); I² = 61%								
Test for overall effect: Z = 7.13 (P < 0.00001)								
**Total (95% CI)**			**181**			**181**	**100.0%**	**0.38 [0.10, 0.67]**

Heterogeneity: Tau² = 0.09; Chi² = 98.43, df = 4 (P < 0.00001); I² = 96%
Test for overall effect: Z = 2.62 (P = 0.009)
Test for subgroup differences: Chi² = 38.92, df = 1 (P < 0.00001), I² = 97.4%

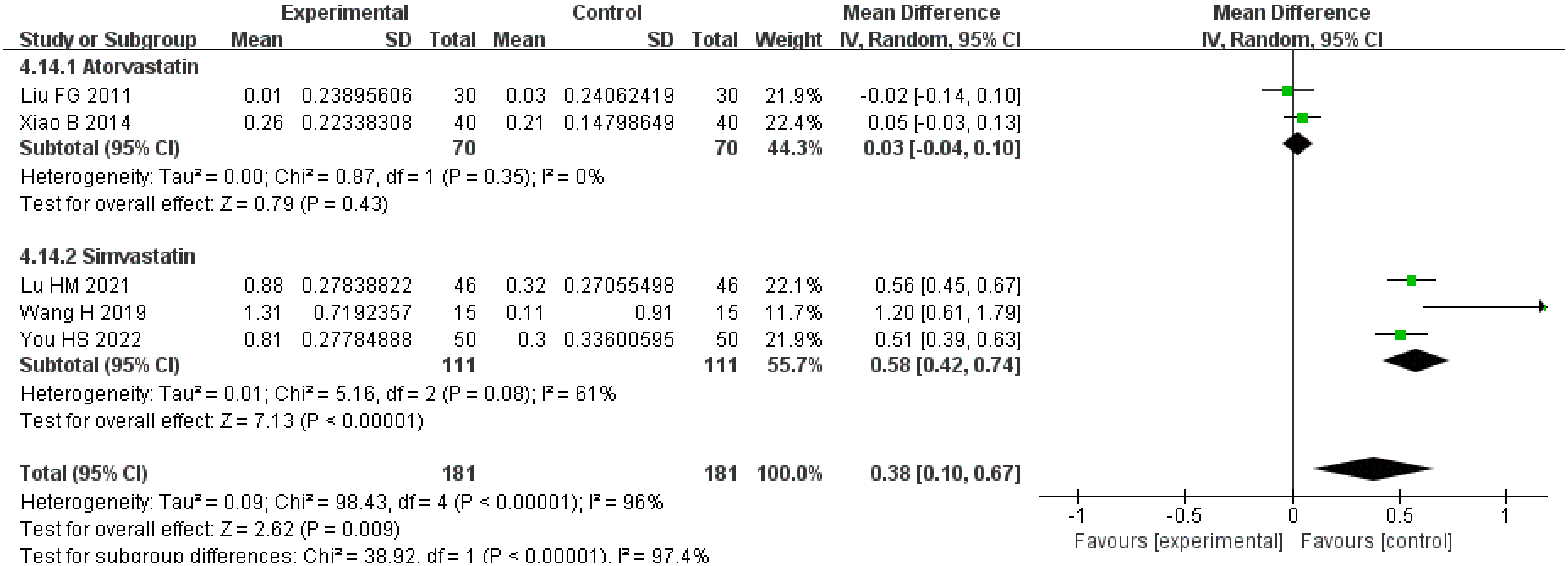

S8.4.5 TT+XZK subgroup analysis based on average age (HDL-C).

Study or Subgroup	Experimental Mean	Experimental SD	Experimental Total	Control Mean	Control SD	Control Total	Weight	Mean Difference IV, Random, 95% CI
**4.27.1 AverageAge ≥60**								
Chen HY 2014	0.4	0.15524175	42	0.29	0.13114877	42	6.0%	0.11 [0.05, 0.17]
Fu YJ 2017	0.2	0.30512293	75	0.08	0.20518284	75	5.3%	0.12 [0.04, 0.20]
Jiang CH 2012	0.36	0.15394804	85	0.2	0.14525839	85	6.4%	0.16 [0.12, 0.20]
Jiang F 2013	0.42	0.30116441	28	0.22	0.30512293	28	3.4%	0.20 [0.04, 0.36]
Li RG 2023	0.45	0.46486557	100	0.18	0.46032597	100	4.1%	0.27 [0.14, 0.40]
Liu YS 2022	0.57	0.16522712	59	0.45	0.19924859	59	5.8%	0.12 [0.05, 0.19]
Ma JB 2015	0.16	0.22715633	40	0.15	0.24062419	40	4.8%	0.01 [-0.09, 0.11]
Qiang SC 2023	0.31	0.10148892	90	0.13	0.09165151	90	6.7%	0.18 [0.15, 0.21]
Shi C 2018	0.26	0.34394767	62	0.05	0.27221315	62	4.6%	0.21 [0.10, 0.32]
Sun XJ 2018	0.11	0.18734994	48	0.04	0.19313208	48	5.6%	0.07 [-0.01, 0.15]
Wei GR 2011	0.018	0.85378393	38	0.013	0.15418171	38	1.6%	0.00 [-0.27, 0.28]
Xu XC 2024	0.45	0.52373658	50	0.17	0.33151169	50	3.1%	0.28 [0.11, 0.45]
**Subtotal (95% CI)**			**717**			**717**	**57.3%**	**0.14 [0.11, 0.18]**
Heterogeneity: Tau² = 0.00; Chi² = 26.86, df = 11 (P = 0.005); I² = 59%								
Test for overall effect: Z = 7.77 (P < 0.00001)								
**4.27.2 AverageAge<60**								
Hu GY 2015	0.67	0.44170126	60	0.29	0.84184322	60	2.0%	0.38 [0.14, 0.62]
Qu J 2020	0.6	0.42508823	39	0.34	0.31953091	39	3.2%	0.26 [0.09, 0.43]
Tian L 2022	0.86	0.23515952	30	0.4	0.24269322	30	4.3%	0.46 [0.34, 0.58]
Wang Z 2012	0.29	0.13453624	39	0.11	0.12288206	39	6.1%	0.18 [0.12, 0.24]
Wang ZZ 2013	0.51	0.14177447	27	0.16	0.09539392	27	5.9%	0.35 [0.29, 0.41]
Ye JG 2021	0.5	0.34641016	60	0.3	0.26457513	60	4.6%	0.20 [0.09, 0.31]
Yu Q 2021	0.58	0.30805844	38	0.15	0.36373067	38	3.5%	0.43 [0.28, 0.58]
Zhou L 2024	0.56	0.45431267	41	0.37	0.40706265	41	2.8%	0.19 [0.00, 0.38]
Zhou M 2017	0.48	0.47465777	244	0.2	0.4613025	244	5.4%	0.28 [0.20, 0.36]
Zhou Z 2010	0.34	0.2116601	39	0.11	0.20952327	39	5.1%	0.23 [0.14, 0.32]
**Subtotal (95% CI)**			**617**			**617**	**42.7%**	**0.29 [0.22, 0.35]**
Heterogeneity: Tau² = 0.01; Chi² = 33.01, df = 9 (P = 0.0001); I² = 73%								
Test for overall effect: Z = 8.77 (P < 0.00001)								
**Total (95% CI)**			**1334**			**1334**	**100.0%**	**0.21 [0.17, 0.25]**
Heterogeneity: Tau² = 0.01; Chi² = 102.17, df = 21 (P < 0.00001); I² = 79%								
Test for overall effect: Z = 9.95 (P < 0.00001)								
Test for subgroup differences: Chi² = 15.17, df = 1 (P < 0.0001), I² = 93.4%								

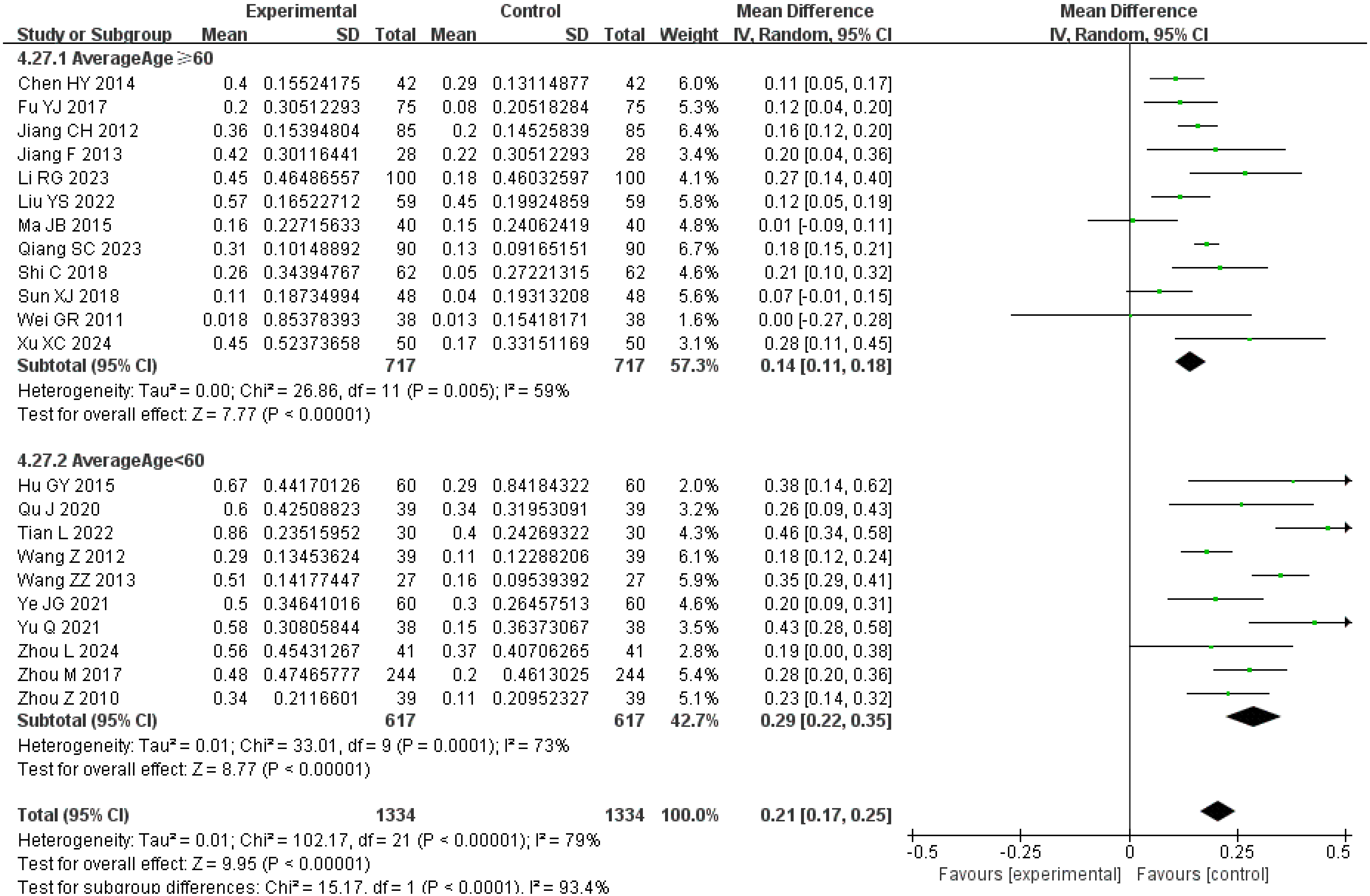

S8.5 LDL-C

	TT+DTJZ			TT+JZL			TT+PS		
Subgroup	Criteria	MD[95% CI]	Subgroup Difference p-value	Criteria	MD[95% CI]	Subgroup Difference p-value	Criteria	MD[95% CI]	Subgroup Difference p-value
Sample Size	≥96	-0.90 [-1.03, -0.76]	**0.00001**	≥90	-0.78 [-1.16, -0.40]	0.26	≥80	-0.03 [-0.24, 0.18]	0.31
	<96	-0.26 [-0.43, -0.10]		<90	-0.50 [-0.80, -0.21]		<80	-0.16 [-0.30, -0.02]	
Duration	≥12	-0.65 [-1.30, -0.00]	0.85	≥12	-0.67 [-1.29, -0.05]	0.85	≥12	-0.05 [-0.16, 0.05]	0.25
	<12	-0.56 [-1.17, 0.05]		<12	-0.61 [-0.85, -0.37]		<12	-0.19 [-0.39, 0.02]	
Average Age	≥60	-0.61 [-1.16, -0.07]	0.98	≥55	-0.67 [-1.29, -0.05]	0.85	≥60	-0.07 [-0.23, 0.10]	0.46
	<60	-0.60 [-1.31, 0.10]		<55	-0.61 [-0.85, -0.37]		<60	-0.16 [-0.35, 0.02]	
Combined with other diseases	Yes	-0.26 [-0.43, -0.10]	**0.00001**	Yes	-0.49 [-0.74, -0.23]	0.11	Yes	-0.04 [-0.15, 0.06]	0.15
	No	-0.90 [-1.03, -0.76]		No	-0.82 [-1.14, -0.50]		No	-0.21 [-0.41, -0.01]	
Treatment with different TT	Atorvastatin	-0.65 [-1.30, -0.00]	0.85				Atorvastatin	-0.20 [-0.45, 0.05]	0.21
	Simvastatin	-0.56 [-1.17, 0.05]					Rosuvastatin	-0.03 [-0.13, 0.08]	

	TT+XZK			TT+XZT		
Subgroup	Criteria	MD[95% CI]	Subgroup Difference p-value	Criteria	MD[95% CI]	Subgroup Difference p-value
Sample Size	≥100	-0.40 [-0.59, -0.22]	0.71	≥100	-0.46 [-0.64, -0.27]	0.42
	<100	-0.35 [-0.54, -0.16]		<100	-0.72 [-1.33, -0.11]	
Duration	≥12	-0.40 [-0.55, -0.26]	0.64	≥12	-0.52 [-0.86, -0.18]	0.92
	<12	-0.34 [-0.56, -0.12]		<12	-0.54 [-0.68, -0.39]	
Average Age	≥60	-0.35 [-0.55, -0.16]	0.66	≥52	-0.57 [-0.95, -0.19]	0.55
	<60	-0.41 [-0.58, -0.24]		<52	-0.43 [-0.68, -0.17]	
Combined with other diseases	Yes	-0.34 [-0.53, -0.16]	0.65			
	No	-0.40 [-0.57, -0.23]				
Treatment with different TT	Atorvastatin	-0.41 [-0.60, -0.23]	**0.006**			
	Simvastatin	-0.04 [-0.26, 0.17]				
	Pitavastatin	-0.51 [-0.72, -0.29]				

S8.5.1 TT+DTJZ subgroup analysis based on sample size (LDL-C)

Study or Subgroup	Experimental Mean	Experimental SD	Experimental Total	Control Mean	Control SD	Control Total	Weight	Mean Difference IV, Random, 95% CI
**5.4.1 SampleSize ≥96**								
Kuang DD 2019	-4.99	0.70632854	48	-4.02	0.63379808	48	24.5%	-0.97 [-1.24, -0.70]
Liang ZM 2015	-2.64	0.32908965	56	-1.77	0.49568135	56	26.7%	-0.87 [-1.03, -0.71]
**Subtotal (95% CI)**			**104**			**104**	**51.2%**	**-0.90 [-1.03, -0.76]**
Heterogeneity: Tau² = 0.00; Chi² = 0.40, df = 1 (P = 0.53); I² = 0%								
Test for overall effect: Z = 13.02 (P < 0.00001)								
**5.4.2 SampleSize<96**								
Sun JY 2012	-0.61	0.42567593	45	-0.36	0.48445846	45	26.2%	-0.25 [-0.44, -0.06]
Wang QC 2012	-2.07	0.71630999	30	-1.76	0.66121101	30	22.6%	-0.31 [-0.66, 0.04]
**Subtotal (95% CI)**			**75**			**75**	**48.8%**	**-0.26 [-0.43, -0.10]**
Heterogeneity: Tau² = 0.00; Chi² = 0.09, df = 1 (P = 0.77); I² = 0%								
Test for overall effect: Z = 3.12 (P = 0.002)								
**Total (95% CI)**			**179**			**179**	**100.0%**	**-0.61 [-0.98, -0.23]**
Heterogeneity: Tau² = 0.13; Chi² = 34.06, df = 3 (P < 0.00001); I² = 91%								
Test for overall effect: Z = 3.15 (P = 0.002)								
Test for subgroup differences: Chi² = 33.58, df = 1 (P < 0.00001), I² = 97.0%								

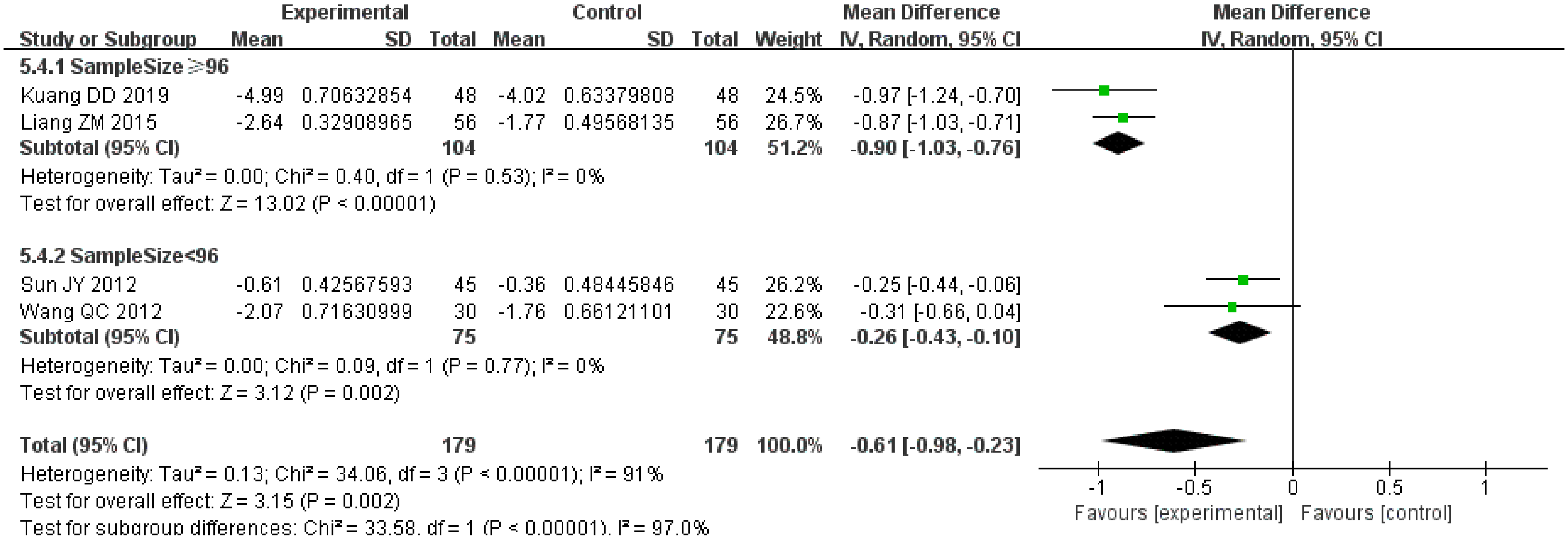

S8.5.2 TT+DTJZ subgroup analysis based on whether or not combined with other diseases (LDL-C)

Study or Subgroup	Experimental Mean	Experimental SD	Experimental Total	Control Mean	Control SD	Control Total	Weight	Mean Difference IV, Random, 95% CI
**5.6.1 Combined with other diseases**								
Sun JY 2012	-0.61	0.42567593	45	-0.36	0.48445846	45	26.2%	-0.25 [-0.44, -0.06]
Wang QC 2012	-2.07	0.71630999	30	-1.76	0.66121101	30	22.6%	-0.31 [-0.66, 0.04]
**Subtotal (95% CI)**			**75**			**75**	**48.8%**	**-0.26 [-0.43, -0.10]**
Heterogeneity: Tau² = 0.00; Chi² = 0.09, df = 1 (P = 0.77); I² = 0%								
Test for overall effect: Z = 3.12 (P = 0.002)								
**5.6.2 Not combined with other diseases**								
Kuang DD 2019	-4.99	0.70632854	48	-4.02	0.63379808	48	24.5%	-0.97 [-1.24, -0.70]
Liang ZM 2015	-2.64	0.32908965	56	-1.77	0.49568135	56	26.7%	-0.87 [-1.03, -0.71]
**Subtotal (95% CI)**			**104**			**104**	**51.2%**	**-0.90 [-1.03, -0.76]**
Heterogeneity: Tau² = 0.00; Chi² = 0.40, df = 1 (P = 0.53); I² = 0%								
Test for overall effect: Z = 13.02 (P < 0.00001)								
**Total (95% CI)**			**179**			**179**	**100.0%**	**-0.61 [-0.98, -0.23]**
Heterogeneity: Tau² = 0.13; Chi² = 34.06, df = 3 (P < 0.00001); I² = 91%								
Test for overall effect: Z = 3.15 (P = 0.002)								
Test for subgroup differences: Chi² = 33.58, df = 1 (P < 0.00001), I² = 97.0%								

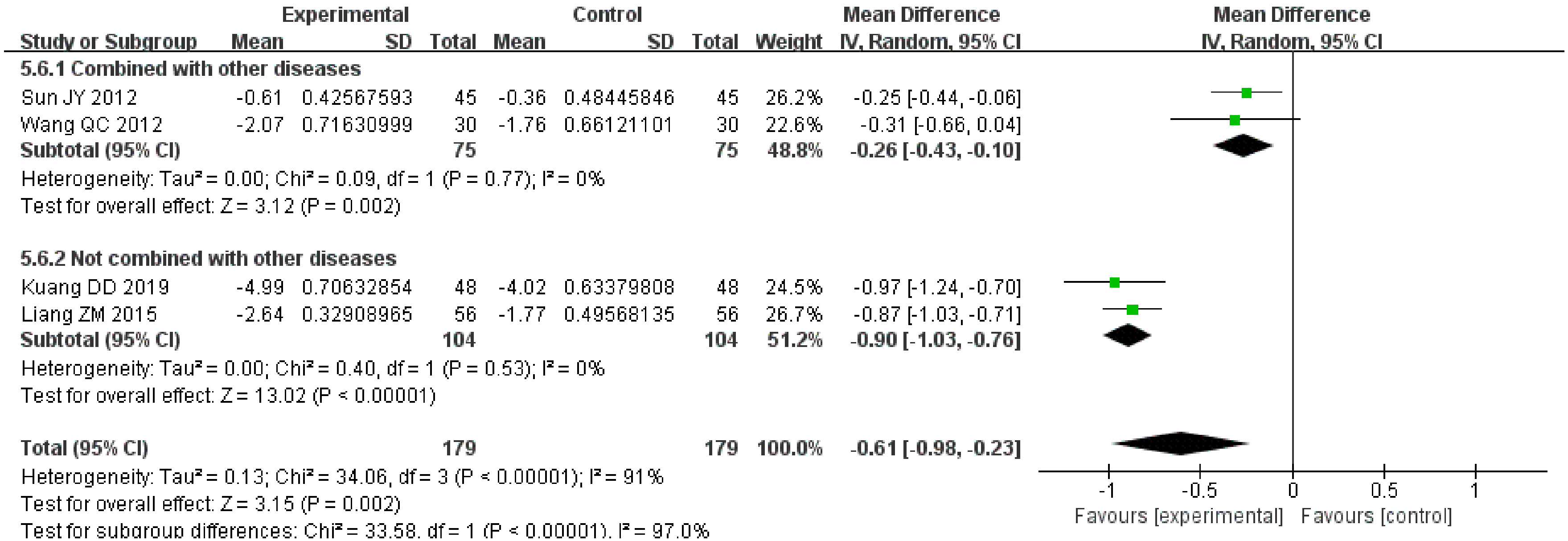

S8.5.3 TT+XZK subgroup analysis based on different TT (LDL-C)

Study or Subgroup	Experimental Mean	Experimental SD	Experimental Total	Control Mean	Control SD	Control Total	Weight	Mean Difference IV, Random, 95% CI
**5.12.1 Atorvastatin**								
Chen HY 2014	-4.03	0.47947888	42	-3.59	0.4596738	42	6.0%	-0.44 [-0.64, -0.24]
Fu YJ 2017	-0.9	0.52915026	75	-0.5	0.55677644	75	6.2%	-0.40 [-0.57, -0.23]
Jiang F 2013	-1.05	0.6780118	28	-1.12	0.67357256	28	4.7%	0.07 [-0.28, 0.42]
Li RG 2023	-0.54	0.81184974	100	-0.27	0.78076885	100	5.9%	-0.27 [-0.49, -0.05]
Liu YS 2022	-1.31	0.11357817	59	-1.29	0.14933184	59	6.9%	-0.02 [-0.07, 0.03]
Wang Z 2012	-1.33	0.47507894	39	-0.84	0.3207803	39	6.2%	-0.49 [-0.67, -0.31]
Wang ZZ 2013	-1.54	0.42673177	27	-0.71	0.44305756	27	5.8%	-0.83 [-1.06, -0.60]
Xu XC 2024	-3.28	0.87068938	50	-2.13	0.79642953	50	4.9%	-1.15 [-1.48, -0.82]
Ye JG 2021	-1.6	1.22882057	60	-1.1	1.30766968	60	3.9%	-0.50 [-0.95, -0.05]
Yu Q 2021	-0.57	0.5850641	38	-0.27	0.61991935	38	5.4%	-0.30 [-0.57, -0.03]
Zhou L 2024	-1.34	0.56320511	41	-0.92	0.59253692	41	5.6%	-0.42 [-0.67, -0.17]
Zhou M 2017	-0.55	0.79542442	244	-0.27	0.78172885	244	6.5%	-0.28 [-0.42, -0.14]
**Subtotal (95% CI)**			**803**			**803**	**68.1%**	**-0.41 [-0.60, -0.23]**
Heterogeneity: Tau² = 0.09; Chi² = 140.51, df = 11 (P < 0.00001); I² = 92%								
Test for overall effect: Z = 4.36 (P < 0.0001)								
**5.12.2 Simvastatin**								
Jiang CH 2012	-0.81	0.35383612	85	-0.57	0.35594944	85	6.7%	-0.24 [-0.35, -0.13]
Ma JB 2015	-1.03	0.50764161	40	-1.08	0.4932545	40	5.9%	0.05 [-0.17, 0.27]
Wei GR 2011	-0.836	1.16808861	38	-0.827	1.18584822	38	3.3%	-0.01 [-0.54, 0.52]
Zhou Z 2010	-1.66	0.52564246	39	-1.77	0.54249424	39	5.7%	0.11 [-0.13, 0.35]
**Subtotal (95% CI)**			**202**			**202**	**21.6%**	**-0.04 [-0.26, 0.17]**
Heterogeneity: Tau² = 0.03; Chi² = 10.76, df = 3 (P = 0.01); I² = 72%								
Test for overall effect: Z = 0.39 (P = 0.70)								
**5.12.3 Pitavastatin**								
Qu J 2020	-1.34	0.59573484	39	-0.92	0.64505814	39	5.4%	-0.42 [-0.70, -0.14]
Tian L 2022	-1.34	0.67668309	30	-0.71	0.63316664	30	4.9%	-0.63 [-0.96, -0.30]
**Subtotal (95% CI)**			**69**			**69**	**10.3%**	**-0.51 [-0.72, -0.29]**
Heterogeneity: Tau² = 0.00; Chi² = 0.91, df = 1 (P = 0.34); I² = 0%								
Test for overall effect: Z = 4.68 (P < 0.00001)								
**Total (95% CI)**			**1074**			**1074**	**100.0%**	**-0.34 [-0.47, -0.20]**
Heterogeneity: Tau² = 0.07; Chi² = 162.51, df = 17 (P < 0.00001); I² = 90%								
Test for overall effect: Z = 4.96 (P < 0.00001)								
Test for subgroup differences: Chi² = 10.28, df = 2 (P = 0.006), I² = 80.5%								

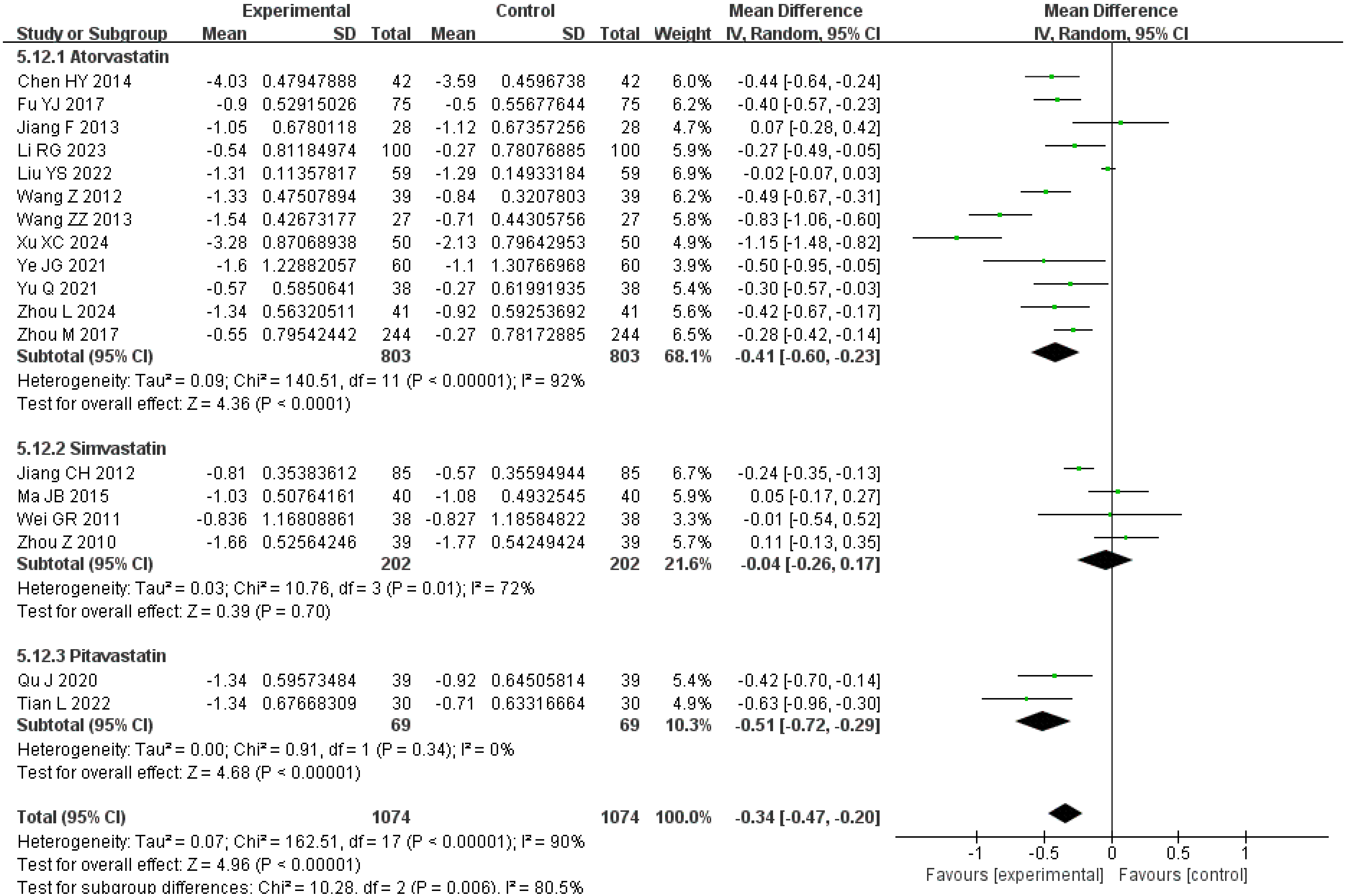

S9. Sensitivity analysis for different outcomes.

Effect size by leave-one-out method	TT+DTJZ	TT+JZL	TT+JZTL	TT+JZTM	TT+PS	TT+SLXMK	TT+XZK	TT+XZT
Clinical total effective rate	3.05 (1.79, 5,17)	5.08 (1.38, 18.71)	4.20 (1.81, 9.77)	5.24 (2.22, 12.37)	3.37 (1.98, 5.73)	3.26 (2.19, 4.87)	3.97 (3.03, 5.22)	3.57 (1.78, 7.15)
TC	-0.95 (-1.38, -0.51)	-0.73 (-0.98, -0.48)	-0.49 (-0.85, -0.14)	-0.69 (-0.96, -0.41)	-0.14 (-0.23, -0.05)	-0.79 (-1.20, -0.37)	-0.60 (-0.78, -0.42)	-0.70 (-1.02, -0.37)
TG	-0.49 (-0.69, -0.28)	-0.49 (-0.76, -0.21)	-0.49 (-0.70, -0.28)	-0.48 (-0.86, -0.10)	-0.59 (-0.81, -0.36)	-0.36 (-0.66, -0.07)	-0.34 (-0.46, -0.23)	-0.47 (-0.67, -0.27)
HDL-C	0.33 (0.16, 0.51)	0.37 (0.17, 0.58)	0.26 (0.03, 0.49)	0.38 (0.10, 0.67)	0.31 (0.10, 0.51)	0.21 (0.02, 0.40)	0.21 (0.17, 0.25)	0.27 (0.18, 0.37)
LDL-C	-0.61 (-0.98, -0.23)	-0.65 (-0.91, -0.39)	-0.41 (-0.88, 0.05)	-0.32 (-0.49, -0.16)	-0.11 (-0.22, 0.00)	-0.31 (-0.73, 0.10)	-0.38 (-0.52, -0.24)	-0.50 (-0.70, -0.31)

S10. Publication bias analysis.

Comparisons	Outcomes	RCTs	Begg's test	Egger's test	Trim and fill	
			p	p	Left	Right
TT+DTJZ vs TT	Clinical total effective rate	4	1	0.343	2	
TT+JZTM vs TT	Clinical total effective rate	3	0.296	**0.048**	0	
TT+PS vs TT	Clinical total effective rate	6	0.26	0.166	0	
TT+SLXMK vs TT	Clinical total effective rate	5	0.806	0.424	1	
TT+XZK vs TT	Clinical total effective rate	20	0.194	**0.011**	8	
TT+XZT vs TT	Clinical total effective rate	4	0.308	0.189	2	
TT+DTJZ vs TT	TC	5	1	0.767		0
TT+JZL vs TT	TC	4	0.308	0.235		1
TT+JZTL vs TT	TC	4	0.734	0.333		1
TT+JZTM vs TT	TC	5	0.462	0.213	0	
TT+PS vs TT	TC	10	1	0.991	1	
TT+SLXMK vs TT	TC	4	0.308	0.102	0	
TT+XZK vs TT	TC	24	0.785	0.871		0
TT+XZT vs TT	TC	6	1	0.988		1
TT+DTJZ vs TT	TG	5	1	0.459		0
TT+JZL vs TT	TG	4	0.308	0.58	0	
TT+JZTL vs TT	TG	4	0.734	0.686	0	
TT+JZTM vs TT	TG	5	0.806	0.135		2
TT+PS vs TT	TG	10	0.721	0.647	0	
TT+SLXMK vs TT	TG	4	0.734	0.409		1
TT+XZK vs TT	TG	23	0.635	0.452	5	
TT+XZT vs TT	TG	6	0.06	**0.002**		2
TT+DTJZ vs TT	HDL-C	5	0.221	0.442	0	
TT+JZL vs TT	HDL-C	4	0.734	0.277	0	
TT+JZTL vs TT	HDL-C	4	0.308	0.077	1	
TT+JZTM vs TT	HDL-C	5	1	0.417	0	
TT+PS vs TT	HDL-C	10	1	0.7	0	
TT+SLXMK vs TT	HDL-C	4	0.734	0.587	0	
TT+XZK vs TT	HDL-C	22	0.259	0.265	0	
TT+XZT vs TT	HDL-C	6	0.452	0.646		0
TT+DTJZ vs TT	LDL-C	4	0.734	0.777	0	
TT+JZL vs TT	LDL-C	4	0.308	0.121	1	
TT+JZTL vs TT	LDL-C	3	1	0.56	0	
TT+JZTM vs TT	LDL-C	5	1	0.408	0	
TT+PS vs TT	LDL-C	10	0.107	0.346		2
TT+SLXMK vs TT	LDL-C	3	0.296	0.338		0
TT+XZK vs TT	LDL-C	21	0.695	**0.028**		0
TT+XZT vs TT	LDL-C	6	1	0.762	2	

S11. GRADE assessment.

S11.1 GRADE assessment of clinical total effective rate.

	Certainly assessment						No. of patients			
Intervention	No. of studies	Risk of bias	Inconsistency	Indirectness	Imprecision	Publication bias	TT+TCMs	TT	Effect size	Certainty
TT+DTJZ	4	not serious	not serious	not serious	not serious	none	286/307 (93.2%)	251/307 (81.8%)	OR 3.05 (1.79 to 5.17)	⊕⊕⊕⊕ High
TT+JZL	2	not serious	not serious	not serious	serious[b]	none	71/74 (95.9%)	61/74 (82.4%)	OR 5.08 (1.38 to 18.71)	⊕⊕⊕◯ Moderate[b]
TT+JZTL	2	not serious	not serious	not serious	serious[b]	none	103/111 (92.8%)	84/111 (75.7%)	OR 4.20 (1.81 to 9.77)	⊕⊕⊕◯ Moderate[b]
TT+JZTM	3	serious[a]	not serious	not serious	not serious	publication bias strongly suspected[c]	129/136 (94.9%)	106/136 (77.9%)	OR 5.24 (2.22 to 12.37)	⊕⊕◯◯ Low[a,c]
TT+PS	6	not serious	not serious	not serious	not serious	none	195/217 (89.9%)	167/232 (72.0%)	OR 3.37 (1.98 to 5.73)	⊕⊕⊕⊕ High
TT+SLXMK	5	serious[a]	not serious	not serious	not serious	none	458/496 (92.3%)	388/490 (79.2%)	OR 3.26 (2.19 to 4.87)	⊕⊕⊕◯ Moderate[a]
TT+XZK	20	not serious	not serious	not serious	not serious	publication bias strongly suspected[c]	1148/1227 (93.6%)	968/1221 (79.3%)	OR 3.97 (3.03 to 5.22)	⊕⊕⊕◯ Moderate[c]
TT+XZT	4	not serious	not serious	not serious	not serious	none	158/170 (92.9%)	134/170 (78.8%)	OR 3.57 (1.78 to 7.15)	⊕⊕⊕⊕ High

a. High risk of randomization process or outcome data missing.
b. The sample size included in the outcome is too small.
c. Publication bias is probably.

S11.2 GRADE assessment of TC.

	Certainly assessment						No. of patients			
Intervention	No. of studies	Risk of bias	Inconsistency	Indirectness	Imprecision	Publication bias	TT+TCMs	TT	Effect size	Certainty
TT+DTJZ	5	not serious	serious[b]	not serious	not serious	none	337	337	MD -0.95 (-1.38 to -0.51)	⊕⊕⊕◯ Moderate[b]
TT+JZL	4	not serious	not serious	not serious	not serious	none	172	174	MD -0.73 (-0.98 to -0.48)	⊕⊕⊕⊕ High
TT+JZTL	4	serious[a]	serious[b]	not serious	not serious	none	229	231	MD -0.49 (-0.85 to -0.14)	⊕⊕◯◯ Low[a,b]
TT+JZTM	5	not serious	serious[b]	not serious	not serious	none	181	181	MD -0.69 (-0.96 to -0.41)	⊕⊕⊕◯ Moderate[b]
TT+PS	10	not serious	not serious	not serious	not serious	none	377	357	MD -0.14 (-0.23 to -0.05)	⊕⊕⊕⊕ High

TT+SLXMK	4	not serious	serious[b]	not serious	not serious	none	202	194	MD -0.79 (-1.2 to -0.37)	⊕⊕⊕◯ Moderate[b]
TT+XZK	24	not serious	serious[b]	not serious	not serious	none	1400	1395	MD -0.6 (-0.78 to -0.42)	⊕⊕⊕◯ Moderate[b]
TT+XZT	6	not serious	not serious	not serious	not serious	none	290	290	MD -0.7 (-1.02 to -0.37)	⊕⊕⊕⊕ High

a. High risk of randomization process or outcome data missing.
b. Heterogeneity among included studies ($I^2 \geq 50\%$).

S11.3 GRADE assessment of TG.

		Certainly assessment					No. of patients			
Intervention	No. of studies	Risk of bias	Inconsistency	Indirectness	Imprecision	Publication bias	TT+TCMs	TT	Effect size	Certainty
TT+DTJZ	5	not serious	not serious	not serious	not serious	none	337	337	MD -0.49 (-0.69 to -0.28)	⊕⊕⊕⊕ High
TT+JZL	4	not serious	not serious	not serious	not serious	none	172	174	MD -0.49 (-0.76 to -0.21)	⊕⊕⊕⊕ High
TT+JZTL	4	serious[a]	not serious	not serious	not serious	none	229	231	MD -0.49 (-0.7 to -0.28)	⊕⊕⊕◯ Moderate[a]
TT+JZTM	5	not serious	not serious	not serious	not serious	none	181	181	MD -0.48 (-0.86 to -0.1)	⊕⊕⊕⊕ High
TT+PS	10	not serious	serious[b]	not serious	not serious	none	377	357	MD -0.59 (-0.81 to -0.36)	⊕⊕⊕◯ Moderate[b]
TT+SLXMK	4	not serious	serious[b]	not serious	not serious	none	202	194	MD -0.36 (-0.66 to -0.07)	⊕⊕⊕◯ Moderate[b]
TT+XZK	23	not serious	serious[b]	not serious	not serious	none	1361	135 6	MD -0.34 (-0.46 to -0.23)	⊕⊕⊕◯ Moderate[b]
TT+XZT	6	not serious	not serious	not serious	not serious	publication bias strongly suspected[c]	290	290	MD -0.47 (-0.67 to -0.27)	⊕⊕⊕◯ Moderate[c]

a. High risk of randomization process or outcome data missing.
b. Heterogeneity among included studies ($I^2 \geq 50\%$).
c. Publication bias is probably.

S11.4 GRADE assessment of HDL-C.

Certainly assessment							No. of patients		Effect size	Certainty
Intervention	No. of studies	Risk of bias	Inconsistency	Indirectness	Imprecision	Publication bias	TT+TCMs	TT		
TT+DTJZ	5	not serious	serious[b]	not serious	not serious	none	337	337	MD 0.33 (0.16 to 0.51)	⊕⊕⊕◯ Moderate[b]
TT+JZL	4	not serious	serious[b]	not serious	not serious	none	172	174	MD 0.37 (0.17 to 0.58)	⊕⊕⊕◯ Moderate[b]
TT+JZTL	4	serious[a]	serious[b]	not serious	not serious	none	229	231	MD 0.26 (0.03 to 0.49)	⊕⊕◯◯ Low[a,b]
TT+JZTM	5	not serious	serious[b]	not serious	not serious	none	181	181	MD 0.38 (0.1 to 0.67)	⊕⊕⊕◯ Moderate[b]
TT+PS	10	not serious	serious[b]	not serious	not serious	none	377	357	MD 0.31 (0.1 to 0.51)	⊕⊕⊕◯ Moderate[b]
TT+SLXMK	4	not serious	serious[b]	not serious	not serious	none	202	194	MD 0.21 (0.02 to 0.4)	⊕⊕⊕◯ Moderate[b]
TT+XZK	22	not serious	serious[b]	not serious	not serious	none	1334	1334	MD 0.21 (0.17 to 0.25)	⊕⊕⊕◯ Moderate[b]
TT+XZT	6	not serious	serious[b]	not serious	not serious	none	290	290	MD 0.27 (0.18 to 0.37)	⊕⊕⊕◯ Moderate[b]

a. High risk of randomization process or outcome data missing.
b. Heterogeneity among included studies ($I^2 \geq 50\%$).

S11.5 GRADE assessment of LDL-C.

Certainly assessment							No. of patients		Effect size	Certainty
Intervention	No. of studies	Risk of bias	Inconsistency	Indirectness	Imprecision	Publication bias	TT+TCMs	TT		
TT+DTJZ	4	not serious	not serious	not serious	not serious	none	179	179	MD -0.61 (-0.98 to -0.23)	⊕⊕⊕⊕ High
TT+JZL	4	not serious	serious[b]	not serious	not serious	none	172	174	MD -0.65 (-0.91 to -0.39)	⊕⊕⊕◯ Moderate[b]
TT+JZTL	3	serious[a]	serious[b]	not serious	not serious	none	173	175	MD -0.41 (-0.88 to 0.05)	⊕⊕◯◯ Low[a,b]
TT+JZTM	5	not serious	not serious	not serious	not serious	none	181	181	MD -0.32 (-0.49 to -0.16)	⊕⊕⊕⊕ High
TT+PS	10	not serious	serious[b]	not serious	not serious	none	377	357	MD -0.11 (-0.22 to 0)	⊕⊕⊕◯ Moderate[b]
TT+SLXMK	3	not serious	serious[b]	not serious	not serious	none	139	137	MD -0.31 (-0.73 to 0.1)	⊕⊕⊕◯ Moderate[b]
TT+XZK	21	not serious	serious[b]	not serious	not serious	publication bias strongly suspected[c]	1274	1274	MD -0.38 (-0.52 to -0.24)	⊕⊕◯◯ Low[b,c]

TT+XZT	6	not serious	not serious	not serious	not serious	none	290	290	MD -0.5 (-0.7 to -0.31)	⊕⊕⊕⊕ High

a. High risk of randomization process or outcome data missing.
b. Heterogeneity among included studies ($I^2 \geq 50\%$).
c. Publication bias is probably.

S12. Forest plot of safety outcomes.

Study or Subgroup	Experimental Events	Experimental Total	Control Events	Control Total	Weight	Odds Ratio M-H, Random, 95% CI
**6.10.1 TT+DTJZ**						
Liang ZM 2015	1	56	3	56	1.7%	0.32 [0.03, 3.19]
Wang QC 2012	1	30	0	30	0.9%	3.10 [0.12, 79.23]
**Subtotal (95% CI)**		**86**		**86**	**2.6%**	**0.74 [0.09, 6.31]**
Total events	2		3			
Heterogeneity: Tau^2 = 0.52; Chi^2 = 1.25, df = 1 (P = 0.26); I^2 = 20%						
Test for overall effect: Z = 0.28 (P = 0.78)						
**6.10.2 TT+JZL**						
Zhang XL 2017	3	50	3	50	2.8%	1.00 [0.19, 5.21]
**Subtotal (95% CI)**		**50**		**50**	**2.8%**	**1.00 [0.19, 5.21]**
Total events	3		3			
Heterogeneity: Not applicable						
Test for overall effect: Z = 0.00 (P = 1.00)						
**6.10.3 TT+JZTL**						
Bao FY 2009	8	56	6	56	4.7%	1.39 [0.45, 4.30]
Guo YW 2017	2	58	1	58	1.5%	2.04 [0.18, 23.09]
Wen CT 2013	4	55	5	55	3.7%	0.78 [0.20, 3.09]
Xie Y 2014	6	60	11	62	5.0%	0.52 [0.18, 1.50]
**Subtotal (95% CI)**		**229**		**231**	**14.9%**	**0.87 [0.45, 1.66]**
Total events	20		23			
Heterogeneity: Tau^2 = 0.00; Chi^2 = 2.08, df = 3 (P = 0.56); I^2 = 0%						
Test for overall effect: Z = 0.43 (P = 0.67)						
**6.10.4 TT+JZTM**						
Lu HM 2021	6	46	4	46	3.8%	1.57 [0.41, 6.00]
Wang H 2019	1	15	1	15	1.1%	1.00 [0.06, 17.62]
You HS 2022	3	50	2	50	2.4%	1.53 [0.24, 9.59]
**Subtotal (95% CI)**		**111**		**111**	**7.4%**	**1.48 [0.54, 4.06]**
Total events	10		7			
Heterogeneity: Tau^2 = 0.00; Chi^2 = 0.08, df = 2 (P = 0.96); I^2 = 0%						
Test for overall effect: Z = 0.75 (P = 0.45)						
**6.10.5 TT+PS**						
Cao CY 2014	1	42	1	42	1.2%	1.00 [0.06, 16.53]
Cao XC 2021	2	32	1	32	1.5%	2.07 [0.18, 24.01]
Chen X 2016	2	20	2	20	2.0%	1.00 [0.13, 7.89]
Li X 2016	1	30	3	46	1.7%	0.49 [0.05, 4.99]
Mu M 2014	6	43	5	43	4.1%	1.23 [0.35, 4.39]
**Subtotal (95% CI)**		**167**		**183**	**10.4%**	**1.09 [0.46, 2.60]**
Total events	12		12			
Heterogeneity: Tau^2 = 0.00; Chi^2 = 0.76, df = 4 (P = 0.94); I^2 = 0%						
Test for overall effect: Z = 0.20 (P = 0.84)						
**6.10.6 TT+SLXMK**						
Cai ZQ 2015	5	48	4	48	3.7%	1.28 [0.32, 5.09]
**Subtotal (95% CI)**		**48**		**48**	**3.7%**	**1.28 [0.32, 5.09]**
Total events	5		4			
Heterogeneity: Not applicable						
Test for overall effect: Z = 0.35 (P = 0.73)						
**6.10.7 TT+XZK**						
Chen HY 2014	3	42	5	42	3.3%	0.57 [0.13, 2.55]
Fu YJ 2017	5	74	4	75	3.8%	1.29 [0.33, 4.99]
Hu GY 2015	6	60	7	60	4.6%	0.84 [0.27, 2.67]
Li RG 2023	2	100	12	100	3.2%	0.15 [0.03, 0.69]
Ma JB 2015	1	40	8	40	1.9%	0.10 [0.01, 0.86]
Qiang SC 2023	5	90	13	90	5.0%	0.35 [0.12, 1.02]
Shi C 2018	2	62	5	62	2.8%	0.38 [0.07, 2.04]
Wang Z 2012	1	39	8	39	1.9%	0.10 [0.01, 0.86]
Wei GR 2011	3	38	10	38	3.7%	0.24 [0.06, 0.96]
Xu XC 2024	5	50	3	50	3.3%	1.74 [0.39, 7.71]
Ye JG 2021	8	60	7	60	4.9%	1.16 [0.39, 3.44]
Yu Q 2021	1	38	6	38	1.8%	0.14 [0.02, 1.26]
Zhou L 2024	5	41	3	41	3.3%	1.76 [0.39, 7.90]
Zhou M 2017	0	244	34	244	1.2%	0.01 [0.00, 0.20]
**Subtotal (95% CI)**		**978**		**979**	**44.5%**	**0.43 [0.23, 0.79]**
Total events	47		125			
Heterogeneity: Tau^2 = 0.75; Chi^2 = 29.90, df = 13 (P = 0.005); I^2 = 57%						
Test for overall effect: Z = 2.69 (P = 0.007)						
**6.10.8 TT+XZT**						
Chen Q 2022	3	30	4	30	3.0%	0.72 [0.15, 3.54]
Kuang ZB 2010	7	60	7	60	4.8%	1.00 [0.33, 3.05]
Shi WH 2011	0	60	7	60	1.1%	0.06 [0.00, 1.06]
Xiong G 2019	0	50	3	50	1.1%	0.13 [0.01, 2.67]
Yang DM 2014	5	40	4	40	3.6%	1.29 [0.32, 5.19]
**Subtotal (95% CI)**		**240**		**240**	**13.6%**	**0.66 [0.27, 1.63]**
Total events	15		25			
Heterogeneity: Tau^2 = 0.29; Chi^2 = 5.53, df = 4 (P = 0.24); I^2 = 28%						
Test for overall effect: Z = 0.89 (P = 0.37)						
**Total (95% CI)**		**1909**		**1928**	**100.0%**	**0.69 [0.50, 0.95]**
Total events	114		202			
Heterogeneity: Tau^2 = 0.25; Chi^2 = 46.97, df = 34 (P = 0.07); I^2 = 28%						
Test for overall effect: Z = 2.27 (P = 0.02)						
Test for subgroup differences: Chi^2 = 6.55, df = 7 (P = 0.48), I^2 = 0%						

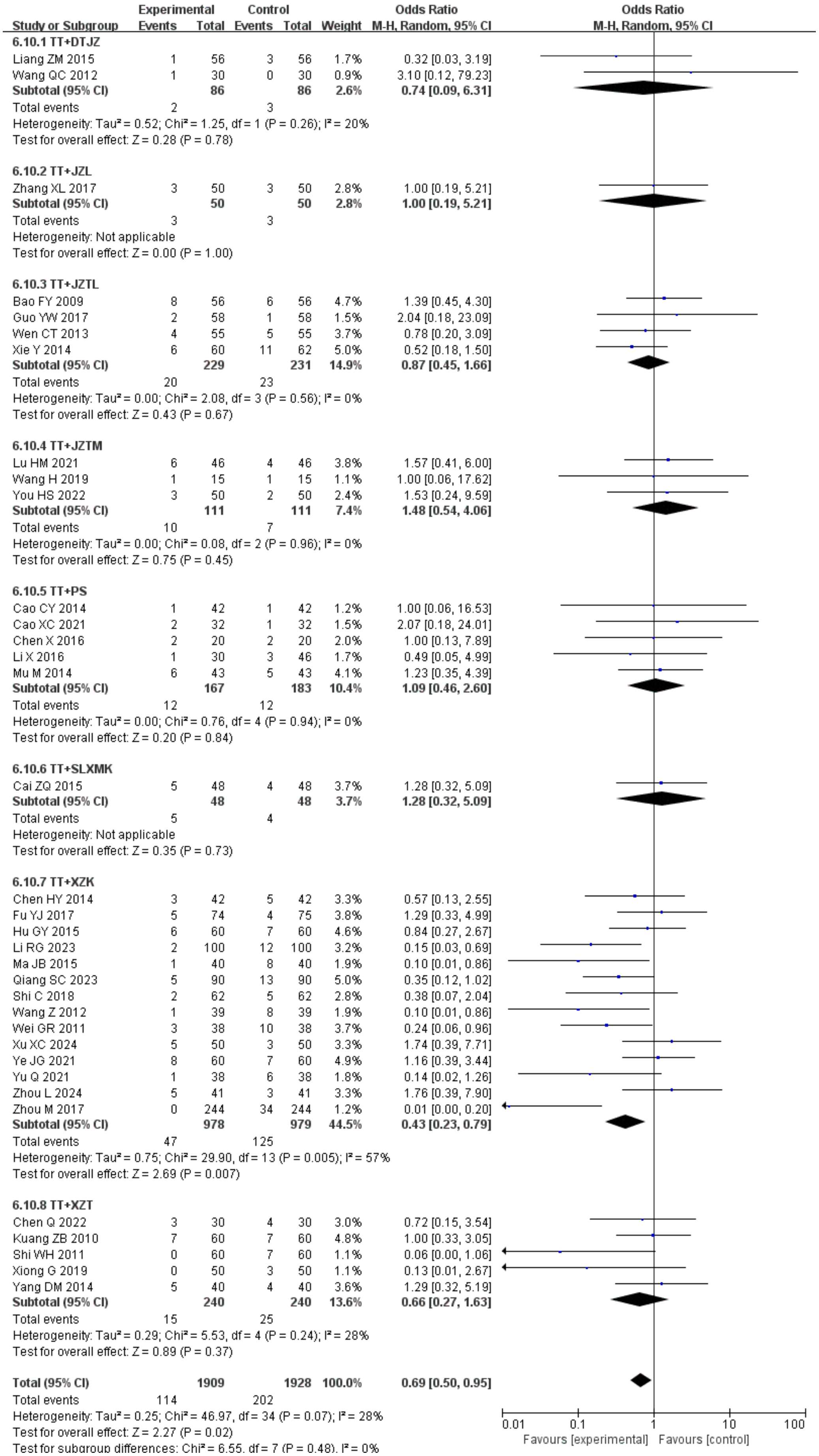

S13. Summary table of adverse reactions.

Study ID	Adverse reactions	
	T	C
Wang QC 2012[1]	Abdominal distension and pain in 1 case	None
Liang ZM 2015[5]	Nausea and vomiting in 1 case	Nausea and vomiting in 3 cases
Zhang XL 2017[6]	Elevated liver and kidney function indicators in 3 cases (elevated ALT in 1 case, elevated AST in 2 cases, elevated ALP in 1 case)	Elevated liver and kidney function indicators in 3 cases (elevated ALT in 1 case, elevated AST in 1 case)
Bao FY 2009[10]	Gastrointestinal discomfort reactions and increased liver and kidney indicators in 8 cases (including nausea, abdominal bloating, diarrhea, increased ALT and AST)	Gastrointestinal discomfort reactions and increased liver and kidney indicators in 6 cases (including nausea, abdominal bloating, diarrhea, increased ALT and AST).
Xie Y 2014[11]	Mildly elevated ALT in 3 cases, mildly elevated AST in 1 case, mildly elevated CK in 1 case, abdominal discomfort in 1 case.	Mildly elevated ALT in 5 cases, mildly elevated AST in 4 cases, mildly elevated CK in 1 case, abdominal discomfort in 1 case.
Guo YW 2017[12]	Abdominal bloating in 1 case, fatigue in 1 case.	Mildly elevated ALT in 1 case
Wen CT 2013[13]	Stomach pain and diarrhea in 1 case, limb itching in 1 case, AST elevation in 1 case, CK elevation in 1 case.	Dizziness in 1 case, abdominal pain in 1 case, AST elevation in 2 cases, CK elevation in 1 case.
Lu HM 2021[16]	Headache in 1 case, vomiting in 3 cases, nausea in 2 cases	Headache in 1 case, vomiting in 2 cases, nausea in 1 case
You HS 2022[17]	Nausea in 1 case, vomiting in 1 case, muscle soreness in 1 case	Nausea in 1 case, muscle soreness in 1 case
Wang H 2019[18]	Mildly elevated ALT in 1 case	Mildly elevated ALT in 1 case
Li X 2016[21]	Abdominal bloating and diarrhea in 1 case	Abdominal bloating and diarrhea in 3 cases
Chen X 2016[22]	Mildly elevated ALT/AST in 2 cases	Mildly elevated ALT/AST in 2 cases
Cao CY 2014[23]	Gastrointestinal discomfort reaction in 1 case	Lower limb weakness in 1 case
Cao XC 2021[25]	Mildly elevated liver function indicators in 2 cases	Mildly elevated liver function indicators in 1 case
Mu M 2014[26]	Diarrhea and constipation in 4 cases, mildly elevated liver function indicators in 2 cases	Abdominal discomfort in 3 cases, mildly elevated liver function indicators in 2 cases
Cai ZQ 2015[32]	Diarrhea in 3 cases, abdominal bloating in 2 cases	Abdominal bloating and constipation in 4 cases
Zhou M 2017[38]	None	Myalgia, joint swelling, and muscle fatigue in a total of 34 cases
Ye JG 2021[39]	Nausea in 2 cases, abdominal bloating in 3 cases, fatigue in 3 cases	Nausea in 2 cases, abdominal bloating in 3 cases, fatigue in 2 cases
Yu Q 2021[40]	Joint swelling in 1 case	Myalgia in 2 cases, joint swelling in 3 cases, muscle fatigue in 1 case
Shi C 2018[41]	Nausea and vomiting in 2 cases	Nausea and vomiting in 3 cases, abdominal bloating in 2 cases
Wei GR 2011[50]	Nausea in 2 cases, dizziness and headache in 1 case	Nausea in 4 cases, headache and dizziness in 3 cases, rash in 2 cases, myalgia in 1 case

Chen HY 2014[51]	Gastrointestinal reaction, mildly elevated liver and kidney function indicators in 3 cases	Gastrointestinal reaction, mildly elevated liver and kidney function indicators in 5 cases
Hu GY 2015[53]	Nausea, diarrhea, and other gastrointestinal discomfort reactions in 6 cases	Abdominal bloating, fatigue, and diarrhea in 7 cases
Wang Z 2012[54]	Abdominal bloating in 1 case	Gastrointestinal discomfort reaction in 8 cases
Ma JB 2015[56]	Gastrointestinal discomfort reaction in 1 case	Gastrointestinal discomfort reaction in 6 cases, liver and kidney indicators elevated in 2 cases
Li RG 2023[57]	Digestive tract reaction in 2 cases	Liver function abnormality in 2 cases, digestive tract reaction in 10 cases
Zhou L 2024[58]	Muscle pain in 2 cases, nausea in 2 cases, vomiting in 1 case	Muscle pain in 1 case, nausea in 1 case, vomiting in 1 case
Fu YJ 2017[59]	Diarrhea and facial flushing each in 2 cases, nausea and vomiting in 1 case	Diarrhea in 2 cases, nausea and vomiting, renal function abnormality each in 1 case
Xu XC 2024[60]	Nausea and vomiting in 1 case, muscle pain in 2 cases, diarrhea and abdominal pain in 2 cases	Nausea and vomiting in 1 case, myalgia in 1 case, diarrhea and abdominal pain in 1 case
Qiang SC 2023[61]	Dizziness and headache in 1 case, nausea in 2 cases, constipation in 1 case, abdominal bloating in 1 case	Dizziness and headache in 3 cases, nausea in 3 cases, constipation in 3 cases, abdominal bloating in 1 case, skin itching in 1 case, liver function damage in 2 cases
Shi WH 2011[62]	None	Nausea and diarrhea in 4 cases, myalgia in 2 cases, rash in 1 case
Chen Q 2022[64]	Abdominal bloating in 2 cases, nausea in 1 case	Abdominal bloating in 3 cases, vomiting in 1 case
Yang DM 2014[65]	Diarrhea in 4 cases, dizziness and headache in 1 case	Nausea, abdominal discomfort in 3 cases, taste disturbance in 1 case
Xiong G 2019[66]	None	Mildly elevated liver and kidney indicators in 1 case, abdominal bloating, no nausea or vomiting in 2 cases
Kuang ZB 2010[67]	Constipation in 3 cases, abdominal pain in 2 cases, nausea in 2 cases	Constipation in 4 cases, abdominal pain in 1 case, nausea in 2 cases
